# Inoculation strategies affect the physicochemical properties and flavor of Zhenjiang aromatic vinegar

**DOI:** 10.3389/fmicb.2023.1126238

**Published:** 2023-03-09

**Authors:** Xiaoting Ye, Yongjian Yu, Jiaxin Liu, Yuanyuan Zhu, Zhen Yu, Peng Liu, Yuqin Wang, Ke Wang

**Affiliations:** School of Grain Science and Technology, Jiangsu University of Science and Technology, Zhenjiang, Jiangsu, China

**Keywords:** Zhenjiang aromatic vinegar, inoculation strategy, fermentation, flavor quality, correlation analysis

## Abstract

Inoculation strategy is a significant determinant of the flavor quality of Zhenjiang aromatic vinegar. Herein, the comparative analyses of the effects of various inoculation strategies on the physicochemical properties, microbial community structure, and flavoring characteristics of Zhenjiang aromatic vinegar were performed. The results showed that the contents of total acid (6.91 g/100 g), organic acid (2099.63 ± 4.13 mg/100 g) and amino acid (3666.18 ± 14.40 mg/100 g) in the direct inoculation strategy were higher than those in the traditional inoculation strategy (6.21 ± 0.02 g/100 g, 1939.66 ± 4.16 mg/100 g and 3301.46 ± 13.41 mg/100 g). At the same time, it can effectively promote the production of acetoin. The diversity of strains under the traditional inoculation strategy was higher than that under the direct inoculation strategy, and the relative abundance of major microbial genera in the fermentation process was lower than that under the direct inoculation strategy. In addition, for two different inoculation strategies, pH was proved to be an important environmental factor affecting the microbial community structure during acetic acid fermentation. The correlation between main microbial species, organic acids, non-volatile acids, and volatile flavor compounds is more consistent. Therefore, this study may help to develop direct injection composite microbial inoculants to replace traditional starter cultures in future research.

## 1. Introduction

Zhenjiang vinegar is one of the four famous vinegar in China, and it is always a popular acidic condiment with a long history (Jin et al., 2017). Due to the seasonal changes in the process of open fermentation, the diversity of raw materials and the complexity of microorganisms, the internal and external environmental factors of vinegar fermentation have changed, so the flavor quality of traditional solid-state fermentation vinegar is unstable (Jin et al., 2017). Acetic acid fermentation is the most important stage in the process of vinegar brewing, and also the decisive factor of vinegar flavor quality (Wu et al., 2021). Traditional solid-state vinegar fermentation is carried out through spontaneous fermentation of raw materials and complex microbial communities in the environment. Under open conditions, a series of complex microbial groups produce rich metabolic products through fermentation to create Zhenjiang aromatic vinegar flavor with unique characteristics, such as smooth, aromatic, mild sweetness, rich color, and fresh taste.

Because the quality of vinegar produced by traditional inoculation is mostly adjusted by experience, the metabolic mechanism and environmental parameters of complex fermentation microbial population cannot be controlled. This leads to low output and unstable quality of products, which seriously hinders technological progress and expansion of production scale. Therefore, the study of the relationship between microbiota and the formation of flavoring substances is important for understanding, controlling and effectively improving vinegar fermentation to improve food quality, productivity and safety. The rapid development of molecular biology and modern detection technology has gradually revealed the contribution of core microorganisms and functional microflora to the flavor of fermented products. In addition, breeding excellent functional strains and adjusting microbial community function through direct inoculation to improve fermentation efficiency are one of the most effective measures to improve yield and quality. Compared with traditional inoculation strategies, microbial community with selective species proportion is simplified and fermentation is easier to control (Wolfe and Dutton, 2015).

The direct inoculation strategy refers to the use of core microorganisms or functional microorganisms as a direct-injection starter (DVS) to replace the traditional fermented and mature seed grains in fermentation production. Under the same conditions, compared with the traditional inoculation strategy, the direct injection inoculation strategy can strengthen the adaptability and stability of the functional bacteria directly inoculated in the fermentation system, and effectively improve the product quality. Moreover, it can be applied in food and pharmaceutical fields (Shi et al., 2022). Relevant research indicates that using *Aspergillus niger*-enhanced gluten meal in the production of Baoning Vinegar can increase the total acid and organic acid contents as well as the utilization rate of starch and maintain relatively stable quality throughout the three cycles of fermentation (Liu et al., 2020). Lactobacillus sp. and *Acetobacter pasteurianus* were used as direct-dose microbial inoculum for *in-situ* biological coculture, and they could synergistically promote the accumulation of key components including acetoin (Shi et al., 2022). Additionally, non-rich species *Komagataeibacter europaeus* is applied to the fermentation of Zhenjiang aromatic vinegar, which can effectively regulate the composition of bacteria, strengthen the *in situ* symbiotic network, and up-regulate the abundance of genes related to sugar and alcohol metabolism (Lu et al., 2018; Peng et al., 2021). Therefore, the direct inoculation strategy can theoretically stabilize the flavor quality of the product and preserve the richness of flavoring substances in the traditional solid-state fermented vinegar, which is an effective strategy to regulate the microbial community and its functions and improve the quality of traditional Chinese grain vinegar.

Therefore, acetic acid and lactic acid bacteria, the key microorganisms in the production of Zhenjiang vinegar, were introduced into the solid-state fermentation production as direct inoculants. The effects of two different inoculation strategies on the physicochemical properties (total acid, reducing sugar and non-volatile acid), microbial community structure and flavor characteristics (organic acid, free amino acid and aroma) of Zhenjiang vinegar were compared. In addition, the effects of two inoculation strategies on microbial communities and metabolic components, and their differences in contribution to vinegar flavor were studied. The purpose of this study was to explore the effects of two inoculation strategies on the physicochemical indexes of Zhenjiang vinegar during acetic acid fermentation, and to provide theoretical support for the combination of traditional and modern vinegar brewing methods and the adjustment of technological parameters in large-scale production.

## 2. Materials and methods

### 2.1. Preparation of samples

In this study, Zhenjiang aromatic vinegar was brewed by traditional solid-state fermentation process. The natural seed fermented grains inoculated on the seventh day of fermentation (provided by Jiangsu Hengshun Vinegar Co., Ltd.) and artificially pure cultured strains (*Acetobacter* A-1 and Lactic Acid Bacteria Y-14) of Zhenjiang aromatic vinegar were used as research subjects. The marinade samples collected on days 0, 2, 4, 6, 8, 10, 12, 14, 16, and 18 of the fermentation were sampled daily before the rotation of the fermented grains, with three replicates for each sample. The vinegar was extracted from the fermentation tank from top to bottom, and 100 g of it was weighed in a self-sealing bag after uniform mixing. A 30-mL sample of marinade was transferred from the bottom of the cylinder to a 50-mL centrifuge tube and stored at −20°C. Samples were thawed in a water bath at room temperature (26°C to 30°C) before analysis.

### 2.2. Reagents

Sodium hydroxide, potassium hydrogen phthalate, glucose monohydrate, 99% anhydrous sodium phosphate monohydrate, potassium hexacyanoferrate trihydrate, zinc sulfate heptahydrate, ethanol, phosphoric acid, phenolphthalein indicator, high-performance liquid chromatography grade methanol, boric acid, lithium chloride, lithium hydroxide-monohydrate, 4-methyl-2-pentanol, citric acid, hydrochloric acid, octanoic acid, potassium acetate, sodium acetate trihydrate, acetic acid, sodium chloride, ninhydrin crystal, phenol, and ascorbic acid were purchased from Guoyao Group Chemical Reagent Co., Ltd (Shanghai, China). Citric acid (≥99.5% purity), L-pyroglutamic acid (≥99% purity), lactic acid (≥99% purity), acetic acid (≥99% purity), succinic acid (≥99% purity), and malic acid (≥99% purity) were purchased from McLean Biochemical Technology Co., Ltd (Shanghai, China).

### 2.3. Analysis of physical and chemical properties

The temperature during the Fermentation was monitored using a thermometer. A total of 10 g of the vinegar *pei* was added into 30 ml distilled water, agitated at 100 rpm for 3 h at room temperature (20°C), centrifuged for 10 min at 6000 g, and the supernatant collected for the following analysis. The total acid, pH, amino nitrogen, and reducing sugar contents were determined following the previous methods (Huang et al., 2022). The contents of nine organic acids were analyzed by an Agilent 1,260 high-performance liquid chromatography (HPLC) system (Agilent Corp., Karlsruhe, Germany) with Aminex HPX-87H ion exclusion column (7.8 × 300 mm, i.d., 5 μM) according to the previous research (Wu et al., 2017). The amino acid content was measured using a Sykam S-433D amino acid analyzer (Sykam GmbH, Bavaria, Germany) (Yang et al., 2021). The reducing sugar content was measured by the 3,5-dinitrosalicylic acid (DNS) method (Yang et al., 2018). The contents of volatile compounds were detected by headspace-solid phase microextraction (HS-SPME)/gas chromatography–mass spectrometry (GC–MS) using an Agilent 7890B-5977B GC–MSD system equipped with a DB-wax capillary column (30.0 m × 0.25 mm × 0.25 μm, Agilent Technology, Santa Clara, CA, United States; Jia et al., 2020). All measurements were carried out in triplicates.

### 2.4. Microbial community analysis

Samples of vinegar *pei* and the precipitates was prepared on the basis of the previous method and the DNA of the sample was extracted (Wu et al., 2017; Huang et al., 2022). The qualified DNA was submitted to Majorbio Biopharm Technology Co., Ltd (Shanghai, China) for Illumina MiSeq amplicon sequencing (2 × 300 bp). Primers 338F and 860R were used to amplify the V3-V4 region of bacterial 16S rRNA gene. For fungi, the internal transcribed spacer (ITS) region was amplified with primers ITS1F and ITS2 (Nie et al., 2013; Huang et al., 2022). After sequencing, the raw sequences were quality-filtered by removing sequences as previously described (Zhang et al., 2020). Qualified reads from all samples were removed chimera sequences and assigned to operational taxonomic units (OTUs) at a 97% sequence similarity threshold with QIIME (version 1.9.1) pipeline (Caporaso et al., 2010). The representative OTU sequences were aligned against the bacterial 16S rRNA gene database Greengenes (version 13.8) (DeSantis et al., 2006) and the fungal ITS database UNITE (version 7.1) (Abarenkov et al., 2010) for taxonomic classification. The analysis of alpha-diversity and betadiversity were conducted after rarefying all samples to the same sequencing depth (Huang et al., 2022).

### 2.5. Data analysis

#### 2.5.1. Statistical analysis

SPSS statistical software was used to analyze and report the results as mean standard deviation. The significance level for the independent *t*-test used to compare the differences between the two inoculation strategies was set at 0.05. One-way analysis of variance and Tukey HSD inspection were performed at a significance level of 0.05 to determine whether the two inoculation strategies had a significant effect on quality.

#### 2.5.2. High-throughput sequencing data analysis

The onboard data of PacBio SMRT were exported, the CCS was identified through the barcode using Lima V1.7.0 software, and the barcode-CCS sequence data were obtained. Subsequently, barcode-CCS was filtered to obtain an effective sequence. Finally, the chimeric sequences were identified and eliminated using UCHIME V4.2 software to obtain the optimized ccs sequences. The valid sequences of the samples were clustered using USEARCH software, and the sequences were clustered into the operational taxonomical unit (OTU) under the condition of 97% similarity. Moreover, the most abundant sequence (MOST ABUNDANT) was selected as the OTU representative sequence. Using SILVA 132 as the reference database, the naive Bayesian classifier was combined with the alignment method for taxonomic annotation of the sequences, with a 70% confidence threshold. Sample α diversity index was evaluated using the Mothur V1.30 software. Furthermore, β diversity was analyzed with QIIME software, and the similarity of species diversity among various samples was compared.

#### 2.5.3. Correlation analysis

Spearman correlation analysis was performed on the relative abundance of >1% and the difference or key strain with the physicochemical factors and main volatile flavor substances in vinegar-fermented grains, respectively, to calculate the correlation coefficient R-value and significant difference *p* value, which were analyzed and plotted using GraphPad Prism 7.0 and R language and other software.

## 3. Results and discussion

### 3.1. Differences in physicochemical properties of different inoculation strategies during fermentation

**Figure 1:**
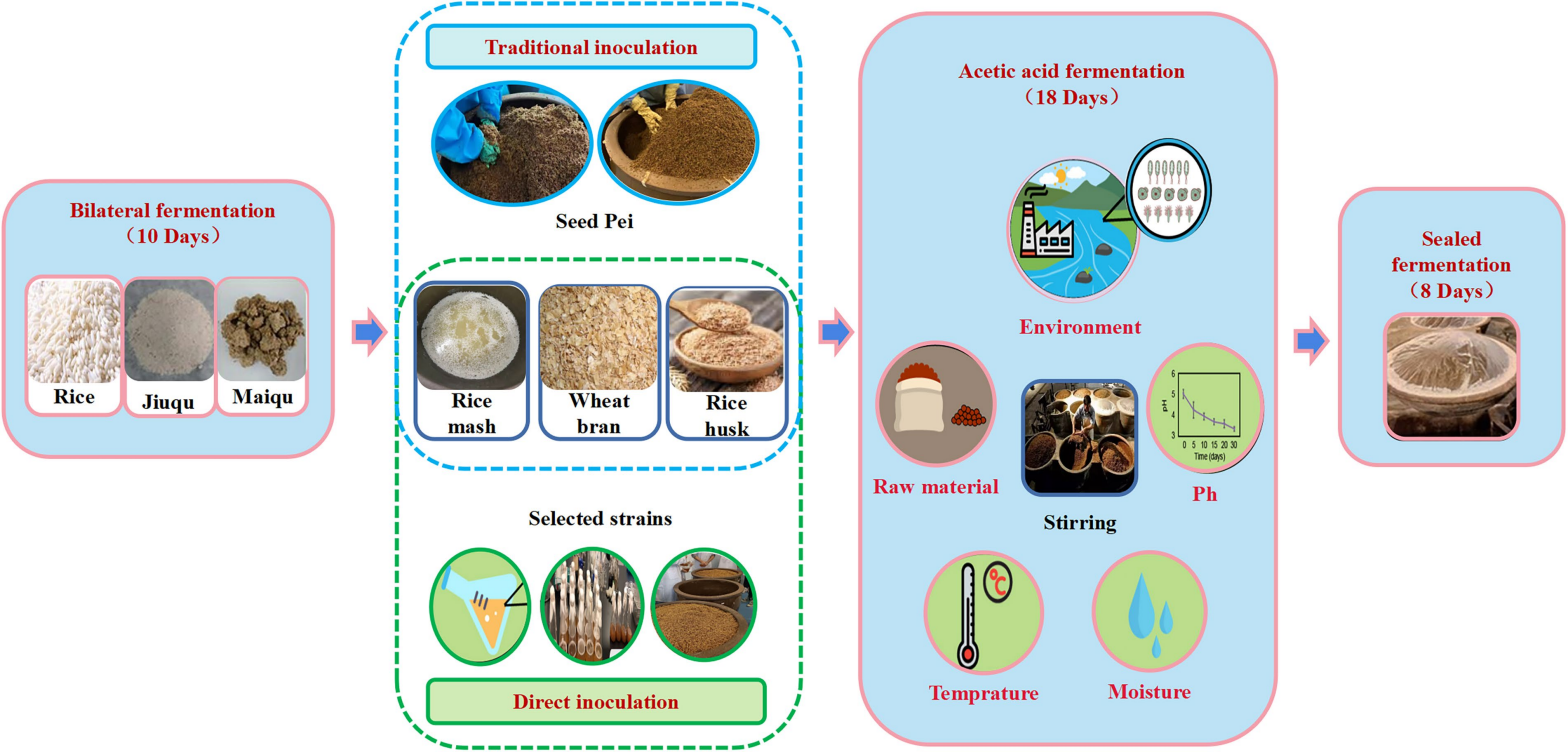
Fermentation process of Zhenjiang aromatic vinegar under the traditional inoculation strategy and the direct-injection inoculation strategy.

To study the dynamic changes in physicochemical parameters and flavoring substances during acetic acid fermentation of Zhenjiang aromatic vinegar under different inoculation strategies, the total acid (Figure 1A), reducing sugar content (Figure 1B), nonvolatile acid content (Figure 1C), fermented grain temperature (Figure 1D), organic acid content (Figures 1E,F), and free amino acid content (Figures 1G,H) were measured during the fermentation process. These parameters were important physicochemical indices related to microbial growth during fermentation (Lu et al., 2018).

The total acid content of vinegar-fermented grains exhibited a fluctuating upward trend throughout the acetic acid fermentation process, which was a key indicator of vinegar acidity. On days 0–7 of acetic acid fermentation, the total acid content continued to increase owing to the mass propagation of microorganisms such as lactic acid bacteria. Aerobic microorganisms such as acetic acid bacteria proliferate in a large number and generate a large amount of organic acids including acetic acid and lactic acid for 7 to 18 days after the fermented grains are completely turned over for fermentation in order that the total acid content increases rapidly (Wang et al., 2015). At the end of fermentation, the total acid content using the traditional inoculation strategy was as high as 6.21 ± 0.02 g/100 g, the highest dose achieved *via* direct inoculation was 6.91 g/100 g.

The sweet flavor of Zhenjiang aromatic vinegar is primarily a result of the saccharification of food. These saccharides can enhance the flavor of vinegar. Simultaneously, the reducing sugar of vinegar can react with amino acids to produce flavor substances (Fang et al., 2021). On the whole, the change trend of reducing sugar content under the direct inoculation strategy and the traditional inoculation strategy is roughly the same, both of which show a trend of increasing first and then decreasing (Figure 1B). The main difference between the two inoculation strategies in the acetic acid fermentation process is that the traditional inoculation strategy is in the rising period of reducing sugar content in 4 ~ 8 days, reaching the maximum value of 2.27 ± 0.03 g/100 g on the 8th day of fermentation, and gradually decreasing in the later stage. The direct inoculation strategy is that the content of reducing sugar is in the rising stage on 6 ~ 14 days and reaches the maximum value of 3.27 ± 0.01 g/100 g on the 14th day of fermentation. There may be two reasons for the increase of reducing sugar content in the early stage. First, reducing sugar mainly comes from bran during acetic acid fermentation. The content of reducing sugar in wine mash and rice husk is relatively low. In the early stage, as the acidity of vinegar *pei*, the macromolecular sugar contained in raw material bran is hydrolyzed into small molecular sugar by acid, such as monosaccharide and other reducing sugar. At the same time, the temperature reached the optimum temperature of saccharifying enzyme (35°C-40°C), the activity of the enzyme increased, and the reducing sugar produced by hydrolysis of raw materials was accumulated. Because the activity and acid-producing ability of acetic acid bacteria under the direct inoculation strategy is stronger than that of the traditional inoculation strategy, it can fully acidolysis macromolecular sugar and produce more reducing sugar. In the late stage of acetic acid fermentation, microorganisms proliferate, nutrients are rapidly consumed, and reducing sugar content is significantly reduced.

The non-volatile acids can neutralize the strong acidity of acetic acid and make the finished vinegar taste soft and delicate, which is the key to the quality of Zhenjiang balsamic vinegar (Wang et al., 2015). Lactic acid is the most important nonvolatile acid (Wang et al., 2015). Under the traditional inoculation strategy, the nonvolatile acid content was maximum in the fermentation process after 12 days, reaching up to 0.41 ± 0.01 g/100 mL, whereas it reached up to 1.58 g/100 mL under direct inoculation.

Temperature affects the metabolic activity of microorganisms and the changes of alcohol and water during the fermentation process, making it a comprehensive indicator used to determine the fermentation quality of vinegar-fermented grains. Under optimal conditions, the rapid propagation and metabolism of microorganisms not only rapidly converted the alcohol in vinegar-fermented grains into acetic acid but also metabolized a large number of organic acids, amino acids, esters and other substances. The fermentation temperature ranged from 38°C to 46°C, which was the most active phase of acetic acid bacterial metabolism. A large number of products were accumulated, and the primary flavoring substances of aromatic vinegar were generated, alongside a large amount of heat. During the final phase of fermentation, owing to the consumption of nutrients and large accumulation of metabolites, the metabolism of microorganisms such as acetic acid bacteria was inhibited, and the temperature was reduced and maintained at 35°C (Shi et al., 2022). Using the traditional inoculation strategy, the fermented grain temperature of vinegar reached a maximum of 46.07°C ± 1.10°C on the eighth day of fermentation and then decreased to 27.33°C ± 2.48°C. however, the temperature of vinegar *Pei* under the direct inoculation strategy continued to rise until the fermentation temperature reached 48.0°C on the twelfth day and then began to decrease to 35.33°C ± 0.35°C. It was possible that the direct inoculation of acetic acid bacteria contained a high concentration of lactic acid bacteria and produced more acid and heat, which inhibited the growth of other bacteria.

**Figure 2:**
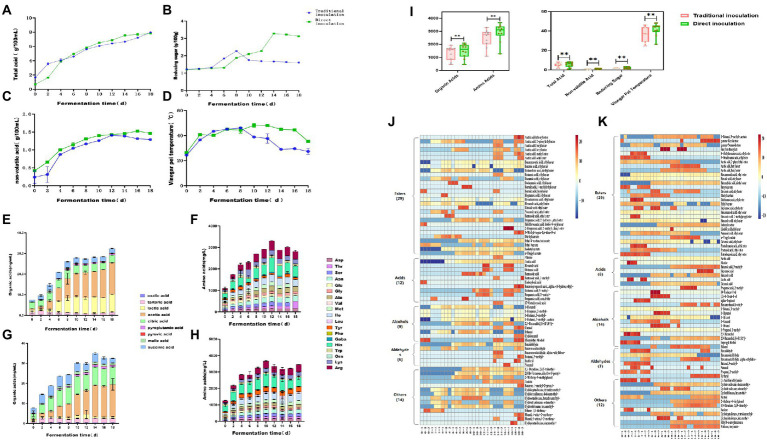
Dynamic changes in physicochemical parameters and flavoring substances during the fermentation of Zhenjiang aromatic vinegar using different inoculation strategies. **(A)** Total acid, **(B)** reducing sugar content, **(C)** nonvolatile acid content, **(D)** fermented grain temperature, **(E)** organic acid content using the traditional inoculation strategy, **(F)** organic acid content using the direct inoculation strategy, **(G)** free amino acid content using the traditional inoculation strategy, **(H)** free amino acid content using the direct inoculation strategy, **(I)** total acid, nonvolatile acid, reducing sugar, vinegar-fermented grain temperature, and total content of organic acid and organic acid in the fermentation process of Zhenjiang aromatic vinegar using different inoculation strategies, **(J)** volatile flavoring substance content using the traditional inoculation strategy, and (K) volatile flavoring substance content using the direct inoculation strategy.

Overall, there were considerable differences in total acid, nonvolatile acid, reducing sugar, the temperature of fermented vinegar, organic acid, and organic acid content between the two inoculation strategies (Figure 2). Moreover, the total acid of fermented vinegar exhibited an upward trend. The total acidity of fermented vinegar inoculated using the direct inoculation strategy was considerably greater than that of vinegar inoculated using the traditional strategy. In the first 12 days, the total acid of acetic acid fermentation using the traditional inoculation strategy increased faster than that using the direct inoculation strategy, but then the total acid of acetic acid fermentation using the direct inoculation strategy surpassed that using the traditional inoculation strategy and reached 6.91 g/100 g dry fermented grains. The reducing sugar content in the acetic acid fermentation process using the two inoculation strategies increased continuously for 8 days before fermentation, but after 8 days of fermentation, the reducing sugar content in the vinegar-fermented grains using the traditional inoculation strategy gradually decreased, whereas that in the vinegar-fermented grains using the direct inoculation strategy continued to increase. The concentration of nonvolatile acid first increased and then decreased. The amount of nonvolatile acid in fermented grains fermented using the direct inoculation strategy was considerably greater than in those fermented using the traditional inoculation strategy. The temperature of vinegar-fermented grains exhibited a fluctuating upward trend 8 days before fermentation, and the fermentation temperature of vinegar-fermented grains using the direct inoculation strategy was higher than that using the traditional inoculation strategy. These physical and chemical parameters are not only the indices of normal fermentation, but also the potential environmental drivers of microbial community accumulation in fermented foods (Zheng et al., 2014; Xiao et al., 2017).

According to the results, the total amino acid content in the acetic acid fermentation stage tended to increase first and then decrease (Figures 1G,H). The total amino acid content in acetic acid fermentation (AAF) reached a maximum of 3301.46 ± 13.41 mg/100 g dry fermented grains on the 12th day using the traditional inoculation strategy, whereas the total amino acid content of the direct inoculation strategy reached the highest of 3666.18 ± 14.40 mg/100 g dry fermented grain on the 10th day (Figures 1G,H). The traditional inoculation strategy produced more His, Arg, Ile, and Lys, whereas the direct inoculation strategy produced more His, Arg, Ile, and Tyr. Organic acid is the primary source of the sour taste of vinegar and the most important flavoring substance of vinegar (Zhu et al., 2016). Vinegar primarily contained volatile acids such as formic acid, acetic acid, propionic acid, and butyric acid, and nonvolatile acids such as lactic acid, tartaric acid, citric acid, malic acid, and succinic acid (Nie et al., 2017). Using the traditional inoculation strategy, the total organic acid content increased continuously *via* fermentation and reached 1939.66 ± 4.16 mg/100 g dry fermented grains at the end of AAF, among which acetic acid, lactic acid, and citric acid were the highest produced. Using the direct inoculation strategy, the organic acid content increased to 2099.63 ± 4.13 mg/100 g dry fermented grains on the 14th day of AAF and then decreased slightly, with acetic acid, citric acid, and tartaric acid accounting for most (Figures 1E,F). Acetic and lactic acids is the primary organic acids in grain vinegar, with acetic acid being the volatile acid with the highest concentration and possessing the characteristics of strong irritability and short aftertaste. Lactic acid, citric acid, tartaric acid, and other nonvolatile acids can adjust the sour taste, endowing vinegar with a soft flavor, which is conducive to the equilibrium of acetic acid sour taste stimulation (Lv et al., 2016). Lactobacillus and Acetobacter are the high-abundance microorganisms in the AAF process of traditional grain vinegar (Ai et al., 2019; Kou et al., 2022). Due to the participation of these microorganisms, the metabolic flavor substances produced by fermentation are more abundant (Wu et al., 2017). Overall, the content of organic acid, amino acid and total acid in fermented grains under the direct inoculation strategy was slightly higher than that under the traditional inoculation strategy.

Compared with organic and amino acids, the volatile substances in fermented vinegar were considerably low in concentration, but they played a substantially important role in vinegar, as the majority of them possessed unique aromas that contributed to a more balanced and diversified flavor. According to previous research reports, the characteristic flavoring substances of Zhenjiang aromatic vinegar mainly include acetic acid, lactic acid, phenethyl alcohol, 3-methylbutyraldehyde phenylacetic acid, 2,3-butanedione, 3-hydroxy-2-butanone, ethyl acetate, and acetoin (Yu et al., 2012). In conclusion, 70 volatile compounds were identified in AAF vinegar-fermented grains using traditional and direct inoculation strategies (Figures 1I,J). Twenty-nine distinct esters of volatile substances were identified using both the inoculation strategies. The concentrations of acids (12 types) and other volatile substances (14 types) such as ketones and phenols using the traditional inoculation strategy were higher than those of acids (8 kinds) and other flavor compounds (12 kinds) using the direct inoculation strategy. However, the numbers of alcohols (14 kinds) and aldehydes (7 types) using the direct inoculation method were higher than those of alcohols (9 types) and aldehydes (6 types) using the traditional inoculation strategy. Furthermore, 13 identical esters, 5 identical acids, 6 identical alcohols, 6 identical aldehydes, and 5 identical ketones, phenols, and other volatile substances are shown in Table 1 for both the inoculation strategies.

**Table 1:** Same flavoring substances and special flavoring substances under different inoculation strategies.

Kind	Common flavoring substances	Flavoring substances peculiar to traditional inoculation strategies	Flavoring substances peculiar to direct inoculation strategy
Esters	Acetic acid ethenyl ester, Acetic acid, hexyl ester, benzeneacetic acid, ethyl ester, benzoic acid, ethyl ester, Decanoic acid, ethyl ester, dodecanoic acid, ethyl ester, heptanoic acid, ethyl ester, hexanoic acid, ethyl ester, linoleic acid ethyl ester, nonanoic acid, ethester, pentanoic acid, ethyl ester, ethyl acetate, Isobutyl acetate and n-Propyl acetate	Acetic acid, 2-phenylethyl ester	1-Butanol, 3-methyl-, acetate
		Acetic acid, butyl ester	Gamma-decalactone
		Acetic acid, methyl ester	Gamma-nonanolactone
		Acetic acid, octyl ester	Hexyl methacrylate
		Butanedioic acid, diethyl ester	8-Methylnonanoic acid, ethyl ester
		Butanoic acid, ethyl ester	9-Octadecenoic acid, ethyl ester
		Formic acid, 1-methylethyl ester	Acetic acid, 2-phenylethyl ester
		Formic acid, hexyl ester	Butanedioic acid, diethyl ester
		Hexadecanoic acid, ethyl ester	Butyrolactone
		Propanoic acid, 2-hydroxy-, ethyl ester	Diethyl azelate
		Trichloroacetic acid, dodec-9-ynyl ester	Hexadecanoic acid, ethyl ester
		2-Propenoic acid, 2-methyl-, hexyl ester	Hexanoic acid, 2-methylpropyl ester
		3-Methyl-hepta-1,6-dien-3-ol	Octanoic acid, ethyl ester
		Diethyl azelate	Pentadecanoic acid, ethyl ester
		Ethyl 4-acetoxybutanoate	Tetradecanoic acid, ethyl ester
		Alanine	
Acids	Acetic acid	Pentanoic acid	Alanine
	Hexanoic acid	Pentanoic acid, 3-methyl	Heptanoic acid
	Octanoic acid	Undecylenic acid	Lactic acid
	Butanoic acid, 3-methyl-	Benzenepropanoic acid, .alpha.-(1-hydroxyethyl)	
	Propanoic acid, 2-methyl-	Propanoic acid, anhydride	
		17-Octadecynoic acid	
Alcohol	1-Hexanol	1-Butanol, 3-methyl-, acetate	10-Undecen-1-ol
	1-Propanol, 2 – methyl-	Creosol	(Z)-4-Decen-1-ol
	1 − Butanol, 3 – methyl-	Cyclobutanol	alpha−Terpineol
	2,3-Butanediol, [S−(R*,R*)]	Phenylethyl alcohol	Benzeneethanol, b-ethyl-
	Ethanol		1-Heptanol
	Acetoin		1-Nonanol
			1-Octanol
			2,3-Butanediol
			Isopropyl Alcohol
Aldehydes	Benzaldehyde	–	Propanal, 2-methyl-
	Benzeneacetaldehyde		
	Benzeneacetaldehyde, .alpha.-ethylidene-		
	Butanal, 3-methyl-		
	Furfural		
	Nonanal		
Other	1,3 − Dioxolane, 2,4,5-trimethyl-	2(3H)-Furanone, dihydro-5 – pentyl-	(2-Aziridinylethyl)amine
	Cycloheptasiloxane, tetradecamethyl-	2-Methoxy-5-methylphenol-	2-Methoxy-4-vinylphenol
	Cyclohexasiloxane, dodecamethyl-	Benzene, 1-methyl-2-propyl-	Azulene
	Cyclotetrasiloxane, octamethyl-	Cyclooctasiloxane, hexadecamethyl-	Cyclopentasiloxane, decamethyl-
	Cyclotrisiloxane, hexamethyl-	Ethane, 1,1-diethoxy-	Ethyl 4-acetoxybutanoate
		Phenol, 4-ethyl-2-methoxy-	Methane, isocyanato-
		Phenol, 5-ethenyl-2-methoxy-	
		Cyclopentasiloxane, decamethyl-	

With the development of fermentation, most volatile substances in fermented grains showed an increasing trend. Acetoin was produced in the acetic acid fermentation process during both inoculation strategies. Acetoin is the precursor of ligustrazine (2,4,5,6-tetramethylpyrazine (TTMP), nut and baking aroma). According to reports, TTMP is the primary bioactive component in vinegar (Wang et al., 2016). As fermentation progressed, the concentration of acetoin steadily increased. On the 18th day of fermentation, the amount of acetoin directly inoculated with *Pei* was 5.88 ± 2.81 mg/100 g dry *Pei*, whereas the amount of acetoin using the traditional inoculation strategy was only 3.35 ± 0.42 mg/100 g dry *Pei*. Therefore, this result indicated that the direct inoculation strategy could effectively promote the production of acetoin.

Esters are the most abundant volatile flavoring substances, and ethyl acetate and ethyl lactate had the highest concentrations in the entire process, which was directly correlated with the higher concentrations of the synthetic precursors of acetic acid and lactic acid (Wang et al., 2016). The environment of solid vinegar fermented grains is extremely conducive to the proliferation and fermentation of ester-producing yeast, making a large number of ester substances produced and accumulated in acetic acid fermentation. In addition to the common ester flavor substances, there are 16 kinds of esters unique to the traditional vaccination strategy, such as 2-phenylethyl acetate, butyl ester and methyl ester of acetic acid and valeric acid. There are 15 direct injection vaccination strategies, such as acetate, gamma decalac, and gamma nonlac. The production of alcohols in the fermentation process, such as hexanol, 3-octanol (nuts, mushrooms) and phenylethanol (flowers, honey), brings richer flavor. Acetic acid, methylbutyric acid, caproic acid, ethanol and phenylethanol are the main acids and alcohols in the production of Zhenjiang vinegar. There are four kinds of alcohols unique to traditional vaccination strategies, including 1-butanol, 3-methyl-acetate and creosote. The direct injection vaccination strategy includes nine kinds, such as 10-undecen-1-ol, (Z) - 4-deca-1-ol, etc. There are 7 kinds of acid specific to traditional vaccination strategy, including 3-methylundecanoic acid. The direct injection vaccination strategy includes three kinds, such as alanine and heptane.

### 3.2. Analysis of differences in the structural composition of microbial communities using different inoculation strategies during fermentation

**Figure 3:**
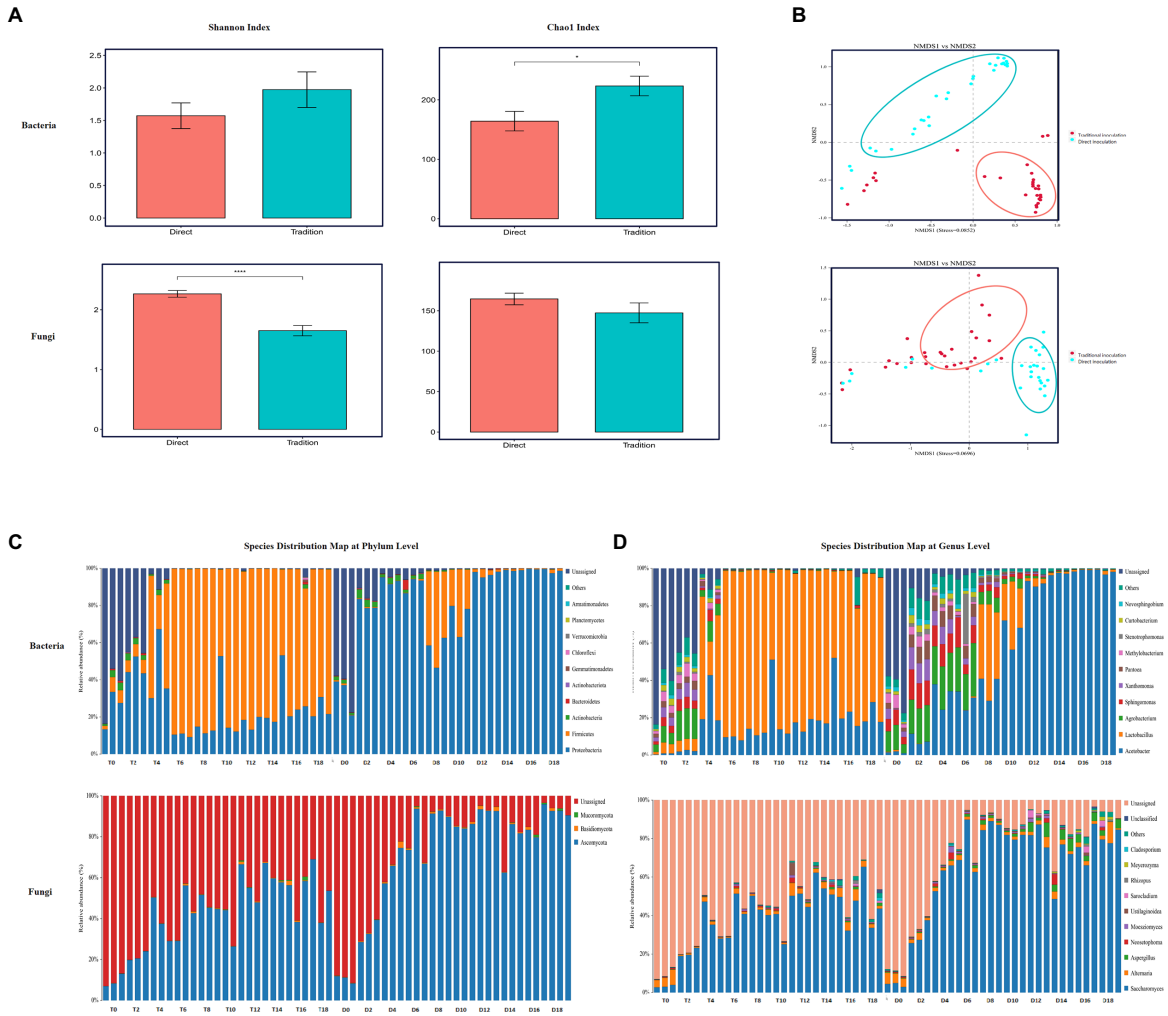
Analysis of microbial community diversity during the fermentation of Zhenjiang aromatic vinegar for different inoculation strategies. **(A)** α diversity of acetic acid fermentation microbial community for different inoculation strategies, including shannon index and Chao1 index. **(B)** NMDS map of microorganisms for different inoculation strategies. **(C)** Diversity analysis of microbial community during the fermentation of Zhenjiang aromatic vinegar for different inoculation strategies at the gate level. **(D)** Diversity analysis of microbial community during the fermentation of Zhenjiang aromatic vinegar for different inoculation strategies at the genus level.

The structure and succession pattern of bacterial and fungal communities throughout the entire fermentation process for the two inoculation strategies was investigated by using amplicon sequencing analysis. Significant differences were found between the Chao1 and Shannon indices of acetic acid fermentation bacteria and fungi for the direct inoculation strategy and those for the traditional inoculation strategy (Figure 3A). The Chao1 and Shannon indices of bacterial and fungal communities were greater for the traditional inoculation strategy than for the direct inoculation strategy. Inoculated acetic acid bacteria and lactic acid bacteria produced a large amount of acid, which affected the internal environment of fermented grains and inhibited the growth and reproduction of other microorganisms. Moreover, the vinegar-fermented grain communities for the traditional inoculation strategy (blue) and the direct inoculation strategy (red) in acetic acid fermentation gathered into a cluster, and the distribution distance between the communities was wide (Figure 3A), indicating that the inoculation strategy considerably affected the community structure of bacteria and fungi.

Throughout the entire process of acetic acid fermentation, bacteria predominate the metabolic activities in vinegar culture, and their biomass in vinegar-fermented grains is higher than that of fungi (Wang et al., 2016; Chai et al., 2020). Fungi are rare and diverse species in vinegar culture. Their presence provides a biological buffer against environmental changes, but their intrinsic growth rate is low (Huang et al., 2022). The species distribution maps at the phylum (Figure 3C) and genus levels (Figure 3D) revealed that the microflora in vinegar-fermented grains under the two inoculation strategies consisted primarily of bacteria such as Bacteroides, Actinomyces, Sclerotinia, and Proteus as well as fungi such as Myxomycetes, Basidiomycetes, and Ascomycetes, with lactic acid bacteria and Acetobacter as the predominant bacteria in general (Figure 3C). Yeasts and Alternaria were the predominant fungi.

During AAF, the relative abundance of Lactobacillus increased initially and then decreased, whereas the relative abundance of *Acetobacter* continue to increase. During this stage, the predominant microorganisms were lactic acid bacteria and *Acetobacter*, including *Komagataeibacter* and *Gluconacetobacter*. In addition to oxidizing alcohol substrates such as ethanol and sugar alcohols *via* the respiratory chain to organic acids such as acetic acid and lactic acid, *Acetobacter* could promote the production of certain ketone and aldehyde flavoring substances (Huang et al., 2022). Lactic acid bacteria play an important role in the formation of vinegar taste by producing organic acids such as acetic acid, malic acid, and lactic acid, and by synthesizing 2,3-butanediol and acetoin as precursors of tetramethylpyrazine (Chai et al., 2020). Additionally, during the fermentation process, bacillus generates organic acid *via* a tricarboxylic acid circulation pathway to improve the pungent sour taste caused by acetic acid and soften the mouth. Simultaneously, the flavor of vinegar is improved by the secretion of protease to decompose protein and generate amino acids (Nie et al., 2013). Owing to open fermentation, some bacteria in the environment participate in the esterification reaction, producing esters (Xu et al., 2011). As the acetic acid fermentation process progressed, the dominant microbial population for fermentation also changed (Figure 3D). During AAF, the relative abundance of acetic acid bacteria under the direct inoculation strategy was significantly higher than that under the traditional inoculation method, while lactic acid bacteria was on the contrary, because the direct inoculation strategy used biologically enhanced acetic acid bacteria as a bacterial agent to inoculate in the fermented grains, and acetic acid bacteria inhibited the activity of lactic acid bacteria. However, *Agrobacterium*, *Sphingomonas* and other major bacterial genera showed a trend of increasing first and then decreasing in the fermentation process, and the relative abundance of bacteria under the direct inoculation strategy was slightly higher than that under the traditional inoculation strategy.

The proportion of fungi in the two inoculation strategies was considerably lower than that of bacteria. Fungi are well known to play an important role in fermenting foods (Venturini, 2019). However, there are few studies on the changes in fungal communities during vinegar fermentation. Studies have proven that fungi are important contributors to flavor production in AAF and are rare communities (Huang et al., 2022). During AAF, *Saccharomyces*, *Alternaria* and *Aspergillus* were the dominant fungi under the two inoculation strategies. Like the above bacteria, the relative abundance of bacteria under the direct inoculation strategy is slightly higher than that under the traditional inoculation strategy. The unidentified microorganisms in vinegar need further study. In addition to produce beneficial nutrients and flavor substances, some microorganisms may also produce harmful substances and their precursors, such as biogenic amines, urea, carbamate and other substances (Gao et al., 2023). Studies have shown that rare microflora may considerably affect the local microbial interactions during environmental disturbance (Jiang et al., 2019). The future research should focus more on the role of fungi in the structure of the fermentation microbial community and the generation of vinegar flavor (Huang et al., 2022).

### 3.3. Correlation analysis between internal environment of fermented grains and main microorganisms of different inoculation strategies during fermentation

In the AAF process, the formation of solid vinegar is the result of the continuous reproduction and metabolism of flavor substances by a large number of microbial communities using fermentation raw materials. The environment has a substantial effect on the metabolism of microbial communities. Once fermentation begins, the rapidly changing environmental conditions of the niche (e.g., the high ethanol content and anaerobic environment of fermented rice milk, and the high acidity of vinegar fermentation) provide selective strength for the ‘systematic self-domestication’ of microbial communities, thus shaping the decision-making process of microbial succession (Lu et al., 2018).

**Figure 4:**
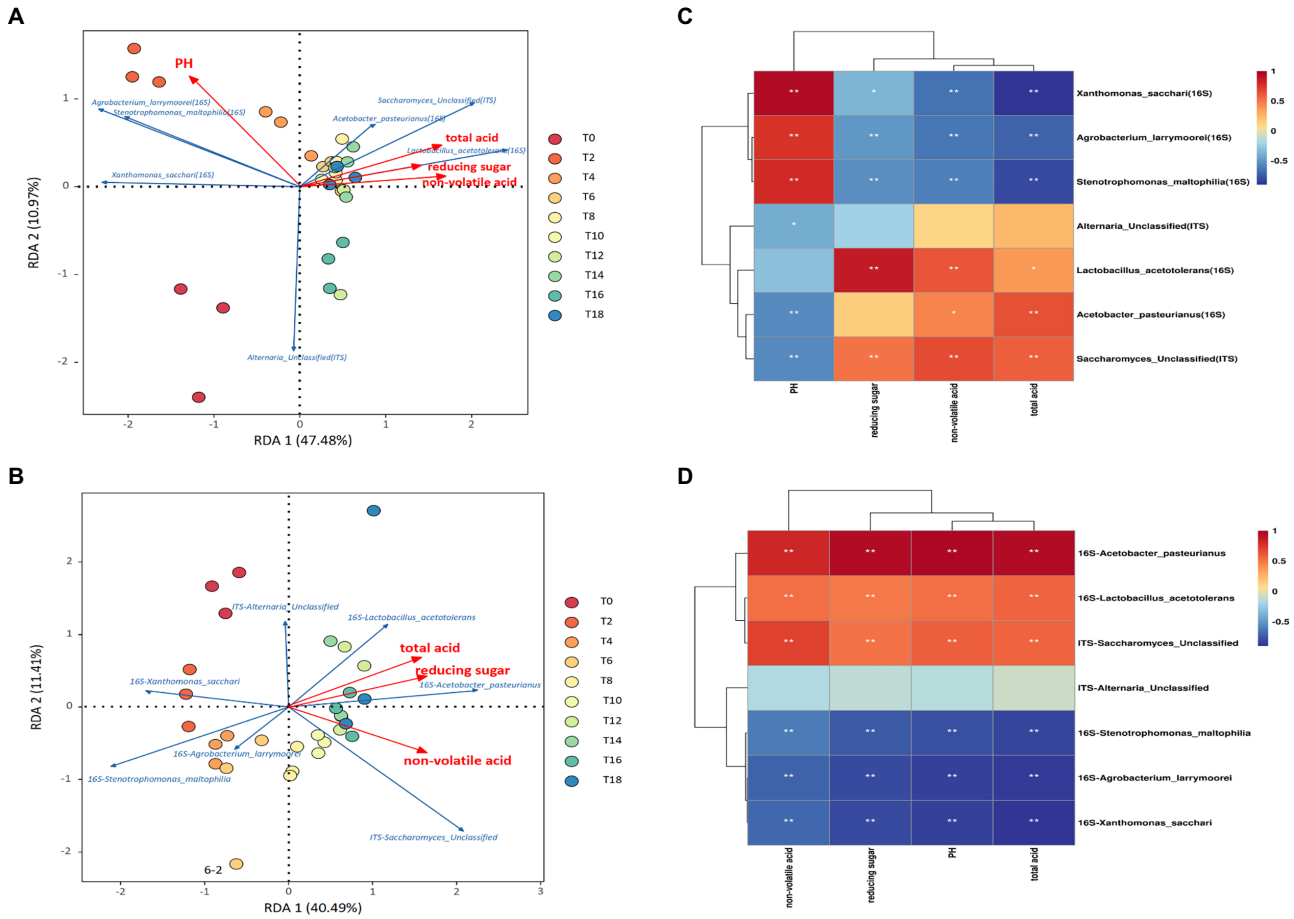
Correlation analysis between main bacteria and fungi for different inoculation strategies and environmental parameters during fermentation. **(A)** RDA diagram of main microorganisms and environmental parameters for the traditional inoculation strategy. **(B)** RDA of main microorganisms and environmental parameters for the direct-injection inoculation strategy. **(C)** Correlation heat diagram of main microorganisms and environmental parameters for the traditional inoculation strategy. **(D)** Correlation heat diagram of main microorganisms and environmental parameters for the direct inoculation strategy.

Environmental factors had a significant impact on the fermentation of vinegar in the solid state. In addition to the indoor temperature, humidity, and other external environments of fermented grains, the total acid, nonvolatile acid, and reducing sugar of vinegar-fermented grains were not only metabolites of acetic acid fermentation but also substrates of vinegar production, making them crucial environmental variables in vinegar-fermented grains. Redundancy analysis (RDA) and Spearman were used to conduct correlation analysis on seven bacterial and seven fungal groups (with average relative abundance >10%) and four environmental parameters to determine the relationship between the succession of microbial communities during the acetic acid fermentation stage and environmental changes caused by different inoculation strategies (Figure 4).

According to the RDA results, a significant correlation existed between prevalent microorganisms and environmental parameters (Figures 4A,B). Various environmental factors influence the survival and reproduction of various microorganisms. The cumulative interpretation of the relationship between major microorganisms and environmental factors on Axis I and Axis II was as high as 47.48 and 40.49%, respectively, in vinegar-fermented grains after traditional and direct inoculation (Figures 4A,B). In conjunction with the Spearman correlation coefficient and Figures 4C,D, the heat map of correlation between the main bacteria and fungi (average relative abundance >10%) and the environmental parameters in the fermentation process, for the two inoculation strategies, *Acetobacter pasteurianus*, *Lactobacillus acetotolerans*, and *Saccharomyces* sp (unclassified) were significantly positively correlated with reducing sugar, nonvolatile acid, and total acid. *Acetobacter pasteurinus* has strong ethanol resistance and can convert ethanol in fermented grains into lactic acid, acetic acid and other substances (Jiang et al., 2019). *Lactobacillus acetotolerans* prefers high-acid environment (growth pH 3.0–5.0, optimum pH 4.0), and its relative abundance will increase with acetic acid fermentation (Yu et al., 2020). *Saccharomyces sp.*, the main fungus in vinegar fermented grains, was brought into the fermented grains during the alcohol fermentation stage. *Saccharomyces sp*. decomposes polysaccharides to produce more reducing sugars, and decomposes monosaccharides to produce alcohol. *Saccharification* is accompanied by alcoholization and acetic acid. The content of non-volatile acid and volatile acid in vinegar fermented grains increases rapidly (Zhang et al., 2017). *Xanthomonas* is a common bacteria brought in from the fermented mash (Zhang et al., 2017). With the continuous reduction of acetic acid fermentation abundance, *Xanthomonas* is a small number of bacteria that can finally adapt to the acidic environment and remain in the microbial community of fermented mash with relatively low abundance (Wang et al., 2016). *Alternaria* is the dominant fungus genus of Zhenjiang vinegar (Wang et al., 2016). These bacteria were negatively correlated with reducing sugar, non-volatile acid and total acid. Under the traditional inoculation strategy, *Acetobacter Pasteuri*, *Acetyl-tolerant Lactobacillus* and *Saccharomyces cerevisiae (unclassified)* were significantly negatively correlated with pH. *Xanthomonas*, *Lactobacillus* and *Alternaria (unclassified)* were significantly positively correlated with the pH value of the direct inoculation strategy. Therefore, under different inoculation conditions, pH has the most significant effect on microorganisms. The pH value will affect the activity of microorganisms such as *Acetobacter* and *Lactobacillus* during acetic acid fermentation. When the pH value in the fermentation broth is too high or too low, the growth of microorganisms will be inhibited. In addition, pH value can also affect the accumulation of nutrients in the fermentation broth and the stability of the metabolic substances of acetic acid bacteria, thus reducing the acid production capacity of acetic acid bacteria (Wang et al., 2015).

### 3.4. Correlation analysis between flavoring substances and main microorganisms for various inoculation strategies during fermentation process

**Figure 5:**
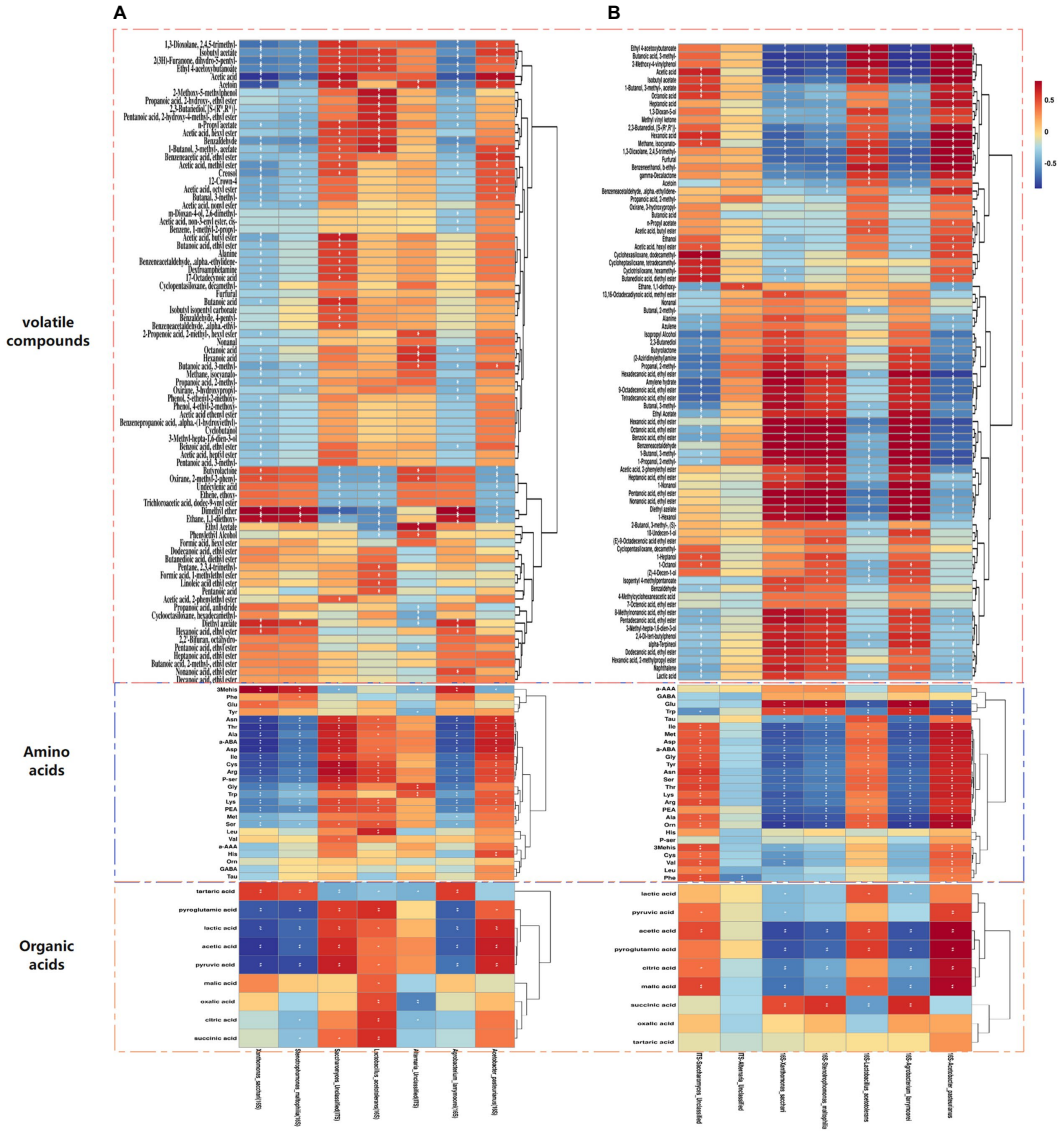
Correlation analysis between main bacteria and fungi and metabolites (volatile compounds, amino acids, and organic acids) during fermentation for different inoculation strategies. **(A)** A correlation heat map of microorganisms and flavor substances produced in the acetic acid fermentation process for the traditional inoculation strategy. **(B)** A correlation heat map of microorganisms and flavoring substances produced in the acetic acid fermentation process for the direct inoculation strategy.

**Figure 6:**
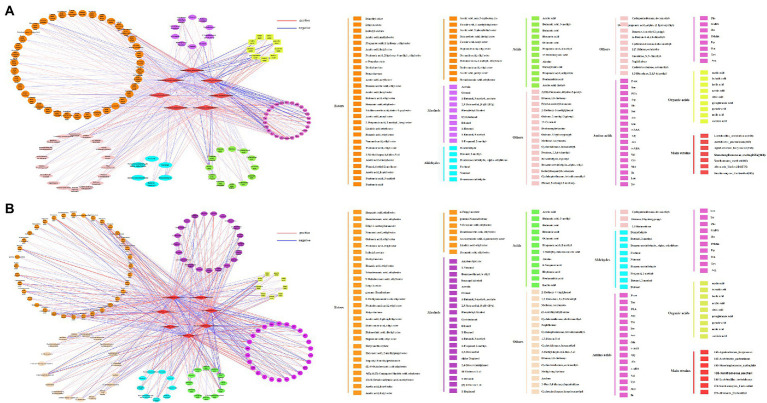
**(A)** A related network diagram of microorganisms and flavoring substances produced in the acetic acid fermentation process for the traditional inoculation strategy. **(B)** The relevant network diagram of microorganisms and flavoring substances produced in the acetic acid fermentation process for the direct inoculation strategy.

To fully understand the correlation between flavoring substances and the predominant microorganisms for different inoculation strategies, the Spearman correlation coefficient was used to investigate the correlation between important microorganisms (average relative abundance >1%) and flavoring substances formed during the fermentation process, The red line represents positive correlation, and the blue line represents negative correlation (Figures 5A,B, 6A,B). *Acetobacter Pasteurianus*, the main bacteria under the traditional inoculation strategy, is positively correlated with active acid, acid acid, pyruvic acid, lAsn, Thr, Ala, a-ABA, Asp., Ile, Cys, Arg, P-ser, His and Acetin. *Lactobacillus Acetotolerans,* the main bacteria under the traditional inoculation strategy, is positively correlated with oxalic acid, citric acid, pyroglutamic acid, successive acid, Leu, Cys, Arg, P-ser, Lys, PEA, 2-Methoxy-5-methylphenol, propanoic acid, 1-butanol, 3-methyl-, acetate (Figures 5A, 6A). While, in the direct inoculation strategy, the main bacteria, *Acetobacter Pasteurianus,* is positively correlated with acid acid, pyroglutamic acid, citric acid, malic acid, ethyl 4-acetoxybutyloate, butanoic acid, 3-methyl-, 2-Methoxy-4-vinylphenol, acid, isobutyl acetate, hexanoic acid, methane, isocyanato-, 1,3-Dioxolane, 2,4,5-trimethyl-, benzeneethanol, b-Ethyl-, and negatively correlated with hexadecanoic acid, ethyl ester. Meanwhile, *Acetobacter Pasteurianus* is positively correlated with Ile, Met, Asp., a-ABA, Gly, Tyr, Asn, Ser, Thr, Lys, Arg, PEA, Ala, Orn. *Lactobacillus Acetotolerans* under the direct inoculation strategy is positively correlated with ethyl 4 − acetoxybutanoate, butanoic acid, 3 − methyl−, 2 − methoxy−4 − vinylpheno, acetic acid, benzeneethanol, b − ethyl−, and negatively correlated with acid and pyroglutamic acid (Figures 5B, 6B). This shows that the function of acetic acid bacteria under the direct inoculation strategy is strong, which is more conducive to the generation of organic acids, non-volatile acids and volatile flavor compounds than the traditional inoculation strategy. Jia et al. (2020) cultivated *Acetobacter Pasteuri* and *Lactobacillus plantarum*, which are conducive to the formation of flavor substances in Shanxi aged vinegar, so as to improve the content of flavor substances that contribute to the flavor of Shanxi aged vinegar, such as benzyl alcohol, ethyl acetate, phenylethyl acetate, 2,5-dimethylpyrazine, which are aroma-active compounds with floral, fruity, sweety and chocolate-like notes (Gao, 2021; Zhang et al., 2022).

In addition to the most important *Acetobacter and Lactobacillus,* other major microorganisms, such as *Agrobacterium Larrymorei* under the traditional inoculation strategy, have a positive correlation with tartaric acid *Agrobacterium Larrymoorei* was positively correlated with tartartaric acid, and negatively correlated with pyroglutamic acid, lactic acid and pyruvic acid. *Stenotrophomonas Maltophilia* was negatively correlated with pyroglutamic acid and pyruvic acid. *Xanthomonas Sacchari* was positively correlated with tartartaric acid and negatively correlated with acid. *Agrobacterium larrymoorei*, *Stenotrophomonas maltophilia*, *Xanthomonas Sacchari* were positively correlated with 3mehis, dimethyl ether and ethane, 1,1-diethoxy-and negatively correlated with lAsn, Thr, Ala, a-ABA, Asp., Ile, Cys, Arg, P-ser and Acetoin. *Agrobacterium larrymoorei*, *Stenotrophomonas maltophilia, Xanthomonas Sacchari* under the direct inoculation strategy, were positively correlated with 11 alcohols, 22 esters, 6 aldehydes and 3 acids, and negatively correlated with 6 alcohols, 7 esters, 1 aldehyde and 7 acids, and these strains are negatively correlated with ethyl 4-acetoxybutyloate, Butanoic acid, 3-methyl-, 2-methody-4-vinylphenol, and with hexadecanoic acid, ethyl ester, pexanoic acid, ethyl ester, octanoic acid, ethyl ester, benzoic acid, ethyl ester, heptanoid acid, ethyl ester, 1-Nonal, Pentanoic acid, ethyl ester, Nona Noic acid, ethyl ester and diethyl azelate are positively correlated.

It was well known that fungi play an important role in fermented food (Venturini, 2019). *Saccharomyces Unclassified*, the main fungus under the traditional inoculation strategy is positively correlated with acetic acid, pyruvic acid, Asn, Thr, Ala, a-ABA, Asp., Ile, Cys, Arg, P-ser, Gly, Lys, PEA, and negatively correlated with tartaric acid. While, *Saccharomyces Unclassified*, the main fungus under direct inoculation strategy is positively correlated with both malic acid and acetic acid. Yang et al. (2017) used excellent *Saccharomyces cerevisiae* strains with strong fermentation capacity and strong fragrance production capacity for pure and mixed fermentation, and found that the content of volatile flavor substances could be increased when two excellent yeast strains were mixed for fermentation (Yang et al., 2017). *Alternaria Unclassified* is positively correlated with Gly and Trp. These main fungi are positively correlated with 5 alcohols, 27 esters, 7 aldehydes and 9 acids, among which *Alternaria Unclassified* is positively correlated with phenol alcohol, octanoic acid, exanoic acid, *Saccharomyces Unclassified* is positively correlated with acetic acid, acetoin and ethyl acetate. Although there are few studies on the changes of fungal community during vinegar fermentation, fungi have made a significant contribution to the production of flavor substances in vinegar fermentation. Therefore, in the future research, the role of fungi in the microbial community structure of fermentation and the formation of vinegar flavor quality cannot be ignored (Huang et al., 2022).

## 4. Conclusion

To fully understand the effects of different inoculation strategies on the physicochemical quality and flavor of Zhenjiang aromatic vinegar, we studied the effects of various physicochemical indexes, differences in the composition of flavor substances and microorganisms, and environmental factors on the main microorganisms of Zhenjiang aromatic vinegar for different inoculation strategies during the entire fermentation process. By comparing the physicochemical properties and flavor substances of different inoculation strategies in the fermentation process, it can be found that the content of total acid, organic acid, amino acid and key flavor substances in acetic acid fermentation under the direct inoculation strategy is slightly higher than that under the traditional inoculation strategy. At the same time, the direct inoculation strategy can effectively promote the production of acetoacetate.

The structure and succession pattern of bacterial and fungal communities in the whole fermentation process of two inoculation strategies were studied by using amplified sequence analysis. It was found that inoculation strategies significantly affected the structure of bacterial and fungal communities. The future research should focus more on the role of fungi in the structure of the fermentation microbial community and the generation of vinegar flavor.

Through the correlation analysis between the internal environment of fermented grains and main microorganisms (relative abundance greater than 10%) under different inoculation strategies in the fermentation process, it was found that pH had the most significant effect on microorganisms under different inoculation strategies.

Finally, the Spearman correlation coefficient was used to study the correlation between important microorganisms (average relative abundance>10%) and flavoring substances formed during fermentation under different inoculation strategies. It was found that in the direct inoculation strategy, the correlation between major microbial species and organic acids, non-volatile acids and volatile flavor compounds was more consistent.

The results revealed the effects of different inoculation strategies on the microbial composition and flavor quality during the fermentation of Zhenjiang aromatic vinegar, which will provide useful information for the future development of a direct-injection composite microbial inoculum to replace the traditional starter.

## Data availability statement

The datasets presented in this study can be found in online repositories. The names of the repository/repositories and accession number(s) can be found in the article/Supplementary material.

## Author contributions

XY conducted experiments and made significant contributions to the acquisition, analysis, and interpretation of data. YZ and JL participated in the experiment and revised and discussed the manuscript. KW and PL participated in the revision of the manuscript. YY participated in the experiment and reviewed and agreed to publish this article. ZY and YW designed the study and participated in the drafting and revision of the manuscript. All authors contributed to the article and approved the submitted version.

## Funding

This project was supported by the Postgraduate Research & Practice Innovation Program of Jiangsu Province (No. 1182162204) and the National Natural Science Foundation of China (No. 32072202).

## Conflict of interest

The authors declare that the research was conducted in the absence of any commercial or financial relationships that could be construed as a potential conflict of interest.

## Publisher’s note

All claims expressed in this article are solely those of the authors and do not necessarily represent those of their affiliated organizations, or those of the publisher, the editors and the reviewers. Any product that may be evaluated in this article, or claim that may be made by its manufacturer, is not guaranteed or endorsed by the publisher.

## Abbreviations

AAF: Acetic acid fermentation
HPCLC: High pressure liquid chromatography
OTU: Operation taxonomical unit
NMDS: Non-metric multidimensional scaling
RDA: Redundancy analysis
