# Supplementary material for: Inoculation strategies affect the physicochemical properties and flavor of Zhenjiang aromatic vinegar
Source: Front Microbiol. 2023 Mar 9;14:1126238. doi: 10.3389/fmicb.2023.1126238 (PMC10033837; doi:10.3389/fmicb.2023.1126238)
Supplement: Supplementary file 1 [file Data_Sheet_1.PDF]

Supplementary Material

Inoculation Strategies Affect the Physical and Chemical Quality and Flavor of Zhenjiang Aromatic Vinegar

Ye Xiaoting*, Yu Yongjian, Liu Jiaxin, Yuan-yuan Zhu, Yu Zhen, Liu Peng, Wang Yuqin,Ke Wang

*** Correspondence:** Yu Yongjian: yuyj@just.edu.cn

1 Supplementary Tables

Supplementary Table 1. Dynamic changes of Physicochemical Parameters in Zhenjiang Vinegar Fermentation under Different Inoculation Methods

	Fermentation days(d)	Traditional inoculation			AVG	SD	Direct inoculation			AVG	SD
Total acids (g/100g)	0	1.25	2.04	1.65	1.65	0.40	0.72	0.66	0.69	0.69	0.03
	2	3.74	3.40	3.57	3.57	0.17	0.59	0.66	0.62	0.62	0.04
	4	3.83	4.33	4.08	4.08	0.25	0.90	0.90	0.90	0.90	0.00
	6	4.54	4.65	4.60	4.60	0.06	0.90	1.02	0.96	0.96	0.06
	8	5.51	5.72	5.62	5.62	0.11	1.97	1.68	1.83	1.83	0.15
	10	5.58	5.83	5.71	5.71	0.13	2.70	3.10	2.90	2.90	0.20
	12	5.40	5.42	5.41	5.41	0.01	4.95	4.80	4.88	4.88	0.08
	14	6.08	6.17	6.13	6.13	0.04	6.00	5.70	5.85	5.85	0.15
	16	6.19	6.23	6.21	6.21	0.02	6.60	6.75	6.68	6.68	0.08
	18	6.13	6.22	6.18	6.18	0.04	6.90	6.91	6.91	6.91	0.00
Reducing sugar (g/100g)	0	1.24	1.20	1.22	1.22	0.02	1.20	1.21	1.21	1.21	0.01

	2	1.29	1.21	1.25	1.25	0.04	1.23	1.23	1.23	1.23	0.00
	4	1.33	1.31	1.32	1.32	0.01	1.29	1.28	1.28	1.28	0.01
	6	1.85	1.82	1.84	1.84	0.02	1.30	1.31	1.31	1.31	0.01
	8	2.28	2.24	2.30	2.27	0.03	1.88	1.87	1.88	1.88	0.00
	10	1.75	1.75	1.75	1.75	0.00	2.10	2.10	2.10	2.10	0.00
	12	1.74	1.66	1.66	1.69	0.05	2.28	2.27	2.28	2.28	0.00
	14	1.70	1.65	1.66	1.67	0.03	3.28	3.27	3.27	3.27	0.01
	16	1.65	1.62	1.63	1.63	0.02	3.22	3.22	3.22	3.22	0.00
	18	1.63	1.60	1.60	1.61	0.02	3.12	3.12	3.12	3.12	0.00
**Non-Volatile Acids (g/100g)**	0	0.14	0.14	0.14	0.14	0.00	0.42	0.42	0.42	0.42	0.00
	2	0.17	0.19	0.18	0.18	0.01	0.66	0.66	0.65	0.66	0.01
	4	0.17	0.24	0.20	0.20	0.04	1.00	1.00	1.00	1.00	0.00
	6	0.33	0.27	0.30	0.30	0.03	1.22	1.12	1.10	1.15	0.06
	8	0.37	0.32	0.35	0.35	0.03	1.30	1.32	1.30	1.31	0.01
	10	0.37	0.35	0.36	0.36	0.01	1.40	1.40	1.40	1.40	0.00
	12	0.41	0.40	0.41	0.41	0.01	1.58	1.58	1.58	1.58	0.00
	14	0.38	0.39	0.39	0.39	0.01	1.28	1.30	1.26	1.28	0.02
	16	0.33	0.30	0.31	0.31	0.02	1.40	1.52	1.42	1.45	0.06
	18	0.32	0.26	0.29	0.29	0.03	1.56	1.55	1.58	1.56	0.02
**Temperature in vinegar Pei**	0	24.10	24.50	24.50	24.37	0.23	26.30	26.00	26.70	26.33	0.35

(℃)										
	2	36.50	35.60	37.50	36.53	0.95	41.62	40.00	40.55	40.72	0.82
	4	43.20	43.20	44.00	43.47	0.46	40.10	40.30	40.10	40.17	0.12
	6	45.50	45.00	44.80	45.10	0.36	45.30	45.00	44.95	45.08	0.19
	8	47.20	46.00	45.00	46.07	1.10	46.70	44.00	42.00	44.23	2.36
	10	38.80	38.00	39.80	38.87	0.90	49.85	47.10	47.53	48.16	1.48
	12	42.00	35.00	36.00	37.67	3.79	48.00	48.00	48.00	48.00	0.00
	14	30.00	27.90	29.20	29.03	1.06	44.70	45.00	45.20	44.97	0.25
	16	30.10	28.60	29.80	29.50	0.79	45.20	44.00	44.40	44.53	0.61
	18	30.20	26.00	25.80	27.33	2.48	35.30	35.00	35.70	35.33	0.35

Supplementary Table 2. Dynamic Changes of oganic acids in Zhenjiang Vinegar Fermentation under Different Inoculation Methods.

	Traditional inoculation(mg/100g)										Direct inoculation(mg/100g)									
**Fermentation days(d)**	0	2	4	6	8	10	12	14	16	18	0	2	4	6	8	10	12	14	16	18
	0.07	0.12	0.17	0.23	0.21	0.19	0.10	0.16	0.13	0.18	0.19	0.34	0.42	0.49	0.45	0.46	0.37	0.46	0.43	0.41
**oxalic acid**	0.11	0.19	0.19	0.21	0.25	0.24	0.12	0.13	0.13	0.26	0.19	0.41	0.53	0.50	0.43	0.45	0.42	0.45	0.43	0.39
	0.04	0.18	0.19	0.21	0.27	0.18	0.10	0.17	0.12	0.18	0.18	0.43	0.56	0.48	0.42	0.52	0.41	0.46	0.42	0.40
**tartaric acid**	0.96	1.93	1.14	1.71	1.12	0.95	0.71	1.15	1.10	1.34	0.92	1.66	1.78	2.00	1.46	1.92	1.77	2.14	1.95	2.07

	1.87	2.17	1.17	1.11	1.10	1.10	0.80	0.71	1.06	1.57	0.65	2.48	2.60	1.98	1.54	1.95	1.91	2.12	2.04	2.06
	1.42	2.18	2.20	1.10	1.13	0.95	0.74	0.72	1.11	1.31	0.67	2.60	2.39	1.99	1.57	2.17	1.93	2.11	1.97	2.25
	1.08	1.15	1.27	5.07	5.66	8.22	7.77	7.91	7.42	8.61	0.44	0.84	0.57	0.62	0.71	1.67	1.01	0.81	0.76	0.68
**lactic acid**	1.09	0.81	1.38	5.65	7.76	7.71	6.97	7.92	7.43	8.72	0.79	0.60	0.54	0.61	1.06	1.44	1.08	0.71	0.75	0.87
	1.08	0.79	1.27	5.66	7.79	8.18	8.31	7.95	7.36	8.60	0.83	0.69	0.54	1.07	1.17	1.46	4.59	0.82	0.77	0.94
	0.75	0.78	4.54	6.37	6.34	11.19	11.31	12.32	13.54	15.19	0.23	1.88	2.55	5.11	6.79	10.66	11.75	15.73	15.01	12.75
**acetic acid**	0.79	1.36	3.56	6.32	10.89	10.82	12.21	12.29	13.56	15.38	0.88	1.68	3.35	5.15	6.78	10.27	11.61	15.58	15.77	17.69
	0.77	1.24	4.19	6.38	10.89	11.09	12.79	12.33	13.42	15.13	0.92	1.55	2.61	5.58	6.80	10.41	12.62	15.60	15.10	17.93
	3.08	2.89	3.73	4.48	3.87	3.69	3.65	3.70	3.36	3.64	0.95	4.50	7.20	8.09	8.20	9.03	7.92	9.04	8.83	8.81
**citric acid**	3.05	3.34	4.53	4.06	4.33	4.20	3.65	3.90	3.42	3.77	3.39	4.61	6.67	8.18	8.22	9.02	7.62	9.05	8.81	8.66
	3.07	3.33	4.06	3.58	4.15	4.08	3.63	3.79	3.29	3.72	3.35	4.58	6.65	8.96	8.22	9.16	7.99	9.11	8.77	8.26
	0.01	0.12	0.15	0.30	0.40	0.47	0.48	0.51	0.47	0.61	0.45	0.19	0.36	0.29	0.59	0.81	0.99	1.16	1.10	1.06
**pyroglutamic acid**	0.01	0.12	0.20	0.30	0.59	0.58	0.49	0.53	0.47	0.55	0.03	0.20	0.23	0.30	0.53	0.83	0.92	1.15	1.10	1.04
	0.01	0.12	0.12	0.31	0.57	0.60	0.49	0.52	0.46	0.55	0.03	0.19	0.22	0.46	0.60	0.87	0.99	1.16	1.09	0.99
	0.04	0.04	0.14	0.11	0.11	0.16	0.13	0.17	0.16	0.19	0.03	0.03	0.12	0.14	0.15	0.15	0.14	0.13	0.13	0.12
**pyruvic acid**	0.04	0.07	0.09	0.12	0.16	0.16	0.30	0.16	0.16	0.38	0.05	0.03	0.12	0.14	0.11	0.12	0.13	0.14	0.13	0.13
	0.04	0.06	0.06	0.12	0.16	0.16	0.15	0.17	0.16	0.19	0.05	0.03	0.11	0.14	0.08	0.13	0.13	0.15	0.12	0.12

		0.03	0.05	0.76	0.11	0.53	0.24	0.07	0.03	0.02	0.00	0.10	0.27	0.31	0.78	0.81	1.25	1.01	1.19	1.10	0.88
**malic acid**		0.06	0.02	0.70	0.12	0.44	0.46	0.04	0.03	0.02	0.01	0.10	0.25	0.30	0.82	0.85	1.40	0.94	1.19	0.91	0.82
		0.05	0.03	0.02	0.12	0.43	0.21	0.09	0.05	0.02	0.04	0.08	0.21	0.27	0.85	0.84	1.25	1.08	1.26	1.10	1.03
		0.99	1.26	3.22	3.03	3.13	2.37	2.07	2.34	2.22	2.14	2.50	4.36	5.15	5.37	4.93	4.19	2.99	4.12	2.82	2.48
**succinic acid**		1.01	2.11	3.22	2.99	2.77	2.92	2.42	2.15	2.22	2.40	2.48	4.52	5.11	5.31	4.81	4.54	3.13	4.12	2.85	2.34
		1.00	2.08	2.07	2.89	3.02	2.32	2.38	2.53	2.27	2.31	2.57	4.15	5.07	5.31	4.83	4.33	3.94	5.03	3.81	2.18
**Total content**	**AVG**	7.50	9.51	14.78	20.89	26.02	27.82	27.33	28.11	28.36	32.33	7.69	14.43	18.77	23.57	24.32	30.15	29.80	34.99	32.69	32.45
	**SD**	0.06	0.11	0.06	0.06	0.45	0.04	0.14	0.03	0.01	0.07	0.18	0.04	0.06	0.12	0.03	0.02	0.37	0.07	0.06	0.31

Supplementary Table 3. Dynamic Changes of amino acids in Zhenjiang Vinegar Fermentation under Different Inoculation Methods.

	Traditional inoculation(mg/100g)										Direct inoculation(mg/100g)									
**Fermentation days(d)**	**0**	**2**	**4**	**6**	**8**	**10**	**12**	**14**	**16**	**18**	**0**	**2**	**4**	**6**	**8**	**10**	**12**	**14**	**16**	**18**
	67.19	97.13	109.26	117.03	128.47	138.27	164.09	149.80	153.46	173.00	2.52	81.01	125.32	126.57	146.56	168.84	173.38	164.27	158.06	168.10
**Asp**	81.55	100.39	107.43	127.70	131.08	130.56	145.13	149.80	153.76	173.00	2.96	98.06	123.14	121.72	131.98	168.38	161.70	118.74	159.80	169.07
	47.55	93.42	105.29	120.45	129.77	142.40	154.61	149.80	153.76	173.00	2.55	86.83	120.96	135.76	150.39	150.93	168.27	152.09	153.64	178.04
**Thr**	34.25	64.22	67.60	85.29	98.08	109.30	129.48	111.79	116.92	321.33	37.99	63.82	96.19	103.19	119.00	136.84	142.50	128.71	123.72	125.97

	39.70	65.92	75.75	76.99	95.02	112.58	120.26	111.79	111.47	321.33	42.04	78.12	94.20	97.20	88.06	134.45	130.74	95.11	113.46	130.68
	36.55	60.11	70.59	83.63	96.55	110.14	124.87	111.79	111.47	321.33	41.40	69.42	82.94	109.81	125.59	122.85	136.07	116.06	123.88	137.26
	48.93	96.02	97.48	125.91	147.25	163.52	194.17	168.86	180.16	7.00	56.61	94.29	139.45	144.83	167.21	192.33	202.29	181.07	176.37	179.39
**Ser**	57.34	98.06	109.58	125.61	149.70	170.02	180.50	168.86	174.78	7.00	62.88	115.70	136.52	136.37	137.57	195.29	187.90	134.80	167.91	189.41
	39.07	89.61	101.27	126.55	148.47	169.27	187.33	168.86	174.78	7.00	61.82	103.48	127.46	155.39	176.67	173.26	198.99	166.06	180.06	199.69
	69.83	107.84	134.97	173.87	209.47	233.40	271.24	228.15	244.60	255.76	60.55	109.64	182.72	198.25	225.54	260.01	270.85	240.00	236.37	238.99
**Asn**	80.11	112.17	151.81	124.88	205.73	244.25	247.58	228.15	238.74	236.50	67.82	131.39	178.94	182.31	195.13	264.21	251.37	182.08	224.28	251.02
	54.91	98.39	137.39	171.90	207.60	234.98	259.41	228.15	238.74	256.50	66.66	116.85	171.36	212.11	240.16	236.79	264.83	205.91	244.92	266.92
	74.32	167.37	111.38	136.60	131.82	114.99	125.35	73.62	98.71	86.27	52.26	117.51	151.08	127.73	110.64	80.11	73.71	0.00	0.00	0.00
**Glu**	60.39	172.17	157.80	182.48	87.27	130.27	110.05	73.62	99.28	88.63	62.14	148.72	150.20	102.32	100.47	96.00	62.73	0.00	0.00	0.00
	105.70	104.78	92.29	97.99	109.54	106.73	117.70	73.62	99.28	86.81	60.95	126.37	145.26	137.94	129.72	86.16	66.99	0.00	0.00	0.00
	36.82	63.29	80.83	84.98	98.64	110.26	125.65	115.34	117.66	115.61	38.84	57.00	82.76	90.94	106.41	126.32	126.87	122.90	113.22	121.37
**Gly**	35.44	64.08	34.07	246.07	97.66	113.37	115.97	115.34	116.57	115.54	42.57	64.95	81.04	86.53	94.00	125.00	116.97	86.23	111.60	120.25
	169.20	53.90	64.27	79.63	98.15	105.53	120.81	115.34	116.57	116.01	41.98	61.40	80.29	95.74	106.59	112.79	120.45	106.16	108.52	119.06
	55.99	96.26	126.84	153.64	189.38	215.41	251.21	231.46	236.15	231.46	88.99	94.58	127.44	129.48	149.59	176.11	181.10	176.20	166.25	174.60
**Ala**	58.20	98.64	88.34	92.76	191.98	222.01	232.00	231.46	233.61	231.46	98.10	108.11	124.43	126.21	133.33	171.86	167.76	0.00	163.33	176.06
	31.54	84.79	101.87	149.99	190.68	212.21	241.60	231.46	233.61	231.46	96.86	101.87	123.25	139.91	151.55	156.90	173.54	156.50	157.78	174.40
	4.97	6.37	11.66	9.25	8.84	10.75	6.73	10.38	7.28	8.33	7.78	8.83	7.63	12.58	9.33	19.50	16.41	15.99	17.05	13.76
**Val**	0.22	6.17	10.83	169.40	6.73	3.08	9.70	6.38	3.45	3.69	7.82	8.25	9.18	14.52	11.92	13.28	11.27	10.68	10.92	12.93
	54.51	6.06	6.55	6.06	7.78	7.00	8.22	6.38	6.45	6.36	8.34	6.60	10.60	15.55	15.47	17.83	13.19	9.22	12.00	12.16

	39.32	87.82	91.89	114.65	138.83	156.69	186.46	155.37	171.28	5.00	53.94	98.85	152.83	132.37	177.06	184.55	211.11	209.33	201.86	219.16
**Met**	55.47	83.00	85.86	5.23	143.74	146.67	162.71	155.37	162.94	5.00	58.13	126.17	149.22	147.06	159.49	201.11	190.41	153.46	202.30	214.00
	17.27	69.20	98.15	120.19	141.28	165.70	174.58	155.37	162.94	5.00	63.94	112.64	147.19	168.54	179.49	175.97	182.08	96.91	190.58	212.71
	76.52	191.53	191.33	246.87	279.98	304.85	352.16	308.57	315.87	303.56	74.57	182.71	301.01	254.29	356.66	389.25	422.68	417.31	410.07	427.00
**Ile**	99.94	154.59	203.24	63.85	292.08	308.95	307.66	308.57	303.10	301.21	90.96	237.84	293.91	298.31	318.43	406.64	360.59	299.62	398.53	427.32
	57.96	132.32	219.51	254.03	286.03	311.70	329.91	308.57	303.10	301.52	97.22	214.52	292.33	338.82	359.23	357.49	373.78	160.41	379.61	422.04
	7.90	7.21	21.17	29.36	25.01	24.23	19.19	13.06	11.56	10.20	4.92	29.25	87.34	37.66	108.76	95.33	76.01	102.93	99.74	112.50
**Leu**	7.80	6.93	29.75	29.34	24.22	21.77	16.32	13.06	12.29	10.52	4.53	76.04	82.12	89.59	96.48	114.92	36.83	76.11	86.17	88.78
	7.13	8.52	26.30	31.80	24.62	22.47	17.75	13.06	13.58	10.20	7.90	67.69	86.28	100.31	97.72	81.98	34.69	402.44	82.67	88.08
	39.38	142.62	142.30	77.02	78.66	83.39	59.75	64.60	64.33	64.34	55.17	90.06	229.74	197.94	294.47	308.05	263.97	311.48	306.14	319.48
**Tyr**	36.43	105.49	138.65	125.85	82.22	79.23	65.70	64.60	266.90	126.33	71.66	186.81	219.52	237.18	245.84	329.68	273.04	226.09	295.38	310.68
	49.61	70.85	85.75	104.91	80.44	63.59	62.72	64.60	66.90	67.63	67.81	160.33	225.40	264.28	287.89	252.02	281.02	0.00	283.03	310.33
	3.35	7.13	14.37	1.45	0.00	0.00	6.33	6.28	5.80	5.31	1.56	5.22	9.10	12.99	10.79	12.86	7.71	5.62	33.25	8.26
**Phe**	0.21	7.57	11.51	7.68	4.08	2.64	6.33	6.68	7.48	5.68	3.91	7.83	10.74	8.61	8.74	14.77	14.98	7.72	0.00	1.50
	71.15	14.95	28.35	7.53	4.08	21.04	6.33	6.58	6.49	6.05	2.53	6.33	12.00	6.10	11.42	17.95	13.11	13.50	0.00	32.84
	23.82	44.14	32.04	35.90	30.87	41.62	47.49	39.09	38.50	45.22	19.76	27.45	28.49	24.85	26.80	28.35	25.95	27.34	25.72	26.47
**Gaba**	24.04	50.11	32.59	36.09	38.24	42.84	41.10	39.09	45.23	45.25	21.11	32.98	27.83	23.18	22.94	27.67	26.72	21.70	24.95	27.20
	24.00	42.41	52.30	35.02	34.55	39.52	44.29	39.09	45.23	45.22	21.03	29.21	27.23	27.38	23.68	22.23	26.39	33.59	24.06	26.59
**His**	448.58	295.26	526.70	159.07	624.59	488.58	802.66	480.63	435.20	438.67	428.30	524.20	607.73	616.19	699.35	776.78	636.04	737.39	494.41	692.23
	100.02	573.72	582.53	2.00	303.19	506.06	520.20	480.63	493.26	496.34	288.83	531.35	557.88	529.78	614.18	796.32	735.99	548.07	497.57	480.40

		22.71	132.33	2.26	293.34	463.89	381.76	661.43	480.63	493.26	493.28	317.21	528.51	580.74	699.49	668.29	656.37	615.90	729.98	490.59	415.15
		116.03	0.53	90.78	70.30	105.50	111.52	111.50	87.53	99.32	105.76	105.36	92.73	113.72	103.85	70.48	102.38	22.08	54.39	54.49	71.75
**Trp**		76.53	0.50	73.73	40.73	76.86	74.95	106.91	87.53	106.70	99.66	115.83	82.03	106.28	68.14	62.64	86.32	52.67	39.12	38.86	36.76
		63.33	68.32	0.00	66.68	91.18	90.53	109.20	87.53	106.70	104.24	96.00	111.63	113.10	73.63	77.65	103.09	64.72	53.07	66.46	64.65
		5.80	101.87	8.05	5.64	7.54	7.75	10.46	9.83	10.58	10.53	1.44	2.12	3.57	4.15	5.53	20.88	24.60	23.12	22.35	22.96
**Orn**		9.24	88.36	7.35	5.00	11.40	8.68	10.30	9.83	9.12	10.36	1.97	2.84	3.25	3.81	4.56	19.75	19.50	13.25	22.80	25.46
		7.76	9.36	276.67	9.65	9.47	11.53	10.38	9.83	9.12	9.14	1.98	2.37	3.60	3.75	5.14	19.05	23.33	22.37	19.02	22.79
		65.64	13.62	182.20	182.05	198.17	203.94	235.78	210.93	212.27	212.58	65.15	97.76	175.80	185.76	217.57	227.62	249.02	242.86	237.93	234.72
**Lys**		68.75	12.53	157.34	711.01	201.64	221.34	219.07	210.93	215.61	212.32	75.52	133.34	157.66	166.44	168.42	249.16	233.83	171.96	231.66	254.96
		77.62	105.93	149.33	186.36	199.90	220.55	227.43	210.93	215.61	213.60	71.80	84.31	137.24	191.76	210.08	215.43	23.33	223.16	224.70	248.47
		115.66	181.67	311.83	306.83	368.01	415.54	456.62	416.89	437.55	425.89	125.92	190.74	312.37	335.68	412.69	460.16	468.13	451.38	415.70	443.62
**Arg**		117.89	202.59	209.04	362.34	372.97	416.06	429.13	416.89	387.22	316.57	146.22	238.35	299.79	325.00	355.27	460.80	398.61	268.86	409.95	468.68
		0.00	168.83	0.00	350.75	370.49	424.05	442.87	416.89	387.22	324.67	136.62	183.23	291.45	9.00	403.51	397.55	243.59	400.77	393.83	453.84
**Total content**	**AVG**	1093.71	1729.66	2079.34	2315.73	2693.13	2910.01	3301.46	2879.75	3014.51	2803.74	1270.41	2183.41	2839.60	2829.61	3261.36	3666.18	3350.75	3038.03	3195.84	3456.83
	**SD**	11.13	6.58	11.59	11.58	10.14	3.21	13.41	0.11	5.80	1.24	0.51	11.62	4.37	3.21	14.22	14.40	15.47	30.49	4.46	6.54

Supplementary Table 4. Dynamic Changes of Flavor Substances Metabolized by Microorganisms during Zhenjiang Vinegar Fermentation under Different Inoculation Methods.

Volatile flavor substances （mg/100g）	0-1	0-2	0-3	2-1	2-2	2-3	4-1	4-2	4-3	6-1	6-2	6-3	8-1	8-2	8-3	1 0-	1 0-	1 0-	12 -1	12 -2	1 2-	1 4-	1 4-	1 4-	1 6-	1 6-	1 6-	18 -1	18 -2	18 -3

																	1	2	3			3	1	2	3	1	2	3			
Traditional inoculation	Acetic acid ethenyl ester	0.00	0.00	0.00	0.00	0.00	0.00	0.00	0.00	0.00	0.00	0.00	0.00	0.00	0.00	0.00	0.00	0.00	0.00	0.00	0.00	0.00	0.00	0.00	0.00	0.00	0.00	0.00	0.37	5.45	0.56
	Acetic acid, 2-phenylethyl ester	0.00	0.00	0.00	0.01	0.01	0.01	0.93	1.11	2.23	0.24	7.70	4.24	28.55	0.00	0.00	0.00	0.00	0.00	0.00	0.00	0.00	10.65	11.02	10.65	0.00	0.00	0.00	30.62	38.80	39.49
	Acetic acid, butyl ester	0.00	0.00	0.00	0.00	0.00	0.00	0.00	0.00	0.00	0.00	0.00	0.00	0.00	0.00	0.00	0.00	0.00	0.00	0.00	0.00	0.00	0.04	0.11	0.04	0.00	0.00	0.00	0.05	0.00	0.00
	Acetic acid, hexyl ester	0.00	0.00	0.00	0.00	0.00	0.00	0.00	0.00	0.00	0.01	0.38	0.35	0.57	0.55	0.00	0.00	0.00	0.00	0.00	0.00	0.00	0.14	0.16	0.14	0.51	0.51	0.00	0.21	0.33	0.00
	Acetic acid, methyl ester	0.00	0.00	0.00	0.00	0.00	0.00	0.00	0.00	0.00	0.00	0.00	0.00	0.00	0.00	0.00	0.00	0.00	0.00	0.00	0.00	0.00	0.03	0.07	0.03	0.00	0.00	0.00	0.10	0.86	0.10
	Acetic acid, octyl ester	0.00	0.00	0.00	0.00	0.00	0.00	0.00	0.00	0.00	0.00	0.00	0.00	0.00	0.00	0.00	0.00	0.00	0.00	0.00	0.00	0.00	0.05	0.07	0.05	0.00	0.00	0.00	0.00	0.13	0.19
	Benzeneacetic acid, ethyl ester	0.00	0.00	0.00	0.03	0.03	0.03	0.80	0.93	1.93	0.02	0.76	0.38	1.49	1.41	1.33	2.12	0.02	2.02	2.33	2.07	0.02	0.30	0.39	0.30	1.05	1.05	2.21	0.94	6.97	1.25
	Benzoic acid, ethyl ester	0.19	0.18	0.77	0.04	0.03	0.03	0.85	0.95	1.68	0.01	0.41	0.27	0.70	0.64	0.66	0.00	0.90	0.81	1.02	0.94	0.00	0.58	0.67	0.58	0.53	0.53	0.89	2.76	8.30	3.88
	Butanedioic acid, diethyl ester	0.00	0.00	0.00	0.13	0.14	0.14	9.40	10.79	25.00	0.43	13.11	6.97	7.68	7.81	7.22	7.55	0.11	7.80	7.18	6.06	0.07	0.00	0.00	0.00	2.77	2.77	7.34	0.00	0.00	0.00
	Butanoic acid, ethyl ester	0.00	0.00	0.00	0.00	0.00	0.00	0.00	0.00	0.00	0.00	0.00	0.00	0.00	0.00	0.00	0.00	0.00	0.00	0.00	0.00	0.00	0.02	0.02	0.02	0.00	0.00	0.00	0.00	0.00	0.00
	Decanoic acid, ethyl ester	0.00	0.00	0.00	0.02	0.02	0.02	0.65	0.85	1.06	0.00	0.00	0.31	0.80	0.55	0.00	0.00	0.00	0.00	0.00	0.00	0.00	0.00	0.00	0.06	0.39	0.39	0.25	0.00	0.00	0.00

Dodecanoic acid, ethyl ester	0.00	0.00	0.00	0.00	0.00	0.00	0.00	0.00	0.37	0.59	0.68	0.00	0.00	0.00	0.00	0.00	0.00	0.00	0.00	0.00	0.00	0.00	0.00	0.00	0.00	0.00	0.00	0.00	0.00	0.00
Formic acid, 1-methylethyl ester	0.00	0.00	0.00	0.00	0.00	0.00	0.00	0.00	0.00	0.00	0.00	0.00	1.47	1.40	1.32	0.00	0.00	0.00	0.00	0.00	0.00	0.00	0.00	0.00	0.00	0.00	0.00	0.00	0.00	0.00
Formic acid, hexyl ester	0.31	0.34	0.00	0.00	0.00	0.00	0.00	0.00	0.00	0.00	0.00	0.00	0.00	0.00	0.00	0.00	0.00	0.00	0.00	0.00	0.00	0.08	0.08	0.00	0.00	0.00	0.00	0.00	0.00	0.00
Heptanoic acid, ethyl ester	0.00	0.00	0.00	0.00	0.00	0.00	0.35	0.38	0.00	0.00	0.00	0.00	0.00	0.00	0.00	0.00	0.00	0.00	0.00	0.00	0.00	0.00	0.00	0.00	0.00	0.00	0.00	0.00	0.00	0.00
Hexadecanoic acid, ethyl ester	0.52	1.74	3.15	0.10	0.11	0.11	2.43	2.85	8.20	0.08	2.65	1.02	2.78	2.25	2.28	2.29	0.03	1.85	2.32	1.67	0.02	0.45	0.99	0.45	0.64	0.64	1.65	2.76	16.42	3.02
Hexanoic acid, ethyl ester	1.16	2.85	2.98	0.09	0.06	0.05	4.45	4.72	4.17	0.03	1.00	1.77	0.94	0.75	0.81	0.75	0.00	0.00	0.00	0.00	0.00	0.12	0.12	0.12	0.68	0.68	0.00	0.00	0.36	0.23
Linoleic acid ethyl ester	0.00	0.00	0.00	0.00	0.00	0.00	0.00	0.00	0.00	0.00	0.00	0.34	0.59	0.50	0.00	0.00	0.00	0.00	0.00	0.00	0.00	0.00	0.00	0.00	0.00	0.00	0.00	0.00	0.00	0.00
Nonanoic acid, ethyl ester	0.00	0.00	0.00	0.02	0.01	0.02	0.72	0.85	0.00	0.20	0.00	0.00	0.00	0.00	0.00	0.00	0.00	0.00	0.00	0.00	0.00	0.15	0.08	0.15	0.00	0.00	0.00	0.00	0.00	0.00
Pentanoic acid, ethyl ester	0.00	0.00	0.00	0.00	0.00	0.00	0.00	0.48	0.52	0.22	0.00	0.00	0.00	0.00	0.00	0.00	0.00	0.00	0.00	0.00	0.00	0.00	0.00	0.00	0.00	0.00	0.00	0.00	0.00	0.00
Propanoic acid, 2-hydroxy-, ethyl ester	0.00	0.00	0.00	0.00	0.00	0.00	0.00	0.00	0.00	0.02	0.70	0.39	2.29	2.25	1.99	3.01	0.04	3.10	2.60	1.94	0.03	0.00	0.00	0.00	0.91	0.91	2.21	0.00	0.00	0.00
Trichloroacetic acid, dodec-9-ynyl ester	0.05	0.29	0.00	0.00	0.00	0.00	0.00	0.00	0.00	0.00	0.00	0.00	0.00	0.00	0.00	0.00	0.00	0.00	0.00	0.00	0.00	0.00	0.00	0.00	0.00	0.00	0.00	0.00	0.00	0.00
2-Propenoic acid, 2-methyl-, hexyl ester	0.00	0.00	0.00	0.00	0.00	0.00	0.00	0.00	0.00	0.00	0.00	0.00	0.00	0.00	0.00	0.00	0.00	0.00	0.00	0.00	0.00	0.00	0.00	0.00	45.9	57.0	49.6	0.00	0.00	0.00

																									6	3	7			
3-Methyl-hepta-1,6-dien-3-ol	0.00	0.00	0.00	0.00	0.00	0.00	0.00	0.00	0.00	0.00	0.00	0.00	0.00	0.00	0.00	0.00	0.00	0.00	0.00	0.00	0.00	0.00	0.00	0.00	0.00	0.00	0.00	0.17	1.03	0.15
Diethyl azelate	0.00	0.00	0.00	0.00	0.01	0.01	0.27	0.47	0.90	0.01	0.44	0.33	0.00	0.00	0.00	0.00	0.00	0.00	0.00	0.00	0.00	0.00	0.00	0.00	0.00	0.00	0.00	0.00	0.00	0.00
Ethyl 4-acetoxybutanoate	0.00	0.00	0.00	0.00	0.00	0.00	0.00	0.00	0.00	0.00	0.00	0.32	0.96	0.94	0.88	1.25	0.02	1.27	1.78	1.31	0.02	0.02	0.08	0.02	0.62	0.62	1.58	0.07	0.46	0.13
Ethyl Acetate	1.34	2.94	2.49	0.17	0.18	0.17	0.00	0.00	0.00	0.00	0.00	0.00	0.00	0.00	0.00	0.92	0.00	0.00	0.73	0.00	0.00	0.82	0.99	0.98	0.47	0.47	1.28	0.00	0.00	0.00
Isobutyl acetate	0.00	0.00	0.00	0.00	0.00	0.00	0.65	0.71	1.00	0.06	2.24	2.04	5.20	5.16	4.78	7.04	0.08	7.44	9.41	6.66	0.07	0.76	0.95	0.76	5.72	5.72	7.18	1.69	30.15	1.71
n-Propyl acetate	0.00	0.00	0.00	0.00	0.00	0.00	0.00	0.00	0.00	0.00	0.31	0.22	0.85	0.83	0.72	0.96	0.01	1.06	1.43	1.05	0.01	0.11	0.11	0.11	0.70	0.70	1.49	0.00	0.00	0.00
Alanine	0.00	0.00	0.00	0.00	0.00	0.00	0.00	0.00	0.00	0.00	0.00	0.00	0.00	0.00	0.00	0.00	0.00	0.00	0.00	0.00	0.00	0.04	0.07	0.04	0.00	0.00	0.00	0.00	0.00	0.00
Acetic acid	0.00	0.00	0.00	0.00	0.00	0.00	0.00	1.08	1.07	0.26	8.61	4.81	40.58	39.00	34.35	56.56	60.84	60.66	75.27	69.01	60.96	71.90	73.27	71.90	84.37	84.37	80.46	101.62	129.20	126.98
Hexanoic acid	0.00	0.00	0.00	0.00	0.00	0.00	0.00	0.00	0.00	0.00	0.00	0.00	0.00	0.00	0.00	0.00	0.00	0.00	5.34	4.14	0.00	0.00	0.00	0.00	1.76	1.76	4.43	0.00	0.00	0.00
Octanoic acid	0.00	0.00	0.00	0.00	0.00	0.00	0.00	0.00	0.00	0.00	0.00	0.00	0.00	0.00	0.00	0.00	0.00	0.00	1.29	1.02	0.01	0.00	0.00	0.00	0.50	0.50	1.25	0.00	0.00	0.00
Pentanoic acid	0.00	0.00	0.00	0.00	0.00	0.00	0.00	0.00	0.00	0.00	0.00	0.00	1.98	2.10	1.90	2.94	0.04	3.01	0.05	0.00	0.00	0.00	0.00	0.00	0.00	0.00	0.00	0.00	0.00	0.00

Pentanoic acid, 3-methyl-	0.00	0.00	0.00	0.00	0.00	0.00	0.00	0.00	0.00	0.00	0.00	0.00	0.00	0.00	0.00	0.00	0.00	0.00	0.00	0.00	0.00	0.04	0.04	0.00	0.00	0.00	0.00	0.00	0.00	1.36
Undecylenic acid	0.32	0.25	0.00	0.00	0.00	0.00	0.00	0.00	0.00	0.00	0.00	0.00	0.00	0.00	0.00	0.00	0.00	0.00	0.00	0.00	0.00	0.00	0.00	0.00	0.00	0.00	0.00	0.00	0.00	0.00
Benzenepropanoic acid, .alpha.-(1-hydroxyethyl)-	0.00	0.00	0.00	0.00	0.00	0.00	0.00	0.00	0.00	0.00	0.00	0.00	0.00	0.00	0.00	0.00	0.00	0.00	0.00	0.00	0.00	0.00	0.00	0.00	0.00	0.00	0.00	0.63	3.95	0.82
Butanoic acid, 3-methyl-	0.00	0.00	0.00	0.00	0.00	0.00	0.00	0.00	0.00	0.00	0.00	0.00	0.00	0.00	0.00	5.16	0.07	5.06	8.67	6.42	0.09	0.00	0.00	0.00	3.18	3.18	7.99	0.93	2.39	0.00
Propanoic acid, 2-methyl-	0.00	0.00	0.00	0.00	0.00	0.00	0.00	0.00	0.00	0.00	0.00	0.00	0.00	0.00	0.00	0.00	1.30	1.25	2.64	2.00	0.03	0.00	0.00	0.00	1.21	1.21	3.06	0.00	0.00	0.00
Propanoic acid, anhydride	0.00	0.00	0.00	0.11	0.12	0.00	0.00	0.00	0.00	0.00	0.00	0.00	3.99	3.64	0.00	0.00	0.00	0.00	0.00	0.00	0.00	0.00	0.00	0.00	0.00	0.00	0.00	0.00	0.00	0.00
17-Octadecynoic acid	0.00	0.00	0.00	0.00	0.00	0.00	0.00	0.00	0.00	0.00	0.00	0.00	0.00	0.00	0.00	0.00	0.00	0.00	0.00	0.00	0.00	0.08	0.06	0.06	0.00	0.00	0.00	0.00	0.00	0.00
1-Hexanol	0.00	0.00	0.00	0.06	0.06	0.06	1.71	1.91	4.31	0.04	1.27	0.70	1.44	1.49	1.32	1.68	0.02	1.68	1.66	1.31	0.02	0.00	0.00	0.00	0.53	0.53	1.23	0.00	0.00	0.00
1-Propanol, 2-methyl-	2.85	5.41	3.85	0.19	0.20	0.19	6.09	6.42	14.85	0.16	5.30	3.00	7.43	7.27	6.43	7.64	0.11	8.05	7.66	5.70	0.08	0.25	0.74	0.25	2.37	2.37	5.50	0.48	3.99	1.11
1-Butanol, 3-methyl-	9.89	26.22	23.04	0.83	0.72	0.69	25.27	27.20	62.88	0.70	16.80	11.38	30.67	25.05	27.04	26.30	0.40	34.03	34.81	25.85	0.36	1.52	2.50	1.52	9.00	9.00	26.12	3.67	16.88	4.65
1-Butanol, 3-methyl-, acetate	0.00	0.00	0.00	0.03	0.03	0.02	3.04	3.56	4.80	0.28	9.91	9.28	18.97	18.33	18.08	24.14	0.25	23.83	26.94	21.41	0.20	2.84	4.16	2.84	20.52	20.52	0.00	3.85	9.21	7.82

2,3-Butanediol, [S-(R*,R*)]-	0.00	0.00	0.00	0.00	0.00	0.00	0.00	0.00	0.00	0.02	0.60	0.30	3.06	3.10	2.61	5.81	0.09	6.16	15.72	10.93	0.15	0.00	0.00	0.00	6.39	6.39	16.08	0.00	0.00	0.00
Creosol	0.00	0.00	0.00	0.00	0.00	0.00	0.00	0.00	0.00	0.00	0.00	0.00	0.00	0.00	0.00	0.00	0.00	0.00	0.00	0.00	0.00	0.09	0.12	0.09	0.00	0.00	0.00	0.39	2.39	0.57
Ethanol	0.00	0.00	0.00	0.02	0.02	0.37	0.59	0.33	1.86	0.00	0.01	18.73	51.60	50.09	44.90	47.10	0.70	49.40	33.60	24.25	0.34	0.00	0.00	0.00	8.57	8.57	20.37	0.00	0.00	0.00
Cyclobutanol	0.00	0.00	0.00	0.00	0.00	0.00	0.00	0.00	0.00	0.00	0.00	0.00	0.00	0.00	0.00	0.00	0.00	0.00	0.00	0.00	0.00	0.00	0.00	0.00	0.00	0.00	0.00	0.06	0.06	0.06
Phenylethyl Alcohol	15.99	33.17	35.40	0.00	0.00	0.00	0.00	0.00	0.00	0.00	0.00	0.00	0.00	0.00	0.00	0.00	0.00	0.00	0.00	0.00	0.00	6.26	7.33	6.26	0.00	0.00	0.00	23.19	32.22	34.05
Benzaldehyde	0.00	0.00	0.00	0.00	0.00	0.00	0.00	0.00	0.00	0.02	0.60	0.36	3.18	3.18	2.81	0.89	0.01	0.91	0.79	0.00	0.00	0.09	0.38	0.09	0.39	0.39	0.87	0.97	1.78	0.49
Benzeneacetaldehyde	0.04	0.10	0.10	0.00	0.00	0.00	0.32	0.34	0.83	0.01	0.31	0.00	0.00	0.00	0.00	0.00	0.00	0.00	0.00	0.00	0.00	0.03	0.04	0.03	0.00	0.00	0.00	0.25	1.80	0.30
Benzeneacetaldehyde, .alpha.-ethylidene-	0.00	0.00	0.00	0.00	0.00	0.00	0.00	0.00	0.00	0.00	0.00	0.00	0.00	0.00	0.00	0.00	0.00	0.00	0.00	0.00	0.00	0.26	0.30	0.26	0.00	0.00	0.00	0.00	0.00	0.00
Butanal, 3-methyl-	0.00	0.00	0.00	0.00	0.00	0.00	0.00	0.00	0.00	0.00	0.00	0.00	0.00	0.00	0.00	0.00	0.00	0.00	0.00	0.00	0.00	0.03	0.06	0.03	0.00	0.00	0.00	0.00	1.40	0.15
Furfural	0.00	0.00	0.00	0.00	0.00	0.00	0.00	0.00	0.00	0.00	0.00	0.00	0.00	0.00	0.00	0.00	0.00	0.00	0.00	0.00	0.00	0.22	0.00	0.00	0.00	0.00	0.00	1.62	0.31	0.00
Nonanal	0.00	0.00	0.00	0.00	0.00	0.00	0.00	0.00	0.00	0.00	0.00	0.00	0.00	0.00	0.00	0.00	0.00	0.00	0.00	0.00	0.00	0.00	0.00	0.00	0.43	0.43	0.00	0.00	0.00	0.00
1,3-Dioxolane,	0.	0.	0.	0.	0.	0.	0.	0.	0.	0.	0.	0.	3.	2.	2.	6.	0.	7.	12	9.	0.	0.	0.	0.	6.	6.	13	0.	0.	0.

2,4,5-trimethyl-	00	00	00	00	00	00	00	00	00	00	00	00	15	79	59	98	09	93	.85	74	13	38	63	38	28	28	.68	84	61	18
2(3H)-Furanone, dihydro-5-pentyl-	0.00	0.00	0.00	0.00	0.00	0.00	0.00	0.00	0.00	0.00	0.03	0.96	2.46	2.18	2.04	2.87	0.04	2.98	4.13	3.15	0.04	0.19	0.41	0.19	1.54	1.54	3.82	1.44	9.86	1.97
2-Methoxy-5-methylphenol	0.00	0.00	0.00	0.00	0.00	0.00	0.00	0.00	0.00	0.02	0.64	0.33	1.45	1.45	1.29	0.00	1.81	0.03	2.35	1.83	0.03	0.00	0.00	0.00	0.90	0.90	2.28	0.00	0.00	0.00
Acetoin	0.00	0.00	0.00	0.00	0.00	0.00	0.00	0.00	0.00	0.00	0.00	0.00	0.00	0.00	0.00	0.00	0.00	0.00	2.64	1.88	0.02	0.31	0.55	0.31	1.80	1.80	4.17	2.87	3.52	3.67
Benzene, 1-methyl-2-propyl-	0.00	0.00	0.00	0.00	0.00	0.00	0.00	0.00	0.00	0.00	0.00	0.00	0.00	0.00	0.00	0.00	0.00	0.00	0.00	0.00	0.00	0.00	0.00	0.00	0.00	0.00	0.00	0.00	0.08	0.23
Cycloheptasiloxane, tetradecamethyl-	0.00	0.00	0.00	0.01	0.03	0.06	0.36	2.04	0.00	0.02	1.07	0.29	1.42	4.16	1.16	1.87	0.03	3.79	2.22	1.95	0.00	0.00	0.00	0.00	0.43	0.43	0.91	0.00	0.00	0.00
Cyclohexasiloxane, dodecamethyl-	0.00	0.00	0.00	0.00	0.02	0.03	0.30	1.02	0.00	0.02	0.58	0.26	1.13	2.51	0.81	1.48	0.01	1.20	1.34	0.01	0.00	0.00	0.00	0.00	1.06	1.06	3.25	0.00	0.00	0.00
Cyclooctasiloxane, hexadecamethyl-	0.00	0.00	0.00	0.02	0.01	0.01	0.00	0.00	0.00	0.00	0.00	0.00	1.40	1.40	1.40	1.09	1.09	1.09	0.00	0.00	0.00	0.00	0.00	0.00	0.00	0.00	0.00	0.00	0.00	0.00
Cyclotetrasiloxane, octamethyl-	0.00	0.00	0.00	0.01	0.01	0.01	0.33	0.37	0.00	0.39	0.23	0.84	0.00	0.00	0.00	0.00	0.00	0.91	1.36	0.86	0.00	0.00	0.00	0.00	0.58	0.58	0.95	0.00	0.00	0.00
Cyclotrisiloxane, hexamethyl-	0.00	0.00	0.00	0.00	0.02	0.02	0.67	0.75	0.00	0.02	0.65	0.41	1.02	1.17	0.88	1.25	0.02	1.13	1.47	1.05	0.01	0.00	0.00	0.00	0.50	0.50	0.89	0.00	0.00	0.00
Ethane, 1,1-diethoxy-	0.00	0.99	0.98	0.02	0.02	0.02	0.27	0.00	0.00	0.00	0.00	0.00	0.00	0.00	0.00	0.00	0.00	0.00	0.00	0.00	0.00	0.00	0.00	0.00	0.00	0.00	0.00	0.00	0.00	0.00
Phenol, 4-ethyl-2-methoxy-	0.00	0.00	0.00	0.00	0.00	0.00	0.00	0.00	0.00	0.00	0.00	0.00	0.00	0.00	0.00	0.00	0.00	0.00	0.00	0.00	0.00	0.00	0.00	0.00	0.00	0.00	0.00	0.05	0.41	0.07

	Phenol, 5-ethenyl-2-methoxy-	0.00	0.00	0.00	0.00	0.00	0.00	0.00	0.00	0.00	0.00	0.00	0.00	0.00	0.00	0.00	0.00	0.00	0.00	0.00	0.00	0.00	0.03	0.03	0.00	0.00	0.00	0.00	0.00	1.46	0.23
	Cyclopentasiloxane, decamethyl-	0.00	0.00	0.00	0.00	0.00	0.00	0.00	0.00	0.00	0.00	0.00	0.00	0.00	0.00	0.00	0.00	0.00	0.00	0.00	0.00	0.00	0.00	0.00	0.00	0.00	0.00	1.07	0.17	0.00	0.00
	1-Butanol, 3-methyl-, acetate	0.00	1.38	0.00	0.00	0.00	0.00	0.00	0.00	0.00	0.00	17.23	15.02	25.73	25.73	25.73	24.39	28.50	48.78	17.98	62.64	56.19	54.18	56.17	0.00	41.07	42.88	34.45	43.29	0.00	0.00
	gamma-Decalactone	0.81	0.00	0.00	0.00	0.00	0.00	0.00	0.00	0.00	0.00	0.00	0.00	0.00	0.00	0.00	0.00	0.00	0.00	5.34	0.00	0.00	0.00	0.00	0.00	8.63	8.63	9.01	7.94	17.19	8.30
	gamma-Nonanolactone	0.00	0.00	0.00	6.54	11.17	4.89	3.27	12.29	13.80	13.00	0.00	0.00	5.67	5.67	5.67	2.04	3.31	3.84	0.00	2.64	7.40	9.81	9.63	9.68	5.48	9.33	0.00	0.00	0.00	0.00
**Direct inoculation**	Hexyl methacrylate	0.00	0.00	0.00	0.00	0.00	0.00	0.00	0.00	0.00	0.00	0.00	0.00	161.71	161.71	161.71	0.00	0.00	80.33	176.47	171.81	0.00	0.00	0.00	0.00	0.00	0.00	0.00	0.00	0.00	0.00
	8-Methylnonanoic acid, ethyl ester	0.00	0.00	0.00	0.66	0.11	0.42	0.26	1.11	0.00	0.00	0.00	0.00	0.00	0.00	0.00	0.00	0.00	0.00	0.00	0.00	0.00	0.00	0.00	0.00	0.00	0.00	0.00	0.00	0.00	0.00
	9-Octadecenoic acid, ethyl ester	3.05	6.25	4.35	2.74	7.09	2.90	1.10	4.43	6.30	0.00	0.00	0.00	0.00	0.00	0.00	0.00	0.00	0.00	0.00	0.00	0.00	0.00	0.00	0.00	0.00	0.00	0.00	0.00	0.00	0.00
	Acetic acid, 2-phenylethyl ester	2.57	6.83	1.73	0.76	1.55	0.59	0.54	2.03	2.80	4.43	4.31	4.76	14.76	14.76	14.76	15.35	23.76	32.87	0.00	0.00	0.00	0.00	0.00	0.00	0.00	0.00	0.00	0.00	0.00	0.00
	Acetic acid, butyl ester	0.00	0.00	0.00	0.00	0.00	0.00	0.00	0.00	0.00	0.00	0.00	0.00	0.00	0.00	0.00	1.01	1.27	2.08	0.99	3.16	3.17	2.30	2.28	2.52	0.00	0.00	0.00	0.00	0.00	0.00
	Acetic acid, hexyl	0.	0.	0.	0.	0.	0.	0.	0.	0.	0.	0.	0.	2.	2.	2.	2.	2.	4.	1.	4.	3.	3.	3.	3.	1.	2.	0.	0.	0.	0.

ester	00	00	00	00	00	00	00	00	00	00	00	00	32	32	32	37	65	86	18	64	96	45	26	11	57	03	00	00	00	00
Benzeneacetic acid, ethyl ester	0.00	0.00	0.00	7.41	10.76	8.39	2.82	7.01	8.90	5.69	6.44	6.60	2.67	2.67	2.67	1.23	1.93	2.48	1.35	3.27	4.44	5.64	5.55	5.57	4.68	3.14	4.67	3.16	6.51	3.96
Benzoic acid, ethyl ester	10.45	13.73	8.77	29.74	53.99	20.90	10.27	34.70	54.02	13.11	14.41	14.49	3.60	3.60	3.60	1.43	1.98	2.22	0.86	2.34	2.71	3.05	3.10	2.97	2.43	2.05	2.53	2.20	4.05	2.96
Butanedioic acid, diethyl ester	0.00	0.00	0.00	0.00	0.00	0.00	0.00	0.00	0.00	16.83	16.86	19.51	12.56	12.56	12.56	3.51	5.64	8.26	2.51	5.58	10.30	9.98	11.55	12.29	9.25	4.39	8.42	6.41	13.35	6.56
Butyrolactone	1.13	1.80	0.64	0.00	0.00	0.22	0.37	0.42	0.53	0.00	0.00	0.00	0.00	0.00	0.00	0.00	0.00	0.00	0.00	0.00	0.00	0.00	0.00	0.00	0.00	0.00	0.00	0.00	0.00	0.00
Decanoic acid, ethyl ester	0.00	0.00	0.22	0.00	0.00	0.00	0.00	0.00	0.00	9.04	8.40	8.36	0.00	0.00	0.00	0.00	1.02	1.58	0.68	1.64	2.42	2.48	2.40	2.74	0.00	0.00	0.00	0.00	0.00	0.00
Diethyl azelate	0.00	0.00	0.00	0.61	1.02	0.53	0.31	1.24	1.42	6.15	4.03	4.51	3.12	3.12	3.12	0.00	0.00	0.00	0.00	0.00	0.00	0.00	0.00	0.00	0.00	0.00	0.00	0.00	0.00	0.00
Dodecanoic acid, ethyl ester	0.00	0.00	0.00	2.01	6.00	2.57	1.65	0.00	0.00	0.00	0.00	0.00	0.00	0.00	0.00	0.00	0.00	0.00	0.00	0.00	0.00	0.00	0.00	0.00	0.00	0.00	0.00	0.00	0.00	0.00
Ethyl Acetate	22.00	36.30	21.17	26.31	44.98	27.63	13.33	38.88	61.13	119.95	99.59	103.96	0.00	0.00	0.00	0.00	1.88	1.02	0.85	1.38	0.00	0.00	0.00	2.82	0.00	0.00	0.00	11.18	17.40	11.14
Heptanoic acid, ethyl ester	0.00	0.00	0.00	0.00	4.06	3.31	4.93	0.00	0.00	8.36	8.46	7.73	0.00	0.00	0.00	0.55	0.87	0.00	0.00	0.00	0.00	0.00	0.00	0.00	0.00	0.00	0.00	0.00	0.00	0.00
Hexadecanoic acid, ethyl ester	66.72	108.21	73.18	49.31	88.32	55.18	14.47	50.65	60.36	36.67	32.63	36.16	10.70	10.70	10.70	2.54	3.59	3.24	1.20	3.67	5.90	6.34	6.31	6.88	3.91	2.20	4.44	2.50	6.57	2.19

Hexanoic acid, 2-methylpropyl ester	0.00	0.00	0.00	0.51	0.83	0.40	0.00	0.00	0.00	0.00	0.00	0.00	0.00	0.00	0.00	0.00	0.00	0.00	0.00	0.00	0.00	0.00	0.00	0.00	0.00	0.00	0.00	0.00	0.00	0.00
Hexanoic acid, ethyl ester	14.16	8.27	8.47	50.52	48.07	30.15	13.03	38.04	43.21	104.34	105.47	97.75	22.29	22.29	22.29	7.35	6.84	11.71	1.22	6.06	4.13	3.32	3.06	2.92	0.00	0.00	2.50	2.80	2.14	3.01
Isobutyl acetate	0.00	0.00	0.00	0.00	0.00	0.00	0.00	0.00	0.00	0.00	0.00	5.04	8.41	8.41	8.41	7.12	8.29	13.41	5.59	16.78	17.69	17.24	17.30	19.11	12.30	12.19	10.98	11.07	12.65	12.00
Linoleic acid ethyl ester	0.00	0.00	0.00	0.00	0.00	0.00	0.00	0.00	0.00	0.00	0.00	0.00	0.00	0.00	0.00	0.53	0.00	0.00	0.00	0.00	0.00	0.00	0.00	0.00	0.00	0.00	0.00	0.00	0.00	0.00
Nonanoic acid, ethyl ester	0.00	0.00	0.00	2.65	3.06	3.54	7.48	13.94	7.21	17.76	11.16	11.69	1.79	1.79	1.79	0.92	1.08	1.21	1.10	1.71	1.91	0.00	0.00	0.00	0.00	0.00	0.00	0.00	0.00	0.00
n-Propyl acetate	0.00	0.00	0.00	0.00	0.00	0.00	0.00	0.00	0.00	0.00	0.00	0.00	0.00	0.00	0.00	0.72	0.95	1.41	0.82	2.05	2.54	2.47	2.55	2.83	0.00	0.00	0.00	0.00	0.00	0.00
Octanoic acid, ethyl ester	15.06	20.62	15.12	74.60	156.77	55.52	20.62	80.72	89.93	62.32	59.64	58.23	6.40	6.40	6.40	3.30	3.13	4.95	1.29	4.35	3.89	0.00	8.16	8.63	3.96	0.00	0.00	4.21	5.04	0.00
Pentadecanoic acid, ethyl ester	0.00	0.00	0.00	0.68	2.31	0.94	1.02	2.02	0.00	0.00	0.00	0.00	0.00	0.00	0.00	0.00	0.00	0.00	0.00	0.00	0.00	0.00	0.00	0.00	0.00	0.00	0.00	0.00	0.00	0.00
Pentanoic acid, ethyl ester	0.00	0.00	0.00	2.55	3.50	1.54	1.22	3.61	5.10	7.35	7.23	6.77	1.85	1.85	1.85	0.53	0.99	0.00	0.00	0.00	0.00	0.00	0.00	0.00	0.00	0.00	0.00	0.00	0.00	0.00
Tetradecanoic acid, ethyl ester	4.13	6.23	5.19	3.41	9.47	4.80	1.99	5.23	4.42	0.00	0.00	0.00	0.00	0.00	0.00	0.00	0.00	0.00	0.00	0.00	0.00	0.00	0.00	0.00	0.00	0.00	0.00	0.00	0.00	0.00
Acetic acid	3.	2.0	3.	3.	5.7	7.	6.	6.	2.4	7.4	7.4	8.5	21.	21.	21.	56.5	60.8	60.6	75.	69.	60.9	71.9	73.2	71.9	84.3	84.3	80.4	101.6	129.2	126.9

	89	2	56	31	2	29	64	25	7	7	7	4	12	12	12	6	4	6	27	01	6	0	7	0	7	7	6	2	0	8
Alanine	0.00	0.00	9.29	1.28	0.76	0.00	0.00	0.00	0.00	0.00	0.00	0.00	0.00	0.00	0.00	0.00	0.00	0.00	0.00	0.00	0.00	0.00	0.00	0.00	0.00	0.00	0.00	0.00	0.00	0.00
Butanoic acid, 3-methyl-	0.00	0.00	0.00	0.00	0.00	0.00	0.00	0.00	0.00	0.00	0.00	0.00	0.00	0.00	0.00	2.71	4.07	6.15	4.40	9.43	13.75	25.58	26.42	27.45	26.55	14.58	24.54	30.76	64.39	28.88
Heptanoic acid	0.00	0.00	0.00	0.00	0.00	0.00	0.00	0.00	0.00	0.00	0.00	0.00	0.00	0.00	0.00	0.00	0.00	0.00	0.00	0.00	0.00	0.00	0.00	2.34	2.51	1.58	2.92	2.15	0.00	0.00
Hexanoic acid	0.00	0.00	0.00	0.00	0.00	0.00	0.00	0.00	0.00	11.76	14.00	14.26	6.82	6.82	6.82	0.00	5.51	6.51	4.74	15.15	0.00	24.72	24.03	23.40	26.56	17.05	28.39	29.06	59.97	27.90
Lactic acid	0.00	0.00	3.56	7.18	0.00	0.00	3.15	2.06	0.00	0.00	0.00	0.00	0.00	0.00	0.00	0.00	0.00	0.00	0.00	0.00	0.00	0.00	0.00	0.00	0.00	0.00	0.00	0.00	0.00	0.00
Octanoic acid	0.00	0.00	0.00	0.00	0.00	0.00	0.00	0.00	0.00	4.63	5.54	6.21	1.70	1.70	1.70	0.63	1.06	1.09	1.19	2.33	2.91	4.77	5.19	4.92	5.74	3.61	6.39	4.99	5.81	0.00
Propanoic acid, 2-methyl-	0.00	0.00	0.00	0.00	0.00	0.00	0.00	0.00	0.00	0.00	0.00	0.00	0.00	0.00	0.00	0.00	0.00	0.00	1.00	1.45	2.15	3.08	3.87	0.00	0.00	0.00	0.00	0.00	0.00	0.00
10-Undecen-1-ol	0.00	0.00	0.00	0.00	0.00	0.00	0.00	0.00	0.00	14.80	14.80	15.00	0.00	0.00	0.00	0.00	0.00	0.00	0.00	0.00	0.00	0.00	0.00	0.00	0.00	0.00	0.00	0.00	0.00	0.00
(Z)-4-Decen-1-ol	0.00	0.00	0.00	0.00	0.00	0.00	0.00	0.00	0.00	13.15	5.78	5.98	3.47	3.47	3.47	0.00	0.00	0.00	0.00	0.00	0.00	0.00	0.00	0.00	0.00	0.00	0.00	0.00	0.00	0.00
alpha-Terpineol	0.00	0.00	0.00	0.00	2.72	0.99	0.44	1.33	1.58	0.00	0.00	0.00	0.00	0.00	0.00	0.00	0.00	0.00	0.00	0.00	0.00	0.00	0.00	0.00	0.00	0.00	0.00	0.00	0.00	0.00

Benzeneethanol, b-ethyl-	0.00	0.00	0.00	0.00	0.00	0.00	0.00	0.00	0.00	0.00	0.00	0.00	0.00	0.00	0.00	0.00	0.00	0.00	0.52	1.10	1.54	2.02	2.01	0.00	1.86	1.17	1.97	0.00	1.88	1.88
1-Butanol, 3-methyl-	99.56	137.55	82.64	87.76	94.84	54.49	30.36	85.97	108.84	222.57	253.36	224.32	78.45	78.45	78.45	15.84	25.28	37.95	10.77	29.11	33.40	33.10	38.04	35.26	21.98	12.72	25.19	15.26	13.30	14.30
1-Heptanol	0.00	0.00	0.00	0.00	0.00	0.00	0.00	0.00	0.00	0.00	5.99	7.98	2.08	2.08	2.08	0.00	0.00	0.00	0.00	0.00	0.00	0.00	0.00	0.00	0.00	0.00	0.00	0.00	0.00	0.00
1-Hexanol	2.22	1.32	0.00	17.55	9.89	5.26	5.75	16.07	20.42	38.45	39.88	42.82	12.25	12.25	12.25	3.18	4.64	6.40	1.71	4.01	5.27	4.15	3.92	4.06	2.32	1.39	2.10	0.00	0.00	0.00
1-Nonanol	0.00	0.00	0.00	0.29	0.99	0.35	0.33	0.68	1.75	5.43	5.70	6.00	0.00	0.00	0.00	0.54	0.56	0.80	0.00	0.00	0.00	0.00	0.00	0.00	0.00	0.00	0.00	0.00	0.00	0.00
1-Octanol	0.00	0.00	0.00	0.00	0.00	0.00	0.00	0.00	0.00	6.73	7.13	7.23	1.98	1.98	1.98	0.00	0.00	0.83	0.00	0.00	0.00	0.00	0.00	0.00	0.00	0.00	0.00	0.00	0.00	0.00
1-Propanol, 2-methyl-	23.21	43.64	22.59	24.07	24.75	15.67	7.59	20.61	26.13	81.16	72.64	77.30	20.68	20.68	20.68	5.49	8.10	11.78	3.25	6.86	10.24	9.93	9.74	11.03	6.58	3.80	6.07	3.97	7.98	3.83
2,3-Butanediol	0.00	6.40	24.33	0.81	3.28	4.78	0.00	0.00	0.00	0.00	0.00	0.00	0.00	0.00	0.00	0.00	0.00	0.00	0.00	0.00	0.00	0.00	0.00	0.00	0.00	0.00	0.00	0.00	0.00	0.00
2,3-Butanediol, [S-(R*,R*)]-	0.00	0.00	0.00	0.00	0.00	0.00	0.00	0.00	0.00	11.20	4.18	4.62	0.00	0.00	0.00	1.44	1.73	3.38	0.66	2.57	3.05	5.60	3.85	7.06	4.33	1.31	4.78	7.54	8.11	9.98
Isopropyl Alcohol	2.83	4.41	0.39	0.44	2.18	5.17	0.00	0.00	0.00	0.00	0.00	0.00	0.00	0.00	0.00	0.00	0.00	0.00	0.00	0.00	0.00	0.00	0.00	0.00	0.00	0.00	0.00	0.00	0.00	0.00

Ethanol	0.00	0.00	0.00	0.00	0.00	0.00	0.00	0.00	0.00	8.90	8.73	0.00	0.00	0.00	0.00	39.19	55.67	82.67	19.50	39.77	59.62	32.42	32.95	37.27	8.53	4.75	7.75	0.00	0.00	0.00
Benzaldehyde	0.00	0.00	0.00	2.74	5.18	2.19	3.11	5.08	12.28	0.00	0.00	0.00	0.00	0.00	0.00	0.00	0.00	0.00	0.00	0.00	0.00	0.00	0.00	0.00	1.73	1.05	1.71	0.00	0.00	0.00
Benzeneacetaldehyde	1.54	2.03	0.92	2.71	5.10	1.21	1.47	4.50	9.24	4.69	4.58	5.18	2.05	2.05	2.05	0.81	1.33	1.49	0.61	1.42	1.70	1.55	0.95	0.00	0.00	0.00	0.00	0.00	0.00	0.00
Benzeneacetaldehyde, .alpha.-ethylidene-	0.00	0.00	0.00	0.00	0.00	0.00	0.28	0.92	1.43	0.00	0.00	0.00	0.00	0.00	0.00	0.00	0.00	0.00	1.27	2.75	4.12	8.52	8.65	9.12	4.71	3.08	4.98	0.00	0.00	0.00
Butanal, 3-methyl-	0.00	0.93	0.65	1.65	2.87	1.94	0.96	2.40	3.23	0.00	0.00	0.00	0.00	0.00	0.00	0.00	0.00	0.00	0.00	0.00	0.00	0.00	0.00	0.00	0.00	0.00	0.00	0.00	0.00	0.00
Nonanal	0.00	0.00	0.00	0.79	1.54	0.34	0.00	0.00	0.00	5.45	0.00	0.00	0.00	0.00	0.00	0.00	0.00	0.00	0.00	0.00	1.03	0.00	0.00	0.00	0.98	0.00	0.00	0.00	0.00	0.00
Propanal, 2-methyl-	0.45	0.81	0.40	0.25	0.13	0.00	0.17	0.46	0.65	0.00	0.00	0.00	0.00	0.00	0.00	0.00	0.00	0.00	0.00	0.00	0.00	0.00	0.00	0.00	0.00	0.00	0.00	0.00	0.00	0.00
Furfural	0.00	0.00	0.00	0.00	0.00	0.00	0.00	0.00	0.00	0.00	0.00	0.00	0.00	0.00	0.00	0.00	0.00	0.00	0.00	0.00	0.00	3.67	3.90	4.38	3.68	2.17	3.54	2.44	5.64	2.62
(2-Aziridinylethyl)amine	10.43	9.26	5.19	10.59	6.84	0.00	2.77	1.68	7.49	0.00	0.00	0.00	0.00	0.00	0.00	0.00	0.00	0.00	0.00	0.00	0.00	0.00	0.00	0.00	0.00	0.00	0.00	0.00	0.00	0.00
Cyclohexasiloxane, dodecamethyl-	0.00	0.00	0.00	0.00	0.00	0.00	0.00	0.00	0.00	0.00	39.03	23.65	16.26	16.26	16.26	0.60	1.80	2.12	4.96	2.44	7.92	3.13	8.99	4.92	5.16	0.92	5.91	2.02	4.16	3.16
Cyclotetrasiloxane,	0.	0.	0.	0.	0.	0.	0.	0.	0.	0.	0.	0.	1.	1.	1.	0.	0.	0.	0.	1.	1.	0.	0.	0.	0.	0.	0.	0.	0.	0.

octamethyl-	00	00	00	00	00	00	00	00	00	00	00	00	66	66	66	00	61	86	55	14	76	00	00	00	00	00	00	00	00	00
Cyclotrisiloxane, hexamethyl-	0.00	0.00	0.00	0.00	0.00	0.00	0.00	0.00	0.00	11.90	10.09	10.71	3.13	3.13	3.13	1.16	1.73	1.94	0.83	1.85	2.53	3.06	2.71	3.45	2.38	1.38	2.09	2.02	4.47	1.92
Acetoin	2.12	1.56	3.07	0.57	1.07	4.06	0.20	0.00	0.00	0.00	0.00	0.00	0.00	0.00	0.00	0.00	0.00	0.00	0.00	0.00	0.00	3.40	3.50	4.11	3.30	1.91	3.04	4.23	9.12	4.29
2-Methoxy-4-vinylphenol	0.00	0.00	0.00	0.00	0.00	0.00	0.00	0.00	0.00	0.00	0.00	0.00	0.00	0.00	0.00	0.67	1.15	0.98	1.19	2.41	3.56	5.79	5.95	5.92	5.06	3.23	5.54	3.71	7.69	3.61
1,3-Dioxolane, 2,4,5-trimethyl-	0.00	0.00	0.00	0.00	0.00	0.00	0.00	0.00	0.00	0.00	0.00	0.00	0.00	0.00	0.00	0.00	0.87	1.02	1.33	3.42	4.18	12.07	13.87	14.34	6.59	4.34	5.95	2.24	2.10	0.00
Azulene	0.00	0.24	0.51	0.75	0.00	0.00	0.00	0.00	1.33	0.00	0.00	0.00	0.00	0.00	0.00	0.00	0.00	0.00	0.00	0.00	0.00	0.00	0.00	0.00	0.00	0.00	0.00	0.00	0.00	0.00
Cycloheptasiloxane, tetradecamethyl-	0.00	0.00	0.00	0.00	0.00	0.00	0.00	0.00	0.00	10.50	23.06	16.93	9.46	9.46	9.46	0.00	2.17	2.76	3.01	1.77	4.68	5.24	6.21	0.00	0.00	6.48	5.88	3.78	7.52	1.95
Cyclopentasiloxane, decamethyl-	0.00	0.00	0.00	0.00	0.00	0.00	0.00	0.00	0.00	0.00	0.00	5.38	1.99	1.99	1.99	0.00	0.00	0.86	0.53	0.00	0.00	0.00	0.00	0.00	0.00	0.00	0.00	0.00	0.00	0.00
Ethyl 4-acetoxybutanoate	0.00	0.00	0.00	0.00	0.00	0.00	0.00	0.00	0.00	0.00	0.00	0.00	0.00	0.00	0.00	1.43	2.30	2.36	2.11	4.41	6.66	7.72	7.93	8.38	5.40	2.86	4.96	3.34	6.97	3.26
Methane, isocyanato-	0.00	0.00	0.00	0.00	0.00	0.00	0.00	0.00	0.00	0.00	0.00	0.00	0.00	0.00	0.00	5.03	7.43	11.07	3.15	7.01	0.00	0.00	9.93	10.42	7.23	4.24	6.73	4.74	9.27	4.54

Supplementary Table 5. Analysis of microbial diversity using different inoculation strategies during fermentation.

Fermentation Days(d)	16S-Chao1		16S-Shannon		ITS-Chao1		ITS-Shannon	
	Traditional inoculation	Direct inoculation	Traditional inoculation	Direct inoculation	Traditional inoculation	Direct inoculation	Traditional inoculation	Direct inoculation
	308.00	267.15	3.03	2.35	295.24	174.17	2.14	1.72
0	278.14	332.66	2.90	3.41	235.67	224.04	2.04	1.85
	337.26	390.93	2.58	3.20	244.55	261.24	1.94	2.17
	406.25	289.33	4.34	3.48	217.14	183.38	2.28	2.03
2	375.04	292.91	4.41	3.87	251.16	169.78	2.34	2.12
	362.37	275.00	4.14	3.38	214.14	177.20	2.03	2.04
	327.12	254.25	3.28	1.90	244.89	177.00	2.46	1.97
4	264.05	233.28	3.73	3.13	222.89	185.11	2.03	2.06
	300.00	265.89	3.51	2.49	209.00	178.35	2.12	2.20
	248.15	121.75	3.42	0.63	118.06	166.81	1.68	2.14
6	273.72	168.67	3.28	0.69	143.60	196.48	0.85	2.20
	311.22	135.00	3.46	0.63	206.33	234.17	1.81	2.35
8	263.79	102.00	2.58	0.73	140.38	148.88	1.31	1.99

	226.33	85.77	2.49	0.61	143.65	188.75	0.94	2.12
	202.07	93.09	2.67	0.67	136.09	156.87	0.96	2.28
	183.00	96.00	1.58	1.67	99.08	139.46	1.27	2.36
10	158.47	99.40	1.84	0.67	92.09	147.11	1.28	2.20
	145.47	126.47	1.58	0.64	85.00	194.63	1.20	2.25
	199.94	108.50	0.67	1.01	88.60	190.75	1.39	2.91
12	155.59	67.75	0.87	0.65	113.20	123.23	1.06	2.28
	221.44	85.60	0.84	0.84	115.46	117.25	1.77	2.29
	190.30	66.00	0.42	0.85	108.50	111.25	2.11	2.11
14	137.00	108.00	0.28	0.74	103.38	140.33	1.61	2.27
	142.13	78.50	0.33	1.60	128.27	128.00	1.87	2.58
	109.83	139.60	0.19	0.88	94.43	117.11	1.57	2.54
16	122.91	161.15	0.10	0.94	83.75	210.09	2.29	2.71
	117.55	147.14	0.12	2.36	78.00	107.08	1.03	2.88
	115.63	103.87	0.08	0.98	83.50	130.25	1.59	1.87
18	107.36	122.37	0.32	1.12	59.20	154.71	1.35	2.51
	111.33	107.88	0.19	1.08	66.50	103.77	1.18	2.95

Compare	Direct inoculation-vs-Tradition inoculation			
Method	T-test			
p.format	0.01	0.24	0.23	2.70E-07
p.signif	*	ns	ns	****

Supplementary Table 6. Change of bacterial genus level under traditional inoculation method(T) and direct inoculation method9(D) (relative abundance>1%).

Fermentation Days	Acetobacter	Lactobacillus	Agrobacterium	Sphingomonas	Xanthomonas	Pantoea	Methylobacterium	Stenotrophomonas	Curtobacterium	Novosphingobium	Others	Unassigned
T0-1	0.33%	1.37%	3.95%	1.64%	2.14%	1.97%	1.40%	0.23%	0.82%	0.31%	2.22%	83.62%
T0-2	0.85%	5.87%	8.77%	4.73%	5.32%	3.32%	5.03%	0.54%	3.05%	1.22%	7.35%	53.95%
T0-3	0.86%	5.02%	5.96%	3.81%	4.42%	2.53%	3.76%	0.38%	3.47%	1.89%	7.64%	60.25%
T2-1	1.92%	5.81%	15.97%	6.17%	5.57%	3.00%	4.47%	1.62%	2.72%	0.97%	6.72%	45.04%
T2-2	2.72%	5.95%	16.34%	6.15%	7.60%	3.17%	7.11%	0.87%	2.61%	1.25%	9.23%	37.00%
T2-3	2.07%	6.63%	16.18%	4.22%	6.00%	4.64%	3.95%	0.94%	1.88%	1.16%	6.74%	45.60%
T4-1	19.20%	65.66%	3.44%	1.32%	0.80%	2.81%	0.75%	0.27%	0.76%	0.24%	1.74%	3.01%
T4-2	42.75%	18.01%	10.95%	6.07%	0.39%	0.24%	3.13%	0.07%	2.34%	0.70%	4.05%	11.30%

T4-3	18.55%	56.30%	6.23%	2.58%	1.96%	1.55%	1.41%	0.53%	1.67%	0.35%	3.02%	5.84%
T6-1	9.50%	89.28%	0.29%	0.15%	0.15%	0.16%	0.05%	0.03%	0.06%	0.03%	0.19%	0.12%
T6-2	9.95%	88.58%	0.28%	0.17%	0.13%	0.17%	0.05%	0.04%	0.07%	0.03%	0.30%	0.22%
T6-3	7.84%	90.37%	0.48%	0.12%	0.12%	0.29%	0.07%	0.06%	0.17%	0.02%	0.18%	0.26%
T8-1	14.03%	84.93%	0.20%	0.11%	0.11%	0.16%	0.04%	0.04%	0.09%	0.03%	0.17%	0.08%
T8-2	10.54%	88.64%	0.17%	0.07%	0.10%	0.18%	0.01%	0.01%	0.04%	0.01%	0.11%	0.12%
T8-3	11.96%	87.14%	0.14%	0.12%	0.11%	0.14%	0.03%	0.02%	0.07%	0.03%	0.13%	0.11%
T10-1	51.06%	46.69%	0.63%	0.43%	0.10%	0.12%	0.08%	0.02%	0.14%	0.05%	0.26%	0.42%
T10-2	13.77%	85.62%	0.13%	0.07%	0.06%	0.06%	0.01%	0.01%	0.04%	0.03%	0.10%	0.10%
T10-3	11.44%	87.65%	0.22%	0.11%	0.08%	0.15%	0.02%	0.01%	0.04%	0.02%	0.16%	0.09%
T12-1	17.48%	79.68%	0.09%	0.08%	0.05%	0.04%	0.00%	0.06%	0.02%	0.01%	1.24%	1.26%
T12-2	12.53%	86.67%	0.19%	0.06%	0.09%	0.08%	0.01%	0.02%	0.04%	0.01%	0.20%	0.10%
T12-3	19.22%	79.76%	0.15%	0.12%	0.08%	0.15%	0.01%	0.12%	0.09%	0.02%	0.17%	0.09%
T14-1	18.49%	80.18%	0.44%	0.16%	0.05%	0.05%	0.00%	0.02%	0.07%	0.03%	0.36%	0.15%
T14-2	16.98%	82.55%	0.06%	0.06%	0.04%	0.02%	0.00%	0.04%	0.02%	0.00%	0.15%	0.06%
T14-3	52.05%	46.27%	0.37%	0.44%	0.01%	0.03%	0.03%	0.00%	0.05%	0.04%	0.33%	0.37%
T16-1	19.50%	79.20%	0.13%	0.12%	0.04%	0.03%	0.02%	0.01%	0.04%	0.01%	0.61%	0.30%
T16-2	23.18%	75.58%	0.17%	0.10%	0.04%	0.04%	0.01%	0.02%	0.03%	0.01%	0.55%	0.27%

T16-3	15.59%	62.88%	0.25%	1.32%	0.03%	0.18%	0.02%	0.04%	0.00%	0.07%	14.99 %	4.64%
T18-1	18.01%	79.18%	0.19%	0.15%	0.01%	0.07%	0.01%	0.02%	0.07%	0.00%	1.91%	0.37%
T18-2	28.36%	68.79%	0.13%	0.07%	0.06%	0.06%	0.03%	0.02%	0.02%	0.09%	1.96%	0.40%
T18-3	17.74%	77.31%	0.04%	0.07%	0.02%	0.01%	0.02%	0.01%	0.00%	0.01%	4.43%	0.34%
D0-1	1.47%	0.37%	10.86%	6.63%	5.55%	3.03%	5.01%	0.72%	1.71%	1.49%	5.41%	57.76%
D0-2	1.93%	0.89%	13.28%	8.29%	0.46%	0.35%	7.08%	0.05%	1.79%	2.27%	4.19%	59.41%
D0-3	0.97%	0.03%	4.76%	3.92%	2.99%	1.97%	1.82%	0.51%	0.77%	0.87%	3.85%	77.54%
D2-1	11.41%	0.06%	18.20%	16.18%	11.91%	6.78%	4.20%	1.86%	4.32%	3.94%	10.56 %	10.57%
D2-2	5.91%	0.21%	18.83%	13.49%	10.71%	8.75%	5.69%	2.29%	3.55%	3.58%	11.01 %	15.98%
D2-3	7.18%	0.04%	19.54%	13.14%	11.45%	9.81%	3.61%	2.77%	2.48%	2.46%	10.14 %	17.39%
D4-1	37.92%	0.03%	20.30%	11.16%	7.86%	8.14%	1.77%	1.55%	1.23%	1.62%	5.64%	2.79%
D4-2	24.31%	0.03%	23.08%	11.98%	9.37%	8.32%	2.07%	1.75%	2.97%	2.65%	9.02%	4.45%
D4-3	34.17%	0.04%	20.63%	10.23%	7.73%	7.27%	2.28%	1.41%	2.77%	1.75%	8.53%	3.20%
D6-1	34.01%	0.11%	23.53%	16.13%	0.79%	0.45%	1.85%	0.19%	1.34%	2.46%	13.17 %	5.99%
D6-2	23.98%	0.11%	19.28%	5.10%	3.26%	6.69%	1.10%	26.84%	1.58%	1.50%	7.05%	3.51%
D6-3	30.73%	0.68%	28.56%	9.54%	4.62%	6.79%	1.14%	2.65%	2.58%	2.22%	8.12%	2.38%

D8-1	40.91%	39.85%	6.54%	3.66%	1.36%	2.27%	0.18%	0.68%	0.38%	0.82%	2.52%	0.85%
D8-2	29.06%	51.67%	7.18%	3.40%	1.34%	2.87%	0.18%	0.52%	0.34%	0.49%	1.71%	1.25%
D8-3	40.79%	35.60%	7.88%	5.59%	2.92%	1.93%	0.24%	0.95%	0.36%	0.48%	2.40%	0.87%
D10-1	72.10%	19.57%	2.65%	1.66%	0.77%	0.96%	0.03%	0.31%	0.20%	0.22%	1.20%	0.34%
D10-2	56.50%	36.49%	2.49%	1.39%	0.51%	0.60%	0.03%	0.48%	0.09%	0.28%	0.84%	0.31%
D10-3	68.12%	21.15%	5.17%	2.87%	0.11%	0.17%	0.05%	0.26%	0.21%	0.60%	0.97%	0.30%
D12-1	93.24%	1.57%	1.32%	1.00%	0.62%	0.39%	0.02%	0.16%	0.05%	0.23%	1.13%	0.26%
D12-2	90.21%	4.49%	1.13%	1.15%	0.71%	0.48%	0.05%	0.18%	0.06%	0.42%	0.99%	0.14%
D12-3	91.90%	2.52%	0.76%	0.54%	0.57%	0.34%	0.02%	0.15%	0.04%	0.15%	2.55%	0.46%
D14-1	96.30%	1.21%	0.27%	0.15%	0.15%	0.12%	0.01%	0.04%	0.01%	0.07%	1.62%	0.06%
D14-2	97.59%	0.57%	0.39%	0.27%	0.21%	0.12%	0.01%	0.06%	0.03%	0.08%	0.54%	0.14%
D14-3	97.17%	0.94%	0.29%	0.15%	0.15%	0.11%	0.01%	0.06%	0.01%	0.09%	0.89%	0.14%
D16-1	98.30%	0.93%	0.14%	0.07%	0.06%	0.04%	0.00%	0.03%	0.01%	0.02%	0.34%	0.05%
D16-2	99.28%	0.24%	0.12%	0.07%	0.01%	0.01%	0.01%	0.00%	0.02%	0.04%	0.15%	0.06%
D16-3	99.09%	0.44%	0.05%	0.03%	0.02%	0.02%	0.00%	0.01%	0.00%	0.01%	0.28%	0.04%
D18-1	99.40%	0.29%	0.04%	0.02%	0.01%	0.01%	0.00%	0.00%	0.00%	0.01%	0.18%	0.04%
D18-2	96.71%	2.01%	0.04%	0.03%	0.02%	0.00%	0.01%	0.01%	0.00%	0.01%	0.77%	0.38%
D18-3	98.12%	1.24%	0.09%	0.03%	0.03%	0.02%	0.01%	0.01%	0.00%	0.00%	0.40%	0.04%

Supplementary Table 7. Changes of fungal genus level under traditional inoculation and direct inoculation (relative abundance>1%).

Fermentation Days	Saccharomyces	Alternaria	Aspergillus	Neosetophoma	Moesziomyces	Ustilaginoidea	Sarocladium	Rhizopus	Meyerozyma	Cladosporium	Others	Unclassified	Unassigned
T0-1	2.72%	3.73%	0.10%	0.18%	0.04%	0.01%	0.01%	0.02%	0.00%	0.00%	0.20%	0.01%	92.99%
T0-2	3.00%	4.65%	0.13%	0.32%	0.10%	0.03%	0.06%	0.04%	0.00%	0.00%	0.16%	0.01%	91.50%
T0-3	3.98%	7.88%	0.25%	0.62%	0.08%	0.03%	0.03%	0.04%	0.00%	0.01%	0.23%	0.05%	86.80%
T2-1	19.00%	0.62%	0.05%	0.04%	0.03%	0.01%	0.00%	0.11%	0.00%	0.00%	0.04%	0.00%	80.09%
T2-2	19.56%	0.86%	0.05%	0.06%	0.02%	0.02%	0.00%	0.03%	0.00%	0.00%	0.04%	0.00%	79.35%
T2-3	23.25%	0.61%	0.08%	0.06%	0.05%	0.02%	0.00%	0.06%	0.00%	0.00%	0.06%	0.00%	75.82%
T4-1	47.23%	2.38%	0.23%	0.14%	0.17%	0.12%	0.01%	0.07%	0.00%	0.10%	0.13%	0.04%	49.38%
T4-2	35.31%	1.67%	0.12%	0.10%	0.17%	0.06%	0.00%	0.08%	0.00%	0.11%	0.10%	0.02%	62.26%
T4-3	28.05%	0.60%	0.06%	0.13%	0.13%	0.02%	0.02%	0.04%	0.00%	0.00%	0.12%	0.03%	70.80%
T6-1	28.45%	0.49%	0.03%	0.05%	0.13%	0.02%	0.01%	0.05%	0.00%	0.00%	0.09%	0.00%	70.69%
T6-2	51.38%	2.74%	0.19%	1.10%	0.83%	0.18%	0.06%	0.10%	0.00%	0.00%	0.49%	0.08%	42.86%
T6-3	40.86%	1.08%	0.08%	0.44%	0.89%	0.05%	0.01%	0.07%	0.00%	0.00%	0.08%	0.01%	56.41%
T8-1	50.31%	0.89%	0.08%	0.15%	0.24%	0.06%	0.00%	0.13%	0.00%	0.02%	0.12%	0.00%	48.01%
T8-2	43.10%	1.19%	0.09%	0.57%	0.24%	0.06%	0.00%	0.12%	0.00%	0.02%	0.16%	0.09%	54.37%

T8-3	43.10%	1.19%	0.09%	0.57%	0.24%	0.06%	0.00%	0.12%	0.00%	0.02%	0.16%	0.09%	54.37%
T10-1	40.20%	2.91%	0.27%	0.29%	0.40%	0.18%	0.00%	0.08%	0.00%	0.22%	0.47%	0.10%	54.87%
T10-2	40.78%	1.49%	0.26%	1.08%	0.21%	0.19%	0.00%	0.21%	0.00%	0.00%	0.19%	0.13%	55.46%
T10-3	25.05%	0.78%	0.07%	0.22%	0.16%	0.13%	0.03%	0.06%	0.00%	0.00%	0.07%	0.04%	73.40%
T12-1	50.28%	6.67%	0.17%	2.55%	1.32%	6.11%	0.00%	0.57%	0.00%	0.00%	0.72%	0.00%	31.59%
T12-2	51.39%	2.71%	0.12%	0.54%	0.03%	0.07%	0.11%	0.25%	0.00%	0.00%	0.07%	0.00%	44.71%
T12-3	44.46%	1.92%	0.53%	0.09%	0.47%	0.68%	0.08%	0.00%	0.00%	0.01%	0.19%	0.00%	51.56%
T14-1	62.32%	1.97%	0.51%	0.00%	0.51%	0.59%	0.00%	0.00%	0.00%	0.06%	1.83%	0.00%	32.20%
T14-2	54.14%	3.31%	0.33%	0.21%	0.35%	0.19%	0.00%	0.00%	0.00%	0.36%	0.70%	0.43%	39.98%
T14-3	50.89%	2.79%	1.13%	1.31%	0.49%	0.33%	0.19%	0.58%	0.00%	0.74%	0.01%	0.30%	41.24%
T16-1	49.67%	4.71%	0.21%	0.00%	0.56%	0.00%	0.00%	0.42%	0.00%	0.04%	2.75%	0.55%	41.07%
T16-2	32.18%	4.42%	0.62%	0.23%	0.67%	0.28%	0.14%	0.07%	0.00%	0.00%	0.42%	0.06%	60.92%
T16-3	47.72%	5.98%	1.23%	0.28%	0.34%	0.00%	1.01%	1.82%	0.00%	0.03%	2.12%	0.00%	39.48%
T18-1	65.33%	3.20%	0.06%	0.00%	0.00%	0.00%	0.00%	0.00%	0.00%	0.02%	0.42%	0.00%	30.96%
T18-2	33.65%	1.93%	0.19%	0.18%	0.15%	0.09%	0.34%	0.07%	0.00%	0.01%	1.44%	0.08%	61.87%
T18-3	43.61%	1.46%	1.53%	0.00%	0.00%	0.00%	1.10%	0.00%	0.00%	1.63%	2.44%	1.85%	46.37%
D0-1	4.45%	5.90%	0.42%	0.30%	0.13%	0.09%	0.04%	0.13%	0.00%	0.23%	0.47%	0.02%	87.83%
D0-2	4.92%	5.04%	0.26%	0.47%	0.10%	0.05%	0.03%	0.09%	0.00%	0.21%	0.23%	0.02%	88.59%

D0-3	2.90%	4.38%	0.28%	0.49%	0.11%	0.05%	0.03%	0.07%	0.00%	0.01%	0.20%	0.01%	91.46%
D2-1	25.68%	1.88%	0.23%	0.21%	0.38%	0.39%	0.02%	0.04%	0.01%	0.00%	0.15%	0.02%	71.00%
D2-2	27.38%	3.64%	0.46%	0.35%	0.39%	0.48%	0.02%	0.04%	0.01%	0.00%	0.17%	0.00%	67.06%
D2-3	37.60%	1.24%	0.21%	0.05%	0.18%	0.08%	0.01%	0.11%	0.01%	0.00%	0.09%	0.01%	60.40%
D4-1	52.62%	1.94%	0.51%	1.62%	0.65%	0.28%	0.01%	0.06%	0.03%	0.00%	0.16%	0.02%	42.10%
D4-2	63.43%	1.38%	0.33%	0.26%	0.52%	0.16%	0.01%	0.08%	0.01%	0.01%	0.22%	0.02%	33.57%
D4-3	66.05%	2.43%	0.72%	3.50%	3.14%	0.25%	0.08%	0.05%	0.02%	0.62%	0.81%	0.05%	22.27%
D6-1	68.81%	2.69%	0.62%	0.11%	0.77%	0.40%	0.06%	0.11%	0.02%	0.24%	0.57%	0.04%	25.56%
D6-2	89.90%	1.60%	1.27%	0.35%	0.77%	0.43%	0.03%	0.43%	0.01%	0.00%	0.09%	0.01%	5.12%
D6-3	62.68%	2.01%	1.42%	0.24%	0.41%	0.24%	0.13%	0.06%	0.00%	0.01%	0.15%	0.03%	32.64%
D8-1	84.36%	2.93%	2.26%	0.79%	0.87%	0.31%	0.05%	0.11%	0.17%	0.01%	0.30%	0.14%	7.72%
D8-2	89.03%	1.73%	1.33%	0.11%	0.60%	0.28%	0.02%	0.06%	0.10%	0.00%	0.18%	0.01%	6.54%
D8-3	87.13%	1.14%	0.85%	0.26%	0.38%	0.24%	0.01%	0.02%	0.08%	0.00%	0.13%	0.00%	9.76%
D10-1	81.88%	1.57%	0.64%	0.15%	0.45%	0.46%	0.00%	0.05%	0.00%	0.01%	0.22%	0.00%	14.57%
D10-2	79.49%	1.59%	1.48%	0.34%	0.53%	0.49%	0.00%	0.08%	0.00%	0.33%	0.27%	0.04%	15.36%
D10-3	81.98%	1.64%	1.31%	0.21%	0.79%	0.35%	0.20%	0.00%	0.00%	0.31%	0.44%	0.10%	12.67%
D12-1	81.76%	1.81%	4.88%	0.21%	1.74%	0.45%	3.87%	0.11%	0.00%	0.01%	0.41%	0.00%	4.76%
D12-2	87.30%	2.17%	1.39%	0.01%	0.28%	0.86%	0.84%	0.00%	0.00%	0.02%	0.14%	0.02%	6.98%

D12-3	75.33%	5.59%	7.42%	2.15%	1.44%	0.52%	0.41%	0.09%	0.01%	0.02%	1.48%	0.29%	5.26%
D14-1	48.63%	3.89%	3.50%	5.45%	0.21%	0.17%	0.20%	0.00%	0.62%	0.00%	0.07%	0.00%	37.24%
D14-2	76.97%	2.55%	5.27%	0.43%	0.46%	0.08%	0.00%	0.19%	0.00%	0.00%	0.66%	0.43%	12.97%
D14-3	71.95%	1.52%	6.54%	0.37%	0.42%	0.15%	0.00%	0.00%	0.11%	0.00%	1.20%	0.00%	17.74%
D16-1	75.59%	2.39%	4.23%	0.00%	0.80%	0.01%	0.17%	0.00%	1.00%	0.00%	0.65%	0.00%	15.16%
D16-2	66.10%	2.21%	2.73%	0.01%	0.21%	1.49%	3.16%	1.07%	0.41%	0.00%	3.34%	0.27%	19.01%
D16-3	87.65%	1.53%	4.48%	0.00%	0.00%	0.17%	0.00%	0.72%	0.23%	0.04%	0.90%	0.90%	3.38%
D18-1	79.53%	2.02%	2.67%	1.59%	0.58%	0.00%	2.98%	0.00%	1.94%	0.11%	2.58%	0.00%	5.98%
D18-2	77.60%	11.10%	0.12%	0.00%	0.00%	0.00%	0.00%	1.06%	1.36%	0.01%	2.56%	0.00%	6.18%
D18-3	84.62%	0.73%	4.80%	0.00%	0.00%	0.31%	0.00%	0.00%	0.00%	0.00%	0.07%	0.00%	9.47%

Supplementary Table 8. Changes in the level of bacteria phyla under traditional inoculation (T) and direct inoculation (D) (relative abundance>1%).

Fermentation Days	Proteobacteria	Firmicutes	Actinobacteria	Bacteroidetes	Actinobacteriota	Gemmatimonadetes	Chloroflexi	Verrucomicrobia	Planctomycetes	Armatimonadetes	Others	Unassigned
T0-1	13.37%	1.83%	1.02%	0.11%	0.02%	0.00%	0.00%	0.00%	0.01%	0.00%	0.01%	83.62%
T0-2	33.51%	7.96%	3.69%	0.68%	0.17%	0.00%	0.00%	0.00%	0.03%	0.00%	0.01%	53.95%
T0-3	27.52%	6.88%	4.07%	0.86%	0.24%	0.00%	0.00%	0.01%	0.11%	0.01%	0.04%	60.25%
T2-1	44.09%	6.42%	3.76%	0.24%	0.36%	0.00%	0.00%	0.00%	0.05%	0.00%	0.02%	45.04%

T2-2	52.54%	6.64%	3.22%	0.30%	0.23%	0.00%	0.00%	0.01%	0.04%	0.00%	0.01%	37.00%
T2-3	43.51%	7.29%	2.51%	0.90%	0.15%	0.00%	0.00%	0.00%	0.01%	0.01%	0.03%	45.60%
T4-1	30.10%	65.91%	0.85%	0.07%	0.04%	0.00%	0.00%	0.00%	0.01%	0.00%	0.01%	3.01%
T4-2	67.25%	18.40%	2.81%	0.12%	0.08%	0.00%	0.00%	0.00%	0.02%	0.01%	0.00%	11.30%
T4-3	35.26%	56.54%	1.97%	0.34%	0.05%	0.00%	0.00%	0.00%	0.01%	0.00%	0.01%	5.84%
T6-1	10.50%	89.30%	0.07%	0.01%	0.00%	0.00%	0.00%	0.00%	0.00%	0.00%	0.00%	0.12%
T6-2	11.03%	88.62%	0.10%	0.01%	0.00%	0.00%	0.00%	0.00%	0.00%	0.00%	0.01%	0.22%
T6-3	9.15%	90.39%	0.19%	0.00%	0.00%	0.00%	0.00%	0.00%	0.00%	0.00%	0.00%	0.26%
T8-1	14.84%	84.95%	0.11%	0.01%	0.00%	0.00%	0.00%	0.00%	0.00%	0.00%	0.00%	0.08%
T8-2	11.17%	88.65%	0.05%	0.01%	0.01%	0.00%	0.00%	0.00%	0.00%	0.00%	0.00%	0.12%
T8-3	12.64%	87.15%	0.08%	0.01%	0.00%	0.00%	0.00%	0.00%	0.00%	0.00%	0.00%	0.11%
T10-1	52.70%	46.70%	0.16%	0.01%	0.00%	0.00%	0.00%	0.00%	0.00%	0.00%	0.00%	0.42%
T10-2	14.21%	85.64%	0.05%	0.00%	0.00%	0.00%	0.00%	0.00%	0.00%	0.00%	0.00%	0.10%
T10-3	12.16%	87.68%	0.06%	0.01%	0.00%	0.00%	0.00%	0.00%	0.00%	0.00%	0.00%	0.09%
T12-1	18.40%	79.88%	0.18%	0.12%	0.00%	0.00%	0.00%	0.15%	0.00%	0.00%	0.02%	1.26%
T12-2	13.12%	86.68%	0.08%	0.00%	0.00%	0.00%	0.00%	0.00%	0.00%	0.00%	0.02%	0.10%
T12-3	20.02%	79.77%	0.11%	0.01%	0.01%	0.00%	0.00%	0.00%	0.00%	0.00%	0.00%	0.09%
T14-1	19.48%	80.26%	0.09%	0.02%	0.00%	0.00%	0.00%	0.00%	0.00%	0.00%	0.00%	0.15%

T14-2	17.37%	82.55%	0.02%	0.00%	0.00%	0.00%	0.00%	0.00%	0.00%	0.00%	0.00%	0.06%
T14-3	53.17%	46.29%	0.16%	0.01%	0.00%	0.00%	0.00%	0.00%	0.00%	0.00%	0.00%	0.37%
T16-1	20.38%	79.24%	0.07%	0.00%	0.00%	0.00%	0.00%	0.00%	0.00%	0.00%	0.01%	0.30%
T16-2	23.99%	75.60%	0.06%	0.03%	0.00%	0.02%	0.02%	0.01%	0.00%	0.00%	0.00%	0.27%
T16-3	25.73%	63.44%	2.17%	1.34%	0.00%	1.21%	1.00%	0.47%	0.00%	0.00%	0.01%	4.64%
T18-1	20.35%	79.20%	0.07%	0.01%	0.00%	0.00%	0.00%	0.00%	0.00%	0.00%	0.00%	0.37%
T18-2	30.71%	68.83%	0.03%	0.00%	0.00%	0.00%	0.00%	0.00%	0.00%	0.00%	0.02%	0.40%
T18-3	21.55%	77.89%	0.19%	0.01%	0.00%	0.00%	0.00%	0.00%	0.00%	0.00%	0.02%	0.34%
D0-1	39.02%	0.82%	2.01%	0.28%	0.09%	0.00%	0.00%	0.00%	0.02%	0.00%	0.01%	57.76%
D0-2	37.09%	0.98%	1.99%	0.38%	0.10%	0.00%	0.00%	0.00%	0.05%	0.01%	0.00%	59.41%
D0-3	20.84%	0.31%	1.01%	0.24%	0.05%	0.00%	0.00%	0.00%	0.00%	0.00%	0.00%	77.54%
D2-1	83.52%	0.39%	4.81%	0.40%	0.24%	0.00%	0.00%	0.00%	0.01%	0.01%	0.03%	10.57%
D2-2	78.54%	0.64%	3.92%	0.50%	0.26%	0.00%	0.00%	0.03%	0.08%	0.01%	0.04%	15.98%
D2-3	78.74%	0.43%	2.82%	0.31%	0.20%	0.00%	0.00%	0.02%	0.06%	0.03%	0.01%	17.39%
D4-1	95.20%	0.18%	1.40%	0.30%	0.10%	0.00%	0.00%	0.01%	0.00%	0.01%	0.01%	2.79%
D4-2	91.50%	0.19%	3.25%	0.29%	0.28%	0.00%	0.00%	0.00%	0.01%	0.02%	0.01%	4.45%
D4-3	93.20%	0.22%	3.00%	0.27%	0.07%	0.00%	0.00%	0.00%	0.02%	0.01%	0.00%	3.20%
D6-1	86.61%	0.27%	1.59%	5.26%	0.19%	0.00%	0.00%	0.04%	0.04%	0.00%	0.01%	5.99%

D6-2	94.19%	0.30%	1.74%	0.17%	0.07%	0.00%	0.00%	0.00%	0.01%	0.00%	0.01%	3.51%
D6-3	93.44%	0.79%	2.79%	0.24%	0.33%	0.00%	0.00%	0.00%	0.01%	0.01%	0.01%	2.38%
D8-1	58.51%	39.89%	0.45%	0.12%	0.08%	0.00%	0.00%	0.03%	0.02%	0.01%	0.05%	0.85%
D8-2	46.48%	51.71%	0.44%	0.05%	0.07%	0.00%	0.00%	0.00%	0.00%	0.00%	0.00%	1.25%
D8-3	62.58%	35.69%	0.67%	0.07%	0.11%	0.00%	0.00%	0.00%	0.00%	0.00%	0.00%	0.87%
D10-1	79.74%	19.60%	0.24%	0.03%	0.06%	0.00%	0.00%	0.00%	0.00%	0.00%	0.00%	0.34%
D10-2	62.98%	36.52%	0.10%	0.06%	0.02%	0.00%	0.00%	0.00%	0.00%	0.00%	0.00%	0.31%
D10-3	78.17%	21.16%	0.27%	0.06%	0.03%	0.00%	0.00%	0.00%	0.00%	0.00%	0.01%	0.30%
D12-1	97.97%	1.61%	0.06%	0.07%	0.00%	0.00%	0.00%	0.00%	0.00%	0.00%	0.02%	0.26%
D12-2	95.07%	4.52%	0.10%	0.05%	0.10%	0.00%	0.00%	0.00%	0.00%	0.00%	0.01%	0.14%
D12-3	96.39%	2.59%	0.10%	0.40%	0.01%	0.00%	0.00%	0.02%	0.00%	0.00%	0.03%	0.46%
D14-1	98.14%	1.26%	0.03%	0.47%	0.00%	0.00%	0.00%	0.02%	0.00%	0.00%	0.02%	0.06%
D14-2	99.08%	0.59%	0.03%	0.14%	0.00%	0.00%	0.00%	0.00%	0.00%	0.00%	0.01%	0.14%
D14-3	98.56%	0.98%	0.03%	0.27%	0.00%	0.00%	0.00%	0.01%	0.00%	0.00%	0.02%	0.14%
D16-1	98.95%	0.93%	0.01%	0.05%	0.00%	0.00%	0.00%	0.00%	0.00%	0.00%	0.00%	0.05%
D16-2	99.64%	0.24%	0.03%	0.01%	0.00%	0.00%	0.00%	0.00%	0.00%	0.00%	0.01%	0.06%
D16-3	99.48%	0.45%	0.01%	0.02%	0.00%	0.00%	0.00%	0.00%	0.00%	0.00%	0.01%	0.04%
D18-1	99.63%	0.31%	0.01%	0.01%	0.00%	0.00%	0.00%	0.00%	0.00%	0.00%	0.01%	0.04%

D18-2	97.35%	2.05%	0.13%	0.07%	0.00%	0.00%	0.00%	0.00%	0.00%	0.00%	0.03%	0.38%
D18-3	98.63%	1.26%	0.02%	0.02%	0.00%	0.00%	0.00%	0.00%	0.00%	0.00%	0.02%	0.04%

Supplementary Table 9. Changes of phyla level of fungi under traditional inoculation (T) and direct inoculation (D) (relative abundance>1%).

Fermentation Days	Ascomycota	Basidiomycota	Mucoromycota	Unassigned
T0-1	6.96%	0.04%	0.02%	92.99%
T0-2	8.36%	0.10%	0.04%	91.50%
T0-3	13.09%	0.08%	0.04%	86.80%
T2-1	19.76%	0.03%	0.11%	80.09%
T2-2	20.59%	0.02%	0.03%	79.35%
T2-3	24.07%	0.05%	0.06%	75.82%
T4-1	50.38%	0.17%	0.07%	49.38%
T4-2	37.50%	0.17%	0.08%	62.26%
T4-3	29.03%	0.13%	0.04%	70.80%
T6-1	29.13%	0.13%	0.05%	70.69%
T6-2	56.21%	0.83%	0.10%	42.86%
T6-3	42.62%	0.89%	0.07%	56.41%

T8-1	51.61%	0.24%	0.13%	48.01%
T8-2	45.28%	0.24%	0.12%	54.37%
T8-3	45.28%	0.24%	0.12%	54.37%
T10-1	44.63%	0.41%	0.08%	54.87%
T10-2	44.12%	0.22%	0.21%	55.46%
T10-3	26.38%	0.16%	0.06%	73.40%
T12-1	66.51%	1.32%	0.57%	31.59%
T12-2	55.01%	0.03%	0.25%	44.71%
T12-3	47.94%	0.50%	0.00%	51.56%
T14-1	67.25%	0.55%	0.00%	32.20%
T14-2	59.68%	0.35%	0.00%	39.98%
T14-3	57.69%	0.49%	0.58%	41.24%
T16-1	56.37%	2.14%	0.42%	41.07%
T16-2	38.34%	0.68%	0.07%	60.92%
T16-3	58.37%	0.34%	1.82%	39.48%
T18-1	69.03%	0.00%	0.00%	30.96%
T18-2	37.91%	0.15%	0.07%	61.87%
T18-3	53.62%	0.00%	0.00%	46.37%

D0-1	11.91%	0.13%	0.13%	87.83%
D0-2	11.23%	0.10%	0.09%	88.59%
D0-3	8.36%	0.11%	0.07%	91.46%
D2-1	28.58%	0.38%	0.04%	71.00%
D2-2	32.50%	0.39%	0.04%	67.06%
D2-3	39.31%	0.18%	0.11%	60.40%
D4-1	57.19%	0.65%	0.06%	42.10%
D4-2	65.83%	0.52%	0.08%	33.57%
D4-3	74.53%	3.15%	0.05%	22.27%
D6-1	73.56%	0.77%	0.11%	25.56%
D6-2	93.68%	0.77%	0.43%	5.12%
D6-3	66.90%	0.41%	0.06%	32.64%
D8-1	91.30%	0.87%	0.11%	7.72%
D8-2	92.80%	0.60%	0.06%	6.54%
D8-3	89.84%	0.38%	0.02%	9.76%
D10-1	84.92%	0.45%	0.05%	14.57%
D10-2	84.03%	0.53%	0.08%	15.36%
D10-3	86.27%	1.05%	0.00%	12.67%

D12-1	93.39%	1.74%	0.11%	4.76%
D12-2	92.74%	0.28%	0.00%	6.98%
D12-3	92.60%	2.04%	0.09%	5.26%
D14-1	62.54%	0.21%	0.00%	37.24%
D14-2	86.23%	0.61%	0.19%	12.97%
D14-3	81.70%	0.56%	0.00%	17.74%
D16-1	83.39%	1.45%	0.00%	15.16%
D16-2	79.71%	0.21%	1.07%	19.01%
D16-3	95.90%	0.00%	0.72%	3.38%
D18-1	92.65%	1.37%	0.00%	5.98%
D18-2	92.76%	0.00%	1.06%	6.18%
D18-3	90.53%	0.00%	0.00%	9.47%

Supplementary Table 9. Change of microbial species level under traditional inoculation (T) and direct inoculation (D) (relative abundance>10%).

Fermentation Days	Lactobacillus_acetotolerans(16S)	Acetobacter_pasteurianus(16S)	Agrobacterium_larrymoorei(16S)	Stenotrophomonas_maltophilia(16S)	Xanthomonas_sacchari(16S)	Alternaria_Unclassified(ITS)	Saccharomyces_Unclassified(ITS)
T0-1	1.07%	0.33%	3.95%	0.22%	2.14%	3.73%	2.72%

T0-2	4.75%	0.85%	8.77%	0.49%	5.32%	4.65%	3.00%
T0-3	4.06%	0.86%	5.96%	0.37%	4.42%	7.88%	3.98%
T2-1	5.35%	1.92%	15.97%	1.62%	5.57%	0.62%	19.00%
T2-2	5.24%	2.72%	16.34%	0.87%	7.60%	0.86%	19.56%
T2-3	6.09%	2.07%	16.18%	0.93%	6.00%	0.61%	23.25%
T4-1	65.25%	19.20%	3.44%	0.27%	0.80%	2.38%	47.23%
T4-2	17.86%	42.75%	10.95%	0.07%	0.39%	1.67%	35.31%
T4-3	55.93%	18.55%	6.23%	0.53%	1.96%	0.60%	28.05%
T6-1	89.01%	9.50%	0.29%	0.03%	0.15%	0.49%	28.45%
T6-2	88.20%	9.95%	0.28%	0.04%	0.13%	2.74%	51.38%
T6-3	90.07%	7.84%	0.48%	0.06%	0.12%	1.08%	40.86%
T8-1	84.74%	14.03%	0.20%	0.04%	0.11%	0.89%	50.31%
T8-2	88.45%	10.54%	0.17%	0.01%	0.10%	1.19%	43.10%
T8-3	86.92%	11.96%	0.14%	0.02%	0.11%	1.19%	43.10%
T10-1	46.55%	51.06%	0.63%	0.02%	0.10%	2.91%	40.20%
T10-2	85.54%	13.77%	0.13%	0.01%	0.06%	1.49%	40.78%
T10-3	87.57%	11.44%	0.22%	0.01%	0.08%	0.78%	25.05%
T12-1	79.57%	17.48%	0.09%	0.06%	0.05%	6.67%	50.28%

T12-2	86.60%	12.53%	0.19%	0.02%	0.09%	2.71%	51.39%
T12-3	79.71%	19.22%	0.15%	0.12%	0.08%	1.92%	44.46%
T14-1	80.06%	18.49%	0.44%	0.01%	0.05%	1.97%	62.32%
T14-2	82.45%	16.98%	0.06%	0.04%	0.04%	3.31%	54.14%
T14-3	46.20%	52.05%	0.37%	0.00%	0.01%	2.79%	50.89%
T16-1	79.11%	19.49%	0.13%	0.01%	0.04%	4.71%	49.67%
T16-2	75.52%	23.18%	0.17%	0.02%	0.04%	4.42%	32.18%
T16-3	62.85%	15.59%	0.25%	0.04%	0.03%	5.98%	47.72%
T18-1	78.88%	18.01%	0.19%	0.02%	0.01%	3.20%	65.33%
T18-2	68.73%	28.35%	0.13%	0.02%	0.06%	1.93%	33.65%
T18-3	77.30%	17.74%	0.04%	0.01%	0.02%	1.46%	43.61%
D0-1	0.34%	1.47%	10.86%	0.72%	5.55%	5.90%	4.45%
D0-2	0.88%	1.93%	13.28%	0.05%	0.46%	5.04%	4.92%
D0-3	0.02%	0.97%	4.76%	0.50%	2.99%	4.38%	2.90%
D2-1	0.06%	11.41%	18.20%	1.84%	11.91%	1.88%	25.68%
D2-2	0.20%	5.91%	18.83%	2.23%	10.71%	3.64%	27.38%
D2-3	0.03%	7.18%	19.54%	2.76%	11.45%	1.24%	37.60%

D4-1	0.02%	37.92%	20.30%	1.52%	7.86%	1.94%	52.62%
D4-2	0.03%	24.31%	23.08%	1.67%	9.37%	1.38%	63.43%
D4-3	0.02%	34.16%	20.63%	1.39%	7.73%	2.43%	66.05%
D6-1	0.00%	34.01%	23.53%	0.19%	0.79%	2.69%	68.81%
D6-2	0.03%	23.98%	19.28%	26.82%	3.26%	1.60%	89.90%
D6-3	0.03%	30.73%	28.56%	2.62%	4.62%	2.01%	62.68%
D8-1	0.04%	40.90%	6.54%	0.67%	1.36%	2.93%	84.36%
D8-2	0.03%	29.06%	7.18%	0.51%	1.34%	1.73%	89.03%
D8-3	0.04%	40.79%	7.88%	0.94%	2.92%	1.14%	87.13%
D10-1	0.17%	72.10%	2.65%	0.31%	0.77%	1.57%	81.88%
D10-2	0.27%	56.50%	2.49%	0.48%	0.51%	1.59%	79.49%
D10-3	0.05%	68.12%	5.17%	0.26%	0.11%	1.64%	81.98%
D12-1	0.23%	93.24%	1.32%	0.15%	0.62%	1.81%	81.76%
D12-2	1.41%	90.21%	1.13%	0.18%	0.71%	2.17%	87.30%
D12-3	0.66%	91.90%	0.76%	0.14%	0.57%	5.59%	75.33%
D14-1	0.45%	96.30%	0.27%	0.03%	0.15%	3.89%	48.63%
D14-2	0.14%	97.59%	0.39%	0.05%	0.21%	2.55%	76.97%
D14-3	0.26%	97.17%	0.29%	0.06%	0.15%	1.52%	71.95%

D16-1	0.55%	98.30%	0.14%	0.03%	0.06%	2.39%	75.59%
D16-2	0.08%	99.28%	0.12%	0.00%	0.01%	2.21%	66.10%
D16-3	0.28%	99.09%	0.05%	0.01%	0.02%	1.53%	87.65%
D18-1	0.18%	99.40%	0.04%	0.00%	0.01%	2.02%	79.53%
D18-2	1.83%	96.71%	0.04%	0.01%	0.02%	11.10%	77.60%
D18-3	1.04%	98.12%	0.09%	0.01%	0.03%	0.73%	84.62%

Supplementary Table 10. Correlation table between core microorganisms (relative abundance greater than 10%) and volatile flavor substances in acetic acid fermentation under traditional inoculation and direct injection inoculation.

	positive				negative			
**Core microorganisms**	**Traditional inoculation**		**Direct inoculation**		**Traditional inoculation**		**Direct inoculation**	
	**Volatile flavor substances**	**pvalue**	**Volatile flavor substances**	**pvalue**	**Volatile flavor substances**	**pvalue**	**Volatile flavor substances**	**pvalue**
Acetobacter_pasteurianus(16S)	1,3-Dioxolane, 2,4,5-trimethyl-	0.02	1,3-Dioxan-5-ol	0.01	2-Methoxy-5-methylphenol	0.80	(2-Aziridinylethyl)amine	0.00
	12-Crown-4	0.04	1,3-Dioxolane, 2,4,5-trimethyl-	0.00	Butyrolactone	0.00	(E)-9-Octadecenoic acid ethyl ester	0.25
	17-Octadecynoic acid	0.14	1-Butanol, 3-methyl-, acetate	0.00	Cyclooctasiloxane, hexadecamethyl-	0.18	(Z)-4-Decen-1-ol	0.16

1-Butanol, 3-methyl-	0.57	2,3-Butanediol, [S-(R*,R*)]-	0.00	Cyclotetrasiloxane, octamethyl-	0.77	10-Undecen-1-ol	0.21
1-Butanol, 3-methyl-, acetate	0.02	2,3-Butanedione	0.19	Diethyl azelate	0.69	13,16-Octadecadiynoic acid, methyl ester	0.08
1-Hexanol	0.34	2-Methoxy-4-vinylphenol	0.00	Dimethyl ether	0.00	1-Butanol, 3-methyl-	0.00
1-Propanol, 2-methyl-	0.50	Acetic acid	0.00	Dodecanoic acid, ethyl ester	0.57	1-Heptanol	0.20
2(3H)-Furanone, dihydro-5-pentyl-	0.05	Acetic acid, butyl ester	0.05	Ethane, 1,1-diethoxy-	0.01	1-Hexanol	0.01
2,2'-Bifuran, octahydro-	0.12	Acetic acid, hexyl ester	0.02	Ethene, ethoxy-	0.02	1-Nonanol	0.01
2,3-Butanediol, [S-(R*,R*)]-	0.57	Acetoin	0.07	Ethyl Acetate	0.46	1-Octanol	0.18
2-Propenoic acid, 2-methyl-, hexyl ester	0.16	Benzeneacetaldehyde, .alpha.-ethylidene-	0.00	Formic acid, 1-methylethyl ester	0.53	1-Oxa-3,4-diazacyclopentadiene	0.57
3-Methyl-hepta-1,6-dien-3-ol	0.17	Benzeneethanol, b-ethyl-	0.00	Formic acid, hexyl ester	0.16	1-Propanol	0.86
Acetic acid	0.00	Butanedioic acid, diethyl ester	0.05	Hexanoic acid, ethyl ester	0.91	1-Propanol, 2-methyl-	0.00
Acetic acid ethenyl ester	0.17	Butanoic acid	0.23	Linoleic acid ethyl ester	0.34	2,3-Butanediol	0.00
Acetic acid, 2-phenylethyl	0.18	Butanoic acid, 3-	0.00	Naphthalene	0.97	2,4-Di-tert-	0.05

ester		methyl-				butylphenol	
Acetic acid, butyl ester	0.13	Cycloheptasiloxane, tetradecamethyl-	0.15	Oxirane, 2-methyl-2-phenyl-	0.00	2-Butanol, 3-methyl-, (S)-	0.08
Acetic acid, diethyl-	0.19	Cyclohexasiloxane, dodecamethyl-	0.02	Pentane, 2,3,4-trimethyl-	0.22	3-Methyl-hepta-1,6-dien-3-ol	0.05
Acetic acid, heptyl ester	0.48	Cyclotetrasiloxane, octamethyl-	0.80	Phenylethyl Alcohol	0.76	4-Methylcyclohexaneacetic acid	0.23
Acetic acid, hexyl ester	0.29	Cyclotrisiloxane, hexamethyl-	0.03	Propanoic acid, anhydride	0.12	6-Nonynoic acid	0.28
Acetic acid, methyl ester	0.03	Decanoic acid, ethyl ester	0.80	Trichloroacetic acid, dodec-9-ynyl ester	0.02	7-Octenoic acid, ethyl ester	0.23
Acetic acid, non-3-enyl ester, cis-	0.23	Ethanol	0.02	Undecylenic acid	0.02	8-Methylnonanoic acid, ethyl ester	0.02
Acetic acid, nonyl ester	0.15	Ethyl 4-acetoxybutanoate	0.00			9(E),11(E)-Conjugated linoleic acid, ethyl ester	0.02
Acetic acid, octyl ester	0.05	Furfural	0.00			9-Octadecenoic acid, ethyl ester	0.00
Acetic acid, pentyl ester	0.96	gamma-Decalactone	0.00			Acetic acid, 2-phenylethyl ester	0.00
Acetoin	0.01	Heptanoic acid	0.00			Acetoxyacetic acid, 4-pentadecyl ester	0.19

Alanine	0.15	Hexanoic acid	0.00	Alanine	0.01
Benzaldehyde	0.17	Hexyl methacrylate	0.87	alpha-Terpineol	0.04
Benzaldehyde, 4-pentyl-	0.57	Isobutyl acetate	0.00	Amylene hydrate	0.00
Benzene, 1-methyl-2-propyl-	0.23	Linoleic acid ethyl ester	0.78	Azulene	0.02
Benzeneacetaldehyde	0.32	Methane, isocyanato-	0.00	Benzaldehyde	0.41
Benzeneacetaldehyde, .alpha.-ethyl-	0.57	Methyl vinyl ketone	0.02	Benzeneacetaldehyde	0.00
Benzeneacetaldehyde, .alpha.-ethylidene-	0.15	n-Propyl acetate	0.04	Benzeneacetic acid, ethyl ester	0.92
Benzeneacetic acid, ethyl ester	0.01	Octanoic acid	0.00	Benzoic acid, ethyl ester	0.00
Benzenepropanoic acid, .alpha.-(1-hydroxyethyl)-	0.17	Oxirane, 3-hydroxypropyl-	0.08	Butanal	0.19
Benzoic acid, ethyl ester	0.07	Pentanoic acid	0.17	Butanal, 2-methyl-	0.17
Butanal, 3-methyl-	0.04	Propanoic acid, 2-methyl-	0.09	Butanal, 3-methyl-	0.00
Butanedioic acid, diethyl ester	0.53			Butyrolactone	0.00
Butanoic acid	0.43			Cyclobutanol	0.69

Butanoic acid, 2-methyl-, ethyl ester	0.10	Cyclooctasiloxane, hexadecamethyl-	0.57
Butanoic acid, 3-methyl-	0.04	Cyclopentasiloxane, decamethyl-	0.64
Butanoic acid, ethyl ester	0.15	Diethyl azelate	0.00
Creosol	0.03	Dodecanoic acid, ethyl ester	0.03
Cyclobutanol	0.18	Ethane, 1,1-diethoxy-	0.00
Cycloheptasiloxane, tetradecamethyl-	0.87	Ethyl Acetate	0.00
Cyclohexasiloxane, dodecamethyl-	0.57	gamma-Nonanolactone	0.95
Cyclopentasiloxane, decamethyl-	0.69	Heptanoic acid, ethyl ester	0.05
Cyclotrisiloxane, hexamethyl-	0.61	Hexadecanoic acid, ethyl ester	0.00
Decanoic acid, ethyl ester	0.22	Hexanoic acid, 2-methylpropyl ester	0.03
Dextroamphetamine	0.14	Hexanoic acid, ethyl ester	0.00
Ethanol	0.64	Isopentyl 4-methylpentanoate	0.15

Ethyl 4-acetoxybutanoate	0.09	Isopropyl Alcohol	0.00
Furfural	0.14	Lactic acid	0.03
Guanidine, N,N-dimethyl-	0.61	Naphthalene	0.01
Heptanoic acid, ethyl ester	0.09	Nonanal	0.33
Hexadecanoic acid, ethyl ester	0.23	Nonanoic acid, ethyl ester	0.00
Hexanoic acid	0.30	Octanoic acid, ethyl ester	0.00
Isobutyl acetate	0.01	Pentadecanoic acid, ethyl ester	0.02
Isobutyl isopentyl carbonate	0.57	Pentanoic acid, ethyl ester	0.00
m-Dioxan-4-ol, 2,6-dimethyl-	0.23	Propanal, 2-methyl-	0.00
Methane, isocyanato-	0.19	Sulfurous acid, dipentyl ester	0.57
Nonanal	0.10	Tetradecanoic acid, ethyl ester	0.00
Nonanoic acid, ethyl ester	0.57		
n-Propyl acetate	0.34		

	Octanoic acid	0.17						
	Oxirane, 3-hydroxypropyl-	0.57						
	Pentanedioic acid	0.69						
	Pentanoic acid	0.95						
	Pentanoic acid, 2-hydroxy-4-methyl-, ethyl ester	0.62						
	Pentanoic acid, 3-methyl-	0.48						
	Pentanoic acid, ethyl ester	0.40						
	Phenol, 4-ethyl-2-methoxy-	0.17						
	Phenol, 5-ethenyl-2-methoxy-	0.17						
	Propanoic acid, 2-hydroxy-, ethyl ester	0.72						
	Propanoic acid, 2-methyl-	0.40						
	1-Butanol, 3-methyl-	0.92	(2-Aziridinylethyl)amine	0.01	1,3-Dioxolane, 2,4,5-trimethyl-	0.00	1,3-Dioxan-5-ol	0.00
Agrobacterium_larry moorei(16S)	1-Hexanol	0.53	(E)-9-Octadecenoic acid ethyl ester	0.06	12-Crown-4	0.07	1,3-Dioxolane, 2,4,5-trimethyl-	0.00
	2,2'-Bifuran, octahydro-	0.08	(Z)-4-Decen-1-ol	0.01	17-Octadecynoic acid	0.64	1-Butanol, 3-methyl-, acetate	0.00

Benzeneacetaldehyde	0.41	10-Undecen-1-ol	0.01	1-Butanol, 3-methyl-, acetate	0.00	2,3-Butanediol, [S-(R*,R*)]-	0.00
Butanedioic acid, diethyl ester	0.51	13,16-Octadecadiynoic acid, methyl ester	0.25	1-Propanol, 2-methyl-	0.96	2,3-Butanedione	0.28
Butanoic acid, 2-methyl-, ethyl ester	0.14	1-Butanol, 3-methyl-	0.00	2(3H)-Furanone, dihydro-5-pentyl-	0.00	2-Methoxy-4-vinylphenol	0.00
Butyrolactone	0.06	1-Heptanol	0.07	2,3-Butanediol, [S-(R*,R*)]-	0.01	Acetic acid	0.00
Cyclooctasiloxane, hexadecamethyl-	0.86	1-Hexanol	0.00	2-Methoxy-5-methylphenol	0.00	Acetic acid, butyl ester	0.06
Cyclotetrasiloxane, octamethyl-	0.87	1-Nonanol	0.00	2-Propenoic acid, 2-methyl-, hexyl ester	0.24	Acetic acid, hexyl ester	0.04
Decanoic acid, ethyl ester	0.09	1-Octanol	0.01	3-Methyl-hepta-1,6-dien-3-ol	0.06	Acetoin	0.00
Diethyl azelate	0.00	1-Oxa-3,4-diazacyclopentadiene	0.61	Acetic acid	0.00	Benzeneacetaldehyde, .alpha.-ethylidene-	0.03
Dimethyl ether	0.00	1-Propanol	0.31	Acetic acid ethenyl ester	0.05	Benzeneethanol, b-ethyl-	0.00
Dodecanoic acid, ethyl ester	0.46	1-Propanol, 2-methyl-	0.00	Acetic acid, 2-phenylethyl ester	0.97	Butanedioic acid, diethyl ester	0.28
Ethane, 1,1-diethoxy-	0.00	2,3-Butanediol	0.13	Acetic acid, butyl ester	0.44	Butanoic acid	0.19

Ethene, ethoxy-	0.14	2,4-Di-tert-butylphenol	0.00	Acetic acid, diethyl-	0.28	Butanoic acid, 3-methyl-	0.00
Ethyl Acetate	0.21	2-Butanol, 3-methyl-, (S)-	0.43	Acetic acid, heptyl ester	0.08	Cycloheptasiloxane, tetradecamethyl-	0.55
Formic acid, hexyl ester	0.55	3-Methyl-hepta-1,6-dien-3-ol	0.01	Acetic acid, hexyl ester	0.07	Cyclohexasiloxane, dodecamethyl-	0.07
Heptanoic acid, ethyl ester	0.13	4-Methylcyclohexaneacetic acid	0.28	Acetic acid, methyl ester	0.05	Cyclotetrasiloxane, octamethyl-	0.75
Hexanoic acid, ethyl ester	0.04	6-Nonynoic acid	0.46	Acetic acid, non-3-enyl ester, cis-	0.04	Cyclotrisiloxane, hexamethyl-	0.23
Nonanoic acid, ethyl ester	0.02	7-Octenoic acid, ethyl ester	0.28	Acetic acid, nonyl ester	0.30	Ethanol	0.07
Oxirane, 2-methyl-2-phenyl-	0.06	8-Methylnonanoic acid, ethyl ester	0.01	Acetic acid, octyl ester	0.05	Ethyl 4-acetoxybutanoate	0.00
Pentane, 2,3,4-trimethyl-	0.68	9(E),11(E)-Conjugated linoleic acid, ethyl ester	0.67	Acetic acid, pentyl ester	0.96	Furfural	0.00
Pentanoic acid, ethyl ester	0.11	9-Octadecenoic acid, ethyl ester	0.00	Acetoin	0.00	gamma-Decalactone	0.00
Propanoic acid, anhydride	0.31	Acetic acid, 2-phenylethyl ester	0.00	Alanine	0.56	Heptanoic acid	0.00
Trichloroacetic acid, dodec-9-ynyl ester	0.14	Acetoxyacetic acid, 4-pentadecyl ester	0.40	Benzaldehyde	0.00	Hexanoic acid	0.00

Undecylenic acid	0.14	Alanine	0.38	Benzaldehyde, 4-pentyl-	0.42	Hexyl methacrylate	0.95
		alpha-Terpineol	0.00	Benzene, 1-methyl-2-propyl-	0.04	Isobutyl acetate	0.00
		Amylene hydrate	0.00	Benzeneacetaldehyde, .alpha.-ethyl-	0.42	Linoleic acid ethyl ester	0.87
		Azulene	0.15	Benzeneacetaldehyde, .alpha.-ethylidene-	0.56	Methane, isocyanato-	0.00
		Benzaldehyde	0.09	Benzeneacetic acid, ethyl ester	0.02	Methyl vinyl ketone	0.00
		Benzeneacetaldehyde	0.00	Benzenepropanoic acid, .alpha.-(1-hydroxyethyl)-	0.05	n-Propyl acetate	0.06
		Benzeneacetic acid, ethyl ester	0.19	Benzoic acid, ethyl ester	0.02	Octanoic acid	0.01
		Benzoic acid, ethyl ester	0.00	Butanal, 3-methyl-	0.06	Oxirane, 3-hydroxypropyl-	0.15
		Butanal	0.40	Butanoic acid	0.36	Pentanoic acid	0.24
		Butanal, 2-methyl-	0.40	Butanoic acid, 3-methyl-	0.01	Propanoic acid, 2-methyl-	0.11
		Butanal, 3-methyl-	0.00	Butanoic acid, ethyl ester	0.56		

Butyrolactone	0.01	Creosol	0.05
Cyclobutanol	0.19	Cyclobutanol	0.06
Cyclooctasiloxane, hexadecamethyl-	0.61	Cycloheptasiloxane, tetradecamethyl-	0.60
Cyclopentasiloxane, decamethyl-	0.26	Cyclohexasiloxane, dodecamethyl-	0.50
Decanoic acid, ethyl ester	0.72	Cyclopentasiloxane, decamethyl-	0.75
Diethyl azelate	0.00	Cyclotrisiloxane, hexamethyl-	0.41
Dodecanoic acid, ethyl ester	0.04	Dextroamphetamine	0.61
Ethane, 1,1-diethoxy-	0.50	Ethanol	0.36
Ethyl Acetate	0.00	Ethyl 4-acetoxybutanoate	0.00
gamma-Nonanolactone	0.19	Formic acid, 1-methylethyl ester	0.27
Heptanoic acid, ethyl ester	0.00	Furfural	0.50
Hexadecanoic acid, ethyl ester	0.00	Guanidine, N,N-dimethyl-	0.69

Hexanoic acid, 2-methylpropyl ester	0.11	Hexadecanoic acid, ethyl ester	0.65
Hexanoic acid, ethyl ester	0.00	Hexanoic acid	0.05
Isopentyl 4-methylpentanoate	0.02	Isobutyl acetate	0.00
Isopropyl Alcohol	0.08	Isobutyl isopentyl carbonate	0.42
Lactic acid	0.09	Linoleic acid ethyl ester	0.79
Naphthalene	0.04	m-Dioxan-4-ol, 2,6-dimethyl-	0.04
Nonanal	0.25	Methane, isocyanato-	0.06
Nonanoic acid, ethyl ester	0.00	Naphthalene	0.36
Octanoic acid, ethyl ester	0.00	Nonanal	0.16
Pentadecanoic acid, ethyl ester	0.01	n-Propyl acetate	0.01
Pentanoic acid, ethyl ester	0.00	Octanoic acid	0.03

			Propanal, 2-methyl-	0.00	Oxirane, 3-hydroxypropyl-	0.01		
			Sulfurous acid, dipentyl ester	0.61	Pentanedioic acid	0.09		
			Tetradecanoic acid, ethyl ester	0.00	Pentanoic acid	0.12		
					Pentanoic acid, 2-hydroxy-4-methyl-, ethyl ester	0.02		
					Pentanoic acid, 3-methyl-	0.08		
					Phenol, 4-ethyl-2-methoxy-	0.05		
					Phenol, 5-ethenyl-2-methoxy-	0.03		
					Phenylethyl Alcohol	0.71		
					Propanoic acid, 2-hydroxy-, ethyl ester	0.02		
					Propanoic acid, 2-methyl-	0.01		
Alternaria_Unclassified(ITS)	1,3-Dioxolane, 2,4,5-trimethyl-	0.06	(2-Aziridinylethyl)amine	0.08	1-Butanol, 3-methyl-, acetate	0.98	(E)-9-Octadecenoic acid ethyl ester	0.56

12-Crown-4	0.75	1,3-Dioxolane, 2,4,5-trimethyl-	0.54	1-Hexanol	0.16	(Z)-4-Decen-1-ol	0.65
17-Octadecynoic acid	0.41	13,16-Octadecadiynoic acid, methyl ester	0.61	2,2'-Bifuran, octahydro-	0.18	1,3-Dioxan-5-ol	0.63
1-Butanol, 3-methyl-	0.42	1-Butanol, 3-methyl-	0.36	Acetic acid, 2-phenylethyl ester	0.13	10-Undecen-1-ol	0.97
1-Propanol, 2-methyl-	0.58	1-Butanol, 3-methyl-, acetate	0.74	Acetic acid, diethyl-	0.96	1-Heptanol	0.35
2(3H)-Furanone, dihydro-5-pentyl-	0.23	1-Propanol, 2-methyl-	0.19	Acetic acid, non-3-enyl ester, cis-	0.68	1-Hexanol	0.32
2,3-Butanediol, [S-(R*,R*)]-	0.29	2,3-Butanediol	0.34	Acetic acid, nonyl ester	0.98	1-Nonanol	0.24
2-Methoxy-5-methylphenol	0.62	2,3-Butanedione	0.69	Benzene, 1-methyl-2-propyl-	0.68	1-Octanol	0.47
2-Propenoic acid, 2-methyl-, hexyl ester	0.02	2-Methoxy-4-vinylphenol	0.84	Butanedioic acid, diethyl ester	0.13	1-Oxa-3,4-diazacyclopentadiene	0.48
3-Methyl-hepta-1,6-dien-3-ol	0.91	9(E),11(E)-Conjugated linoleic acid, ethyl ester	0.07	Butanoic acid, 2-methyl-, ethyl ester	0.94	1-Propanol	0.90
Acetic acid	0.05	9-Octadecenoic acid, ethyl ester	0.25	Cycloheptasiloxane, tetradecamethyl-	0.65	2,3-Butanediol, [S-(R*,R*)]-	0.85
Acetic acid ethenyl ester	0.94	Acetic acid, butyl ester	0.99	Cyclooctasiloxane, hexadecamethyl-	0.00	2,4-Di-tert-butylphenol	0.56

Acetic acid, butyl ester	0.24	Acetoin	0.48	Cyclotrisiloxane, hexamethyl-	0.93	2-Butanol, 3-methyl-, (S)-	0.82
Acetic acid, heptyl ester	0.84	Acetoxyacetic acid, 4-pentadecyl ester	0.33	Decanoic acid, ethyl ester	0.22	3-Methyl-hepta-1,6-dien-3-ol	0.32
Acetic acid, hexyl ester	0.75	Alanine	0.26	Diethyl azelate	0.01	4-Methylcyclohexaneacetic acid	0.15
Acetic acid, methyl ester	0.52	Amylene hydrate	0.36	Dimethyl ether	0.47	6-Nonynoic acid	0.78
Acetic acid, octyl ester	0.73	Azulene	0.17	Dodecanoic acid, ethyl ester	0.11	7-Octenoic acid, ethyl ester	0.15
Acetic acid, pentyl ester	0.15	Benzeneacetaldehyde	0.44	Ethane, 1,1-diethoxy-	0.98	8-Methylnonanoic acid, ethyl ester	0.22
Acetoin	0.01	Benzeneacetaldehyde, .alpha.-ethylidene-	0.81	Ethanol	0.37	Acetic acid	0.47
Alanine	0.38	Benzeneethanol, b-ethyl-	0.32	Formic acid, 1-methylethyl ester	0.16	Acetic acid, 2-phenylethyl ester	0.43
Benzaldehyde	0.71	Benzoic acid, ethyl ester	0.37	Heptanoic acid, ethyl ester	0.93	Acetic acid, hexyl ester	0.83
Benzaldehyde, 4-pentyl-	0.52	Butanal	0.33	Linoleic acid ethyl ester	0.13	alpha-Terpineol	0.59
Benzeneacetaldehyde	0.52	Butanal, 2-methyl-	0.48	m-Dioxan-4-ol, 2,6-dimethyl-	0.68	Benzaldehyde	0.50

Benzeneacetaldehyde, .alpha.-ethyl-	0.52	Butanal, 3-methyl-	0.87	Nonanoic acid, ethyl ester	0.15	Benzeneacetic acid, ethyl ester	0.74
Benzeneacetaldehyde, .alpha.-ethylidene-	0.38	Butyrolactone	0.28	Pentane, 2,3,4-trimethyl-	0.72	Butanedioic acid, diethyl ester	0.88
Benzeneacetic acid, ethyl ester	0.83	Cyclobutanol	0.61	Pentanedioic acid	0.61	Butanoic acid	0.09
Benzenepropanoic acid, .alpha.-(1-hydroxyethyl)-	0.94	Cycloheptasiloxane, tetradecamethyl-	0.90	Pentanoic acid	0.39	Butanoic acid, 3-methyl-	0.98
Benzoic acid, ethyl ester	0.46	Decanoic acid, ethyl ester	0.65	Pentanoic acid, ethyl ester	0.03	Cyclohexasiloxane, dodecamethyl-	0.38
Butanal, 3-methyl-	0.72	Ethane, 1,1-diethoxy-	0.01	Propanoic acid, anhydride	0.03	Cyclooctasiloxane, hexadecamethyl-	0.48
Butanoic acid	0.32	Ethyl 4-acetoxybutanoate	0.77			Cyclopentasiloxane, decamethyl-	0.33
Butanoic acid, 3-methyl-	0.02	Ethyl Acetate	0.66			Cyclotetrasiloxane, octamethyl-	0.70
Butanoic acid, ethyl ester	0.38	Furfural	0.79			Cyclotrisiloxane, hexamethyl-	0.93
Butyrolactone	0.02	gamma-Decalactone	0.75			Diethyl azelate	0.38
Creosol	0.52	gamma-Nonanolactone	0.82			Dodecanoic acid, ethyl ester	0.66

Cyclobutanol	0.92	Hexadecanoic acid, ethyl ester	0.10	Ethanol	0.70
Cyclohexasiloxane, dodecamethyl-	0.92	Isobutyl acetate	0.94	Heptanoic acid	0.42
Cyclopentasiloxane, decamethyl-	0.11	Isopropyl Alcohol	0.17	Heptanoic acid, ethyl ester	0.41
Cyclotetrasiloxane, octamethyl-	0.82	Naphthalene	0.95	Hexanoic acid	0.71
Dextroamphetamine	0.40	Nonanal	0.30	Hexanoic acid, 2-methylpropyl ester	0.73
Ethene, ethoxy-	0.11	Octanoic acid	0.70	Hexanoic acid, ethyl ester	0.72
Ethyl 4-acetoxybutanoate	0.22	Octanoic acid, ethyl ester	0.56	Hexyl methacrylate	0.39
Ethyl Acetate	0.00	Pentanoic acid	0.63	Isopentyl 4-methylpentanoate	0.07
Formic acid, hexyl ester	0.09	Propanal, 2-methyl-	0.12	Lactic acid	0.83
Furfural	0.64	Propanoic acid, 2-methyl-	0.13	Linoleic acid ethyl ester	0.33
Guanidine, N,N-dimethyl-	0.46	Tetradecanoic acid, ethyl ester	0.32	Methane, isocyanato-	0.28
Hexadecanoic acid, ethyl	0.41			Methyl vinyl ketone	0.98

ester			
Hexanoic acid	0.00	Nonanoic acid, ethyl ester	0.28
Hexanoic acid, ethyl ester	0.79	n-Propyl acetate	0.95
Isobutyl acetate	0.28	Oxirane, 3-hydroxypropyl-	0.43
Isobutyl isopentyl carbonate	0.52	Pentadecanoic acid, ethyl ester	0.33
Methane, isocyanato-	0.05	Pentanoic acid, ethyl ester	0.23
Naphthalene	0.17	Sulfurous acid, dipentyl ester	0.48
Nonanal	0.08		
n-Propyl acetate	0.20		
Octanoic acid	0.01		
Oxirane, 2-methyl-2-phenyl-	0.02		
Oxirane, 3-hydroxypropyl-	0.25		
Pentanoic acid, 2-hydroxy-4-methyl-, ethyl ester	0.54		
Pentanoic acid, 3-methyl-	0.84		

	Phenol, 4-ethyl-2-methoxy-	0.94						
	Phenol, 5-ethenyl-2-methoxy-	0.89						
	Phenylethyl Alcohol	0.03						
	Propanoic acid, 2-hydroxy-, ethyl ester	0.77						
	Propanoic acid, 2-methyl-	0.09						
	Trichloroacetic acid, dodec-9-ynyl ester	0.11						
	Undecylenic acid	0.12						
Lactobacillus_acetotolerans(16S)	1,3-Dioxolane, 2,4,5-trimethyl-	0.06	1,3-Dioxan-5-ol	0.01	12-Crown-4	0.94	(2-Aziridinylethyl)amine	0.34
	17-Octadecynoic acid	0.85	1,3-Dioxolane, 2,4,5-trimethyl-	0.00	2,2'-Bifuran, octahydro-	0.26	(E)-9-Octadecenoic acid ethyl ester	0.22
	1-Butanol, 3-methyl-	0.89	13,16-Octadecadiynoic acid, methyl ester	0.99	2-Propenoic acid, 2-methyl-, hexyl ester	0.75	(Z)-4-Decen-1-ol	0.01
	1-Butanol, 3-methyl-, acetate	0.00	1-Butanol, 3-methyl-, acetate	0.06	3-Methyl-hepta-1,6-dien-3-ol	0.86	10-Undecen-1-ol	0.04
	1-Hexanol	0.21	2,3-Butanediol, [S-(R*,R*)]-	0.04	Acetic acid ethenyl ester	0.86	1-Butanol, 3-methyl-	0.00
	1-Propanol, 2-methyl-	0.61	2,3-Butanedione	0.28	Acetic acid, diethyl-	0.78	1-Heptanol	0.07

2(3H)-Furanone, dihydro-5-pentyl-	0.01	2-Methoxy-4-vinylphenol	0.00	Acetic acid, methyl ester	0.99	1-Hexanol	0.00
2,3-Butanediol, [S-(R*,R*)]-	0.00	Acetic acid	0.00	Acetic acid, non-3-enyl ester, cis-	0.81	1-Nonanol	0.00
2-Methoxy-5-methylphenol	0.00	Acetic acid, butyl ester	0.03	Acetic acid, nonyl ester	0.53	1-Octanol	0.01
Acetic acid	0.07	Acetic acid, hexyl ester	0.12	Acetic acid, octyl ester	0.98	1-Oxa-3,4-diazacyclopentadiene	0.23
Acetic acid, 2-phenylethyl ester	0.69	Acetoin	0.04	Acetic acid, pentyl ester	0.61	1-Propanol	0.23
Acetic acid, butyl ester	0.82	Acetoxyacetic acid, 4-pentadecyl ester	0.78	Benzene, 1-methyl-2-propyl-	0.81	1-Propanol, 2-methyl-	0.01
Acetic acid, heptyl ester	0.54	Benzeneacetaldehyde, .alpha.-ethylidene-	0.17	Benzeneacetaldehyde	0.12	2,3-Butanediol	0.47
Acetic acid, hexyl ester	0.01	Benzeneethanol, b-ethyl-	0.00	Benzenepropanoic acid, .alpha.-(1-hydroxyethyl)-	0.86	2,4-Di-tert-butylphenol	0.02
Acetoin	0.79	Butanal	0.78	Butanal, 3-methyl-	0.98	2-Butanol, 3-methyl-, (S)-	0.08
Alanine	0.84	Butanedioic acid, diethyl ester	0.75	Butanoic acid, 2-methyl-, ethyl ester	0.33	3-Methyl-hepta-1,6-dien-3-ol	0.08
Benzaldehyde	0.00	Butanoic acid	0.15	Butyrolactone	0.00	4-Methylcyclohexaneac	0.23

						etic acid	
Benzaldehyde, 4-pentyl-	0.42	Butanoic acid, 3-methyl-	0.00	Creosol	0.99	6-Nonynoic acid	0.78
Benzeneacetaldehyde, .alpha.-ethyl-	0.42	Cyclohexasiloxane, dodecamethyl-	0.29	Cyclobutanol	0.87	7-Octenoic acid, ethyl ester	0.23
Benzeneacetaldehyde, .alpha.-ethylidene-	0.84	Cyclotetrasiloxane, octamethyl-	0.62	Cyclopentasiloxane, decamethyl-	0.74	8-Methylnonanoic acid, ethyl ester	0.07
Benzeneacetic acid, ethyl ester	0.12	Cyclotrisiloxane, hexamethyl-	0.71	Decanoic acid, ethyl ester	0.48	9(E),11(E)-Conjugated linoleic acid, ethyl ester	0.92
Benzoic acid, ethyl ester	0.61	Ethane, 1,1-diethoxy-	0.63	Dimethyl ether	0.00	9-Octadecenoic acid, ethyl ester	0.12
Butanedioic acid, diethyl ester	0.08	Ethanol	0.11	Ethane, 1,1-diethoxy-	0.00	Acetic acid, 2-phenylethyl ester	0.01
Butanoic acid	0.49	Ethyl 4-acetoxybutanoate	0.00	Ethene, ethoxy-	0.02	Alanine	0.32
Butanoic acid, 3-methyl-	0.48	Furfural	0.01	Ethyl Acetate	0.00	alpha-Terpineol	0.02
Butanoic acid, ethyl ester	0.84	gamma-Decalactone	0.01	Formic acid, hexyl ester	0.24	Amylene hydrate	0.08
Cycloheptasiloxane, tetradecamethyl-	0.03	Heptanoic acid	0.23	Heptanoic acid, ethyl ester	0.32	Azulene	0.25
Cyclohexasiloxane,	0.07	Hexanoic acid	0.03	Hexadecanoic acid,	0.79	Benzaldehyde	0.11

dodecamethyl-				ethyl ester			
Cyclooctasiloxane, hexadecamethyl-	0.35	Isobutyl acetate	0.00	Hexanoic acid, ethyl ester	0.19	Benzeneacetaldehyde	0.01
Cyclotetrasiloxane, octamethyl-	0.25	Linoleic acid ethyl ester	0.96	m-Dioxan-4-ol, 2,6-dimethyl-	0.81	Benzeneacetic acid, ethyl ester	0.37
Cyclotrisiloxane, hexamethyl-	0.03	Methane, isocyanato-	0.01	Naphthalene	0.29	Benzoic acid, ethyl ester	0.01
Dextroamphetamine	0.87	Methyl vinyl ketone	0.05	Nonanoic acid, ethyl ester	0.32	Butanal, 2-methyl-	0.04
Diethyl azelate	0.89	n-Propyl acetate	0.05	Oxirane, 2-methyl-2-phenyl-	0.00	Butanal, 3-methyl-	0.03
Dodecanoic acid, ethyl ester	0.16	Octanoic acid	0.21	Pentanedioic acid	0.96	Butyrolactone	0.12
Ethanol	0.03	Oxirane, 3-hydroxypropyl-	0.11	Pentanoic acid, ethyl ester	0.91	Cyclobutanol	0.15
Ethyl 4-acetoxybutanoate	0.01	Pentanoic acid	0.49	Phenol, 4-ethyl-2-methoxy-	0.86	Cycloheptasiloxane, tetradecamethyl-	0.65
Formic acid, 1-methylethyl ester	0.05	Propanoic acid, 2-methyl-	0.05	Phenylethyl Alcohol	0.04	Cyclooctasiloxane, hexadecamethyl-	0.23
Furfural	0.88			Propanoic acid, anhydride	0.98	Cyclopentasiloxane, decamethyl-	0.19
Guanidine, N,N-dimethyl-	0.96			Trichloroacetic acid, dodec-9-ynyl ester	0.02	Decanoic acid, ethyl ester	0.80

Hexanoic acid	0.72	Undecylenic acid	0.02	Diethyl azelate	0.00
Isobutyl acetate	0.01			Dodecanoic acid, ethyl ester	0.21
Isobutyl isopentyl carbonate	0.42			Ethyl Acetate	0.05
Linoleic acid ethyl ester	0.02			gamma-Nonanolactone	0.11
Methane, isocyanato-	0.46			Heptanoic acid, ethyl ester	0.02
n-Propyl acetate	0.00			Hexadecanoic acid, ethyl ester	0.02
Octanoic acid	0.63			Hexanoic acid, 2-methylpropyl ester	0.52
Oxirane, 3-hydroxypropyl-	0.12			Hexanoic acid, ethyl ester	0.00
Pentane, 2,3,4-trimethyl-	0.03			Hexyl methacrylate	0.96
Pentanoic acid	0.04			Isopentyl 4-methylpentanoate	0.03
Pentanoic acid, 2-hydroxy-4-methyl-, ethyl ester	0.00			Isopropyl Alcohol	0.88
Pentanoic acid, 3-methyl-	0.54			Lactic acid	0.02
Phenol, 5-ethenyl-2-	0.72			Naphthalene	0.07

	methoxy-							
	Propanoic acid, 2-hydroxy-, ethyl ester	0.00					Nonanal	0.80
	Propanoic acid, 2-methyl-	0.21					Nonanoic acid, ethyl ester	0.00
							Octanoic acid, ethyl ester	0.00
							Pentadecanoic acid, ethyl ester	0.09
							Pentanoic acid, ethyl ester	0.00
							Propanal, 2-methyl-	0.24
							Sulfurous acid, dipentyl ester	0.23
							Tetradecanoic acid, ethyl ester	0.13
	1,3-Dioxolane, 2,4,5-trimethyl-	0.01	(E)-9-Octadecenoic acid ethyl ester	0.48	1-Butanol, 3-methyl-	0.94	(2-Aziridinylethyl)amine	0.00
Saccharomyces_Unclassified(ITS)	12-Crown-4	0.09	(Z)-4-Decen-1-ol	0.08	2,2'-Bifuran, octahydro-	0.36	13,16-Octadecadiynoic acid, methyl ester	0.07
	17-Octadecynoic acid	0.01	1,3-Dioxan-5-ol	0.12	Acetic acid, diethyl-	0.61	1-Butanol, 3-methyl-	0.05

1-Butanol, 3-methyl-, acetate	0.03	1,3-Dioxolane, 2,4,5-trimethyl-	0.28	Acetic acid, non-3-enyl ester, cis-	0.89	1-Nonanol	0.46
1-Hexanol	0.93	10-Undecen-1-ol	0.75	Benzene, 1-methyl-2-propyl-	0.89	1-Propanol, 2-methyl-	0.04
1-Propanol, 2-methyl-	0.91	1-Butanol, 3-methyl-, acetate	0.01	Benzeneacetaldehyde	0.98	2,3-Butanediol	0.00
2(3H)-Furanone, dihydro-5-pentyl-	0.01	1-Heptanol	0.03	Butyrolactone	0.00	2,4-Di-tert-butylphenol	0.04
2,3-Butanediol, [S-(R*,R*)]-	0.14	1-Hexanol	0.90	Cyclooctasiloxane, hexadecamethyl-	0.27	3-Methyl-hepta-1,6-dien-3-ol	0.04
2-Methoxy-5-methylphenol	0.08	1-Octanol	0.04	Decanoic acid, ethyl ester	0.82	4-Methylcyclohexaneacetic acid	0.28
2-Propenoic acid, 2-methyl-, hexyl ester	0.70	1-Oxa-3,4-diazacyclopentadiene	0.02	Diethyl azelate	0.37	6-Nonynoic acid	0.19
3-Methyl-hepta-1,6-dien-3-ol	0.42	1-Propanol	0.76	Dimethyl ether	0.00	7-Octenoic acid, ethyl ester	0.28
Acetic acid	0.00	2,3-Butanediol, [S-(R*,R*)]-	0.05	Dodecanoic acid, ethyl ester	0.88	8-Methylnonanoic acid, ethyl ester	0.01
Acetic acid ethenyl ester	0.45	2,3-Butanedione	0.96	Ethane, 1,1-diethoxy-	0.00	9(E),11(E)-Conjugated linoleic acid, ethyl ester	0.02
Acetic acid, 2-phenylethyl	0.05	2-Butanol, 3-methyl-,	0.71	Ethene, ethoxy-	0.02	9-Octadecenoic acid,	0.00

ester		(S)-				ethyl ester	
Acetic acid, butyl ester	0.00	2-Methoxy-4-vinylphenol	0.10	Ethyl Acetate	0.33	Acetoin	0.14
Acetic acid, heptyl ester	0.05	Acetic acid	0.00	Formic acid, hexyl ester	0.74	Acetoxyacetic acid, 4-pentadecyl ester	0.23
Acetic acid, hexyl ester	0.02	Acetic acid, 2-phenylethyl ester	0.79	Hexanoic acid, ethyl ester	0.15	Alanine	0.01
Acetic acid, methyl ester	0.02	Acetic acid, butyl ester	0.31	m-Dioxan-4-ol, 2,6-dimethyl-	0.89	alpha-Terpineol	0.04
Acetic acid, nonyl ester	0.34	Acetic acid, hexyl ester	0.03	Oxirane, 2-methyl-2-phenyl-	0.00	Amylene hydrate	0.00
Acetic acid, octyl ester	0.08	Benzeneacetaldehyde, .alpha.-ethylidene-	0.91	Pentanoic acid, ethyl ester	0.23	Azulene	0.01
Acetic acid, pentyl ester	0.53	Benzeneethanol, b-ethyl-	0.24	Phenylethyl Alcohol	0.88	Benzaldehyde	0.04
Acetoin	0.00	Butanedioic acid, diethyl ester	0.00	Propanoic acid, anhydride	0.55	Benzeneacetaldehyde	0.26
Alanine	0.01	Butanoic acid	0.28	Trichloroacetic acid, dodec-9-ynyl ester	0.02	Benzeneacetic acid, ethyl ester	0.37
Benzaldehyde	0.02	Butanoic acid, 3-methyl-	0.07	Undecylenic acid	0.02	Benzoic acid, ethyl ester	0.00
Benzaldehyde, 4-pentyl-	0.03	Cycloheptasiloxane, tetradecamethyl-	0.00			Butanal	0.23

Benzeneacetaldehyde, .alpha.-ethyl-	0.03	Cyclohexasiloxane, dodecamethyl-	0.00	Butanal, 2-methyl-	0.07
Benzeneacetaldehyde, .alpha.-ethylidene-	0.01	Cyclooctasiloxane, hexadecamethyl-	0.02	Butanal, 3-methyl-	0.00
Benzeneacetic acid, ethyl ester	0.05	Cyclopentasiloxane, decamethyl-	0.05	Butyrolactone	0.00
Benzenepropanoic acid, .alpha.-(1-hydroxyethyl)-	0.45	Cyclotetrasiloxane, octamethyl-	0.00	Cyclobutanol	0.61
Benzoic acid, ethyl ester	0.08	Cyclotrisiloxane, hexamethyl-	0.00	Dodecanoic acid, ethyl ester	0.01
Butanal, 3-methyl-	0.09	Decanoic acid, ethyl ester	0.66	Ethane, 1,1-diethoxy-	0.00
Butanedioic acid, diethyl ester	0.78	Diethyl azelate	0.89	Ethyl Acetate	0.01
Butanoic acid	0.00	Ethanol	0.06	gamma-Nonanolactone	0.29
Butanoic acid, 2-methyl-, ethyl ester	0.92	Ethyl 4-acetoxybutanoate	0.12	Heptanoic acid, ethyl ester	0.63
Butanoic acid, 3-methyl-	0.23	Furfural	0.49	Hexadecanoic acid, ethyl ester	0.00
Butanoic acid, ethyl ester	0.01	gamma-Decalactone	0.32	Hexanoic acid, 2-methylpropyl ester	0.03

Creosol	0.02	Heptanoic acid	0.44	Hexanoic acid, ethyl ester	0.24
Cyclobutanol	0.40	Hexanoic acid	0.00	Isopentyl 4-methylpentanoate	0.12
Cycloheptasiloxane, tetradecamethyl-	0.52	Hexyl methacrylate	0.00	Isopropyl Alcohol	0.00
Cyclohexasiloxane, dodecamethyl-	0.59	Isobutyl acetate	0.01	Lactic acid	0.01
Cyclopentasiloxane, decamethyl-	0.10	Linoleic acid ethyl ester	0.46	Naphthalene	0.00
Cyclotetrasiloxane, octamethyl-	0.87	Methane, isocyanato-	0.02	Nonanal	0.09
Cyclotrisiloxane, hexamethyl-	0.32	Methyl vinyl ketone	0.26	Nonanoic acid, ethyl ester	0.87
Dextroamphetamine	0.01	n-Propyl acetate	0.36	Octanoic acid, ethyl ester	0.01
Ethanol	0.62	Octanoic acid	0.01	Pentadecanoic acid, ethyl ester	0.01
Ethyl 4-acetoxybutanoate	0.01	Oxirane, 3-hydroxypropyl-	0.23	Pentanoic acid, ethyl ester	0.85
Formic acid, 1-methylethyl ester	0.46	Pentanoic acid	0.86	Propanal, 2-methyl-	0.00

Furfural	0.10	Propanoic acid, 2-methyl-	0.68			Tetradecanoic acid, ethyl ester	0.00
Guanidine, N,N-dimethyl-	0.09	Sulfurous acid, dipentyl ester	0.02				
Heptanoic acid, ethyl ester	0.95						
Hexadecanoic acid, ethyl ester	0.59						
Hexanoic acid	0.14						
Isobutyl acetate	0.01						
Isobutyl isopentyl carbonate	0.03						
Linoleic acid ethyl ester	0.52						
Methane, isocyanato-	0.63						
Naphthalene	0.80						
Nonanal	0.94						
Nonanoic acid, ethyl ester	0.87						
n-Propyl acetate	0.01						
Octanoic acid	0.12						
Oxirane, 3-hydroxypropyl-	0.55						

	Pentane, 2,3,4-trimethyl-	0.43						
	Pentanedioic acid	0.78						
	Pentanoic acid	0.90						
	Pentanoic acid, 2-hydroxy-4-methyl-, ethyl ester	0.15						
	Pentanoic acid, 3-methyl-	0.05						
	Phenol, 4-ethyl-2-methoxy-	0.45						
	Phenol, 5-ethenyl-2-methoxy-	0.19						
	Propanoic acid, 2-hydroxy-, ethyl ester	0.23						
	Propanoic acid, 2-methyl-	0.32						
Stenotrophomonas_ maltophilia(16S)	1-Butanol, 3-methyl-	0.82	(2-Aziridinylethyl)amine	0.03	1,3-Dioxolane, 2,4,5-trimethyl-	0.00	1,3-Dioxan-5-ol	0.01
	1-Hexanol	0.63	(E)-9-Octadecenoic acid ethyl ester	0.02	12-Crown-4	0.03	1,3-Dioxolane, 2,4,5-trimethyl-	0.00
	2,2'-Bifuran, octahydro-	0.16	(Z)-4-Decen-1-ol	0.04	17-Octadecynoic acid	0.16	1-Butanol, 3-methyl-, acetate	0.01
	Benzeneacetaldehyde	0.47	10-Undecen-1-ol	0.07	1-Butanol, 3-methyl-, acetate	0.00	1-Propanol	0.93

Butanedioic acid, diethyl ester	0.71	13,16-Octadecadiynoic acid, methyl ester	0.07	1-Propanol, 2-methyl-	0.93	2,3-Butanediol, [S-(R*,R*)]-	0.00
Butanoic acid, 2-methyl-, ethyl ester	0.25	1-Butanol, 3-methyl-	0.00	2(3H)-Furanone, dihydro-5-pentyl-	0.00	2,3-Butanedione	0.33
Butyrolactone	0.05	1-Heptanol	0.01	2,3-Butanediol, [S-(R*,R*)]-	0.03	2-Methoxy-4-vinylphenol	0.00
Cyclotetrasiloxane, octamethyl-	0.87	1-Hexanol	0.00	2-Methoxy-5-methylphenol	0.05	9(E),11(E)-Conjugated linoleic acid, ethyl ester	0.67
Decanoic acid, ethyl ester	0.41	1-Nonanol	0.00	2-Propenoic acid, 2-methyl-, hexyl ester	0.17	Acetic acid	0.00
Diethyl azelate	0.01	1-Octanol	0.03	3-Methyl-hepta-1,6-dien-3-ol	0.13	Acetic acid, butyl ester	0.15
Dimethyl ether	0.00	1-Oxa-3,4-diazacyclopentadiene	0.32	Acetic acid	0.00	Acetic acid, hexyl ester	0.14
Dodecanoic acid, ethyl ester	0.51	1-Propanol, 2-methyl-	0.00	Acetic acid ethenyl ester	0.12	Acetoin	0.00
Ethane, 1,1-diethoxy-	0.00	2,3-Butanediol	0.12	Acetic acid, 2-phenylethyl ester	0.83	Benzeneacetaldehyde, .alpha.-ethylidene-	0.02
Ethene, ethoxy-	0.14	2,4-Di-tert-butylphenol	0.00	Acetic acid, butyl ester	0.14	Benzeneethanol, b-ethyl-	0.00
Ethyl Acetate	0.36	2-Butanol, 3-methyl-, (S)-	0.11	Acetic acid, diethyl-	0.69	Butanedioic acid, diethyl ester	0.33

Formic acid, hexyl ester	0.41	3-Methyl-hepta-1,6-dien-3-ol	0.01	Acetic acid, heptyl ester	0.17	Butanoic acid	0.15
Heptanoic acid, ethyl ester	0.26	4-Methylcyclohexaneacetic acid	0.12	Acetic acid, hexyl ester	0.02	Butanoic acid, 3-methyl-	0.00
Hexanoic acid, ethyl ester	0.10	6-Nonynoic acid	0.23	Acetic acid, methyl ester	0.03	Cycloheptasiloxane, tetradecamethyl-	0.48
Nonanoic acid, ethyl ester	0.25	7-Octenoic acid, ethyl ester	0.12	Acetic acid, non-3-enyl ester, cis-	0.15	Cyclohexasiloxane, dodecamethyl-	0.39
Oxirane, 2-methyl-2-phenyl-	0.05	8-Methylnonanoic acid, ethyl ester	0.00	Acetic acid, nonyl ester	0.02	Cyclotrisiloxane, hexamethyl-	0.23
Pentane, 2,3,4-trimethyl-	0.68	9-Octadecenoic acid, ethyl ester	0.01	Acetic acid, octyl ester	0.04	Ethane, 1,1-diethoxy-	0.99
Pentanoic acid, ethyl ester	0.27	Acetic acid, 2-phenylethyl ester	0.00	Acetic acid, pentyl ester	0.96	Ethanol	0.09
Propanoic acid, anhydride	0.37	Acetoxyacetic acid, 4-pentadecyl ester	0.19	Acetoin	0.01	Ethyl 4-acetoxybutanoate	0.00
Trichloroacetic acid, dodec-9-ynyl ester	0.14	Alanine	0.10	Alanine	0.18	Furfural	0.00
Undecylenic acid	0.14	alpha-Terpineol	0.00	Benzaldehyde	0.00	gamma-Decalactone	0.00
		Amylene hydrate	0.00	Benzaldehyde, 4-pentyl-	0.63	Heptanoic acid	0.00

Azulene	0.38	Benzene, 1-methyl-2-propyl-	0.15	Hexanoic acid	0.00
Benzaldehyde	0.08	Benzeneacetaldehyde, .alpha.-ethyl-	0.63	Isobutyl acetate	0.00
Benzeneacetaldehyde	0.00	Benzeneacetaldehyde, .alpha.-ethylidene-	0.18	Methane, isocyanato-	0.00
Benzeneacetic acid, ethyl ester	0.14	Benzeneacetic acid, ethyl ester	0.04	Methyl vinyl ketone	0.01
Benzoic acid, ethyl ester	0.00	Benzenepropanoic acid, .alpha.-(1-hydroxyethyl)-	0.12	n-Propyl acetate	0.13
Butanal	0.19	Benzoic acid, ethyl ester	0.12	Octanoic acid	0.02
Butanal, 2-methyl-	0.36	Butanal, 3-methyl-	0.04	Oxirane, 3-hydroxypropyl-	0.24
Butanal, 3-methyl-	0.00	Butanoic acid	0.43	Pentanoic acid	0.25
Butyrolactone	0.10	Butanoic acid, 3-methyl-	0.04	Propanoic acid, 2-methyl-	0.13
Cyclobutanol	0.40	Butanoic acid, ethyl ester	0.18		
Cyclooctasiloxane, hexadecamethyl-	0.32	Creosol	0.03		

Cyclopentasiloxane, decamethyl-	0.18	Cyclobutanol	0.12
Cyclotetrasiloxane, octamethyl-	0.78	Cycloheptasiloxane, tetradecamethyl-	0.32
Decanoic acid, ethyl ester	0.94	Cyclohexasiloxane, dodecamethyl-	0.25
Diethyl azelate	0.00	Cyclooctasiloxane, hexadecamethyl-	0.75
Dodecanoic acid, ethyl ester	0.00	Cyclopentasiloxane, decamethyl-	0.64
Ethyl Acetate	0.00	Cyclotrisiloxane, hexamethyl-	0.33
gamma-Nonanolactone	0.42	Dextroamphetamine	0.16
Heptanoic acid, ethyl ester	0.00	Ethanol	0.45
Hexadecanoic acid, ethyl ester	0.00	Ethyl 4-acetoxybutanoate	0.00
Hexanoic acid, 2-methylpropyl ester	0.01	Formic acid, 1-methylethyl ester	0.41
Hexanoic acid, ethyl ester	0.00	Furfural	0.24

Hexyl methacrylate	0.73	Guanidine, N,N-dimethyl-	0.53
Isopentyl 4-methylpentanoate	0.03	Hexadecanoic acid, ethyl ester	0.93
Isopropyl Alcohol	0.06	Hexanoic acid	0.25
Lactic acid	0.06	Isobutyl acetate	0.00
Linoleic acid ethyl ester	0.96	Isobutyl isopentyl carbonate	0.63
Naphthalene	0.01	Linoleic acid ethyl ester	0.70
Nonanal	0.34	m-Dioxan-4-ol, 2,6-dimethyl-	0.15
Nonanoic acid, ethyl ester	0.00	Methane, isocyanato-	0.04
Octanoic acid, ethyl ester	0.00	Naphthalene	0.65
Pentadecanoic acid, ethyl ester	0.00	Nonanal	0.10
Pentanoic acid, ethyl ester	0.00	n-Propyl acetate	0.01
Propanal, 2-methyl-	0.04	Octanoic acid	0.41

			Sulfurous acid, dipentyl ester	0.32	Oxirane, 3-hydroxypropyl-	0.04		
			Tetradecanoic acid, ethyl ester	0.01	Pentanedioic acid	0.12		
					Pentanoic acid	0.09		
					Pentanoic acid, 2-hydroxy-4-methyl-, ethyl ester	0.01		
					Pentanoic acid, 3-methyl-	0.17		
					Phenol, 4-ethyl-2-methoxy-	0.12		
					Phenol, 5-ethenyl-2-methoxy-	0.16		
					Phenylethyl Alcohol	0.67		
					Propanoic acid, 2-hydroxy-, ethyl ester	0.03		
					Propanoic acid, 2-methyl-	0.10		
Xanthomonas_sacchari(16S)	1-Butanol, 3-methyl-	0.68	(2-Aziridinylethyl)amine	0.00	1,3-Dioxolane, 2,4,5-trimethyl-	0.00	1,3-Dioxan-5-ol	0.01
	1-Hexanol	0.26	(E)-9-Octadecenoic	0.25	12-Crown-4	0.01	1,3-Dioxolane, 2,4,5-	0.00

		acid ethyl ester				trimethyl-	
1-Propanol, 2-methyl-	0.72	(Z)-4-Decen-1-ol	0.18	17-Octadecynoic acid	0.03	1-Butanol, 3-methyl-, acetate	0.00
2,2'-Bifuran, octahydro-	0.22	10-Undecen-1-ol	0.29	1-Butanol, 3-methyl-, acetate	0.03	2,3-Butanediol, [S-(R*,R*)]-	0.00
Benzeneacetaldehyde	0.70	13,16-Octadecadiynoic acid, methyl ester	0.02	2(3H)-Furanone, dihydro-5-pentyl-	0.00	2,3-Butanedione	0.28
Butanedioic acid, diethyl ester	0.24	1-Butanol, 3-methyl-	0.00	2,3-Butanediol, [S-(R*,R*)]-	0.07	2-Methoxy-4-vinylphenol	0.00
Butanoic acid, 2-methyl-, ethyl ester	0.25	1-Heptanol	0.17	2-Methoxy-5-methylphenol	0.11	Acetic acid	0.00
Butyrolactone	0.03	1-Hexanol	0.00	2-Propenoic acid, 2-methyl-, hexyl ester	0.03	Acetic acid, butyl ester	0.11
Cycloheptasiloxane, tetradecamethyl-	0.88	1-Nonanol	0.00	3-Methyl-hepta-1,6-dien-3-ol	0.04	Acetic acid, hexyl ester	0.05
Cyclooctasiloxane, hexadecamethyl-	0.22	1-Octanol	0.32	Acetic acid	0.00	Acetoin	0.02
Decanoic acid, ethyl ester	0.39	1-Oxa-3,4-diazacyclopentadiene	0.48	Acetic acid ethenyl ester	0.04	Benzeneacetaldehyde, .alpha.-ethylidene-	0.08
Diethyl azelate	0.00	1-Propanol	0.87	Acetic acid, 2-phenylethyl ester	0.39	Benzeneethanol, b-ethyl-	0.00

Dimethyl ether	0.00	1-Propanol, 2-methyl-	0.00	Acetic acid, butyl ester	0.00	Butanedioic acid, diethyl ester	0.04
Dodecanoic acid, ethyl ester	0.21	2,3-Butanediol	0.03	Acetic acid, diethyl-	0.61	Butanoic acid	0.23
Ethane, 1,1-diethoxy-	0.00	2,4-Di-tert-butylphenol	0.00	Acetic acid, heptyl ester	0.04	Butanoic acid, 3-methyl-	0.00
Ethene, ethoxy-	0.08	2-Butanol, 3-methyl-, (S)-	0.08	Acetic acid, hexyl ester	0.05	Cycloheptasiloxane, tetradecamethyl-	0.11
Formic acid, 1-methylethyl ester	0.76	3-Methyl-hepta-1,6-dien-3-ol	0.00	Acetic acid, methyl ester	0.00	Cyclohexasiloxane, dodecamethyl-	0.06
Formic acid, hexyl ester	0.70	4-Methylcyclohexaneacetic acid	0.12	Acetic acid, non-3-enyl ester, cis-	0.15	Cyclotetrasiloxane, octamethyl-	0.87
Heptanoic acid, ethyl ester	0.25	6-Nonynoic acid	0.09	Acetic acid, nonyl ester	0.02	Cyclotrisiloxane, hexamethyl-	0.03
Hexanoic acid, ethyl ester	0.01	7-Octenoic acid, ethyl ester	0.12	Acetic acid, octyl ester	0.01	Decanoic acid, ethyl ester	0.64
Linoleic acid ethyl ester	0.66	8-Methylnonanoic acid, ethyl ester	0.00	Acetic acid, pentyl ester	0.19	Ethanol	0.02
Nonanoic acid, ethyl ester	0.29	9(E),11(E)-Conjugated linoleic acid, ethyl ester	0.96	Acetoin	0.00	Ethyl 4-acetoxybutanoate	0.00
Oxirane, 2-methyl-2-phenyl-	0.03	9-Octadecenoic acid, ethyl ester	0.00	Alanine	0.03	Furfural	0.00

Pentane, 2,3,4-trimethyl-	0.52	Acetic acid, 2-phenylethyl ester	0.02	Benzaldehyde	0.00	gamma-Decalactone	0.00
Pentanoic acid, ethyl ester	0.16	Acetoxyacetic acid, 4-pentadecyl ester	0.15	Benzaldehyde, 4-pentyl-	0.16	Heptanoic acid	0.00
Propanoic acid, anhydride	0.08	Alanine	0.02	Benzene, 1-methyl-2-propyl-	0.15	Hexanoic acid	0.00
Trichloroacetic acid, dodec-9-ynyl ester	0.08	alpha-Terpineol	0.00	Benzeneacetaldehyde, .alpha.-ethyl-	0.16	Isobutyl acetate	0.00
Undecylenic acid	0.08	Amylene hydrate	0.00	Benzeneacetaldehyde, .alpha.-ethylidene-	0.03	Methane, isocyanato-	0.00
		Azulene	0.11	Benzeneacetic acid, ethyl ester	0.02	Methyl vinyl ketone	0.01
		Benzaldehyde	0.02	Benzenepropanoic acid, .alpha.-(1-hydroxyethyl)-	0.04	n-Propyl acetate	0.09
		Benzeneacetaldehyde	0.00	Benzoic acid, ethyl ester	0.02	Octanoic acid	0.00
		Benzeneacetic acid, ethyl ester	0.14	Butanal, 3-methyl-	0.01	Oxirane, 3-hydroxypropyl-	0.14
		Benzoic acid, ethyl ester	0.00	Butanoic acid	0.03	Pentanoic acid	0.32

Butanal	0.15	Butanoic acid, 3-methyl-	0.00	Propanoic acid, 2-methyl-	0.31
Butanal, 2-methyl-	0.18	Butanoic acid, ethyl ester	0.03		
Butanal, 3-methyl-	0.00	Creosol	0.00		
Butyrolactone	0.01	Cyclobutanol	0.03		
Cyclobutanol	0.28	Cyclohexasiloxane, dodecamethyl-	0.69		
Cyclooctasiloxane, hexadecamethyl-	0.48	Cyclopentasiloxane, decamethyl-	0.04		
Cyclopentasiloxane, decamethyl-	0.59	Cyclotetrasiloxane, octamethyl-	0.97		
Diethyl azelate	0.00	Cyclotrisiloxane, hexamethyl-	0.89		
Dodecanoic acid, ethyl ester	0.00	Dextroamphetamine	0.03		
Ethane, 1,1-diethoxy-	0.55	Ethanol	0.90		
Ethyl Acetate	0.00	Ethyl 4-acetoxybutanoate	0.00		
gamma-Nonanolactone	0.23	Ethyl Acetate	0.97		

Heptanoic acid, ethyl ester	0.02	Furfural	0.07
Hexadecanoic acid, ethyl ester	0.00	Guanidine, N,N-dimethyl-	0.12
Hexanoic acid, 2-methylpropyl ester	0.00	Hexadecanoic acid, ethyl ester	0.88
Hexanoic acid, ethyl ester	0.00	Hexanoic acid	0.03
Hexyl methacrylate	0.97	Isobutyl acetate	0.00
Isopentyl 4-methylpentanoate	0.01	Isobutyl isopentyl carbonate	0.16
Isopropyl Alcohol	0.01	m-Dioxan-4-ol, 2,6-dimethyl-	0.15
Lactic acid	0.01	Methane, isocyanato-	0.02
Linoleic acid ethyl ester	0.96	Naphthalene	0.24
Naphthalene	0.00	Nonanal	0.12
Nonanal	0.15	n-Propyl acetate	0.01
Nonanoic acid, ethyl ester	0.00	Octanoic acid	0.03

Octanoic acid, ethyl ester	0.00	Oxirane, 3-hydroxypropyl-	0.07
Pentadecanoic acid, ethyl ester	0.00	Pentanedioic acid	0.15
Pentanoic acid, ethyl ester	0.00	Pentanoic acid	0.62
Propanal, 2-methyl-	0.00	Pentanoic acid, 2-hydroxy-4-methyl-, ethyl ester	0.06
Sulfurous acid, dipentyl ester	0.48	Pentanoic acid, 3-methyl-	0.04
Tetradecanoic acid, ethyl ester	0.00	Phenol, 4-ethyl-2-methoxy-	0.04
		Phenol, 5-ethenyl-2-methoxy-	0.04
		Phenylethyl Alcohol	0.31
		Propanoic acid, 2-hydroxy-, ethyl ester	0.16
		Propanoic acid, 2-methyl-	0.02

Supplementary Table 11. Correlation table between core microorganisms (relative abundance greater than 10%) and organic acids in acetic acid fermentation under traditional inoculation and direct injection inoculation.

Core microorganisms	positive				negative			
	Traditional inoculation		Direct inoculation		Traditional inoculation		Direct inoculation	
	Volatile flavor substances	pvalue	Volatile flavor substances	pvalue	Volatile flavor substances	pvalue	Volatile flavor substances	pvalue
Acetobacter_pasteurianus(16S)	acetic acid	0.00	acetic acid	0.00	malic acid	0.52	succinic acid	0.16
	citric acid	0.07	citric acid	0.00	tartaric acid	0.09		
	lactic acid	0.00	lactic acid	0.09				
	oxalic acid	0.60	malic acid	0.00				
	pyroglutamic acid	0.02	oxalic acid	0.30				
	pyruvic acid	0.00	pyroglutamic acid	0.00				
	succinic acid	0.07	pyruvic acid	0.00				
			tartaric acid	0.17				
Agrobacterium_larrymoorei(16S)	malic acid	0.72	oxalic acid	0.26	acetic acid	0.00	acetic acid	0.00
	tartaric acid	0.00	succinic acid	0.00	citric acid	0.07	citric acid	0.01
					lactic acid	0.00	lactic acid	0.03
					oxalic acid	0.54	malic acid	0.00

					pyroglutamic acid	0.00	pyroglutamic acid	0.00
					pyruvic acid	0.00	pyruvic acid	0.18
					succinic acid	0.23	tartaric acid	0.82
	acetic acid	0.14			citric acid	0.02	acetic acid	0.52
	lactic acid	0.45			malic acid	0.13	citric acid	0.22
	pyruvic acid	0.17			oxalic acid	0.00	lactic acid	0.80
					pyroglutamic acid	0.94	malic acid	0.13
Alternaria_Unclassified(ITS)					succinic acid	0.09	oxalic acid	0.13
					tartaric acid	0.02	pyroglutamic acid	0.98
							pyruvic acid	0.57
							succinic acid	0.18
							tartaric acid	0.34
	acetic acid	0.05	acetic acid	0.00	tartaric acid	0.05	oxalic acid	0.10
Lactobacillus_acetotolerans(16S)	citric acid	0.00	citric acid	0.19			succinic acid	0.00
	lactic acid	0.02	lactic acid	0.01			tartaric acid	0.83
	malic acid	0.02	malic acid	0.02				

	oxalic acid	0.01	pyroglutamic acid	0.00				
	pyroglutamic acid	0.00	pyruvic acid	0.49				
	pyruvic acid	0.04						
	succinic acid	0.00						
Saccharomyces_Unclassified(ITS)	acetic acid	0.00	acetic acid	0.01	tartaric acid	0.01	succinic acid	0.51
	citric acid	0.12	citric acid	0.04			tartaric acid	0.59
	lactic acid	0.00	lactic acid	0.49				
	malic acid	0.92	malic acid	0.00				
	oxalic acid	0.82	oxalic acid	0.64				
	pyroglutamic acid	0.00	pyroglutamic acid	0.07				
	pyruvic acid	0.00	pyruvic acid	0.04				
	succinic acid	0.03						
Stenotrophomonas_maltophilia(16S)	malic acid	0.81	oxalic acid	0.51	acetic acid	0.00	acetic acid	0.00
	tartaric acid	0.01	succinic acid	0.00	citric acid	0.02	citric acid	0.01
					lactic acid	0.00	lactic acid	0.06
					oxalic acid	0.14	malic acid	0.00
					pyroglutamic	0.00	pyroglutamic	0.00

					acid		acid	
					pyruvic acid	0.00	pyruvic acid	0.09
					succinic acid	0.04	tartaric acid	0.72
Xanthomonas_sacchari(16S)	malic acid	0.12	succinic acid	0.01	acetic acid	0.00	acetic acid	0.00
	oxalic acid	0.85			citric acid	0.28	citric acid	0.00
	tartaric acid	0.00			lactic acid	0.00	lactic acid	0.03
					pyroglutamic acid	0.00	malic acid	0.00
					pyruvic acid	0.00	oxalic acid	0.95
					succinic acid	0.43	pyroglutamic acid	0.00
							pyruvic acid	0.02
							tartaric acid	0.55

Supplementary Table 12. Correlation table between core microorganisms (relative abundance greater than 10%) and amino acids in acetic acid fermentation under traditional inoculation and direct injection inoculation.

Core microorganisms	positive		negative	
	Traditional inoculation	Direct inoculation	Traditional inoculation	Direct inoculation

	Volatile flavor substances	pvalue	Volatile flavor substances	pvalue	Volatile flavor substances	pvalue	Volatile flavor substances	pvalue
Acetobacter_pasteurianus(16S)	Ala	0.00	Ala	0.00	3Mehis	0.01	a-AAA	0.23
	Gly	0.07	Gly	0.00	Glu	0.57	Glu	0.00
	Lys	0.01	Lys	0.00			Trp	0.00
	a-AAA	0.07	3Mehis	0.01				
	a-ABA	0.00	a-ABA	0.00				
	Arg	0.00	Arg	0.00				
	Asn	0.00	Asn	0.00				
	Asp	0.00	Asp	0.00				
	Cys	0.01	Cys	0.00				
	GABA	0.15	GABA	0.82				
	His	0.01	His	0.23				
	Ile	0.00	Ile	0.00				
	Leu	0.19	Leu	0.01				
	Met	0.06	Met	0.00				
	Orn	0.69	Orn	0.00				
	PEA	0.03	PEA	0.00				

	Phe	0.63	Phe	0.02				
	P-ser	0.00	P-ser	0.10				
	Ser	0.08	Ser	0.00				
	Tau	0.18	Tau	0.01				
	Thr	0.00	Thr	0.00				
	Trp	0.01	Tyr	0.00				
	Tyr	0.27	Val	0.00				
	Val	0.27						
	3Mehis	0.00	a-AAA	0.06	Ala	0.00	Ala	0.00
	Glu	0.40	GABA	0.43	Gly	0.00	Gly	0.00
	Phe	0.05	Glu	0.00	Lys	0.00	Lys	0.00
	Tyr	0.32	His	0.87	a-AAA	0.44	3Mehis	0.10
	Val	0.69	Trp	0.00	a-ABA	0.00	a-ABA	0.00
Agrobacterium_larrymoorei(16S)								
					Arg	0.00	Arg	0.00
					Asn	0.00	Asn	0.00
					Asp	0.00	Asp	0.00
					Cys	0.00	Cys	0.08
					GABA	0.29	Ile	0.00

					His	0.05	Leu	0.14
					Ile	0.00	Met	0.00
					Leu	0.45	Orn	0.00
					Met	0.04	PEA	0.00
					Orn	0.59	Phe	0.35
					PEA	0.00	P-ser	0.41
					P-ser	0.00	Ser	0.00
					Ser	0.01	Tau	0.00
					Tau	0.55	Thr	0.00
					Thr	0.00	Tyr	0.00
					Trp	0.00	Val	0.06
Alternaria_Unclassified(ITS)	Ala	0.09	a-AAA	0.75	3Mehis	0.02	Ala	0.62
	Gly	0.00	Tau	0.64	GABA	0.94	Gly	0.36
	Lys	0.43			Glu	0.07	Lys	0.21
	a-AAA	0.26			Leu	0.07	3Mehis	0.23
	a-ABA	0.10			Orn	0.27	a-ABA	0.42
	Arg	0.14			Phe	0.28	Arg	0.19

Asn	0.22	Tyr	0.01	Asn	0.18
Asp	0.12			Asp	0.26
Cys	0.46			Cys	0.33
His	0.50			GABA	0.12
Ile	0.31			Glu	0.31
Met	0.34			His	0.21
PEA	0.34			Ile	0.52
P-ser	0.19			Leu	0.06
Ser	0.28			Met	0.53
Tau	0.50			Orn	0.80
Thr	0.26			PEA	0.88
Trp	0.01			Phe	0.00
Val	0.73			P-ser	0.17
				Ser	0.22
				Thr	0.20
				Trp	0.77
				Tyr	0.37
				Val	0.34

	Ala	0.04	Ala	0.00	3Mehis	0.50	a-AAA	0.48
	Gly	0.13	Gly	0.01	a-AAA	0.55	GABA	0.88
	Lys	0.01	Lys	0.02	GABA	0.36	Glu	0.00
	a-ABA	0.05	3Mehis	0.25	Phe	0.15	His	0.71
	Arg	0.00	a-ABA	0.00	Tau	0.08	Trp	0.01
	Asn	0.02	Arg	0.03				
	Asp	0.04	Asn	0.01				
	Cys	0.00	Asp	0.00				
	Glu	0.48	Cys	0.38				
Lactobacillus_acetotolerans(16S)	His	0.99	Ile	0.01				
	Ile	0.02	Leu	0.94				
	Leu	0.00	Met	0.01				
	Met	0.05	Orn	0.00				
	Orn	0.86	PEA	0.02				
	PEA	0.01	Phe	0.94				
	P-ser	0.00	P-ser	0.68				
	Ser	0.01	Ser	0.00				
	Thr	0.02	Tau	0.00				

	Trp	0.85	Thr	0.00				
	Tyr	0.67	Tyr	0.02				
	Val	0.21	Val	0.26				
Saccharomyces_Unclassified(ITS)	Ala	0.00	Ala	0.00	3Mehis	0.01	GABA	0.33
	Gly	0.00	Gly	0.00	GABA	0.94	Glu	0.32
	Lys	0.00	Lys	0.00	Glu	0.52	P-ser	0.79
	a-AAA	0.06	3Mehis	0.00	Phe	0.44	Tau	0.76
	a-ABA	0.00	a-AAA	0.96	Tyr	0.61	Trp	0.01
	Arg	0.00	a-ABA	0.00				
	Asn	0.00	Arg	0.00				
	Asp	0.00	Asn	0.00				
	Cys	0.00	Asp	0.00				
	His	0.07	Cys	0.00				
	Ile	0.00	His	0.08				
	Leu	0.13	Ile	0.00				
	Met	0.08	Leu	0.02				
	Orn	0.80	Met	0.00				

	PEA	0.00	Orn	0.00				
	P-ser	0.00	PEA	0.06				
	Ser	0.02	Phe	0.00				
	Tau	0.66	Ser	0.00				
	Thr	0.00	Thr	0.00				
	Trp	0.07	Tyr	0.00				
	Val	0.02	Val	0.00				
	3Mehis	0.00	a-AAA	0.05	Ala	0.00	Ala	0.00
	Glu	0.12	GABA	0.15	Gly	0.01	Gly	0.00
	Phe	0.04	Glu	0.00	Lys	0.01	Lys	0.00
	Tyr	0.58	His	0.64	a-AAA	0.50	3Mehis	0.20
			Trp	0.00	a-ABA	0.00	a-ABA	0.00
Stenotrophomonas_maltophilia(16S)					Arg	0.00	Arg	0.00
					Asn	0.00	Asn	0.00
					Asp	0.00	Asp	0.00
					Cys	0.00	Cys	0.10
					GABA	0.83	Ile	0.00

					His	0.42	Leu	0.48
					Ile	0.00	Met	0.00
					Leu	0.34	Orn	0.00
					Met	0.06	PEA	0.00
					Orn	0.89	Phe	0.28
					PEA	0.00	P-ser	0.68
					P-ser	0.00	Ser	0.00
					Ser	0.03	Tau	0.01
					Tau	0.86	Thr	0.00
					Thr	0.00	Tyr	0.00
					Trp	0.05	Val	0.10
					Val	0.78		
	3Mehis	0.00	a-AAA	0.07	Ala	0.00	Ala	0.00
	Glu	0.03	GABA	0.13	Gly	0.00	Gly	0.00
Xanthomonas_sacchari(16S)	Phe	0.26	Glu	0.00	Lys	0.00	Lys	0.00
	Tyr	0.21	Trp	0.00	a-AAA	0.05	3Mehis	0.05
	Val	0.89			a-ABA	0.00	a-ABA	0.00

Arg	0.00	Arg	0.00
Asn	0.00	Asn	0.00
Asp	0.00	Asp	0.00
Cys	0.00	Cys	0.01
GABA	0.11	His	0.93
His	0.08	Ile	0.00
Ile	0.00	Leu	0.09
Leu	0.81	Met	0.00
Met	0.02	Orn	0.00
Orn	0.45	PEA	0.00
PEA	0.00	Phe	0.13
P-ser	0.00	P-ser	0.70
Ser	0.01	Ser	0.00
Tau	0.13	Tau	0.03
Thr	0.00	Thr	0.00
Trp	0.00	Tyr	0.00
		Val	0.01
